# Supplementary figures and images for: The novel role of Kallistatin in linking metabolic syndromes and cognitive memory deterioration by inducing amyloid-β plaques accumulation and tau protein hyperphosphorylation (part 1 of 2)
Source: eLife. 2025 Aug 5;13:RP99462. doi: 10.7554/eLife.99462 (PMC12324742; doi:10.7554/eLife.99462)

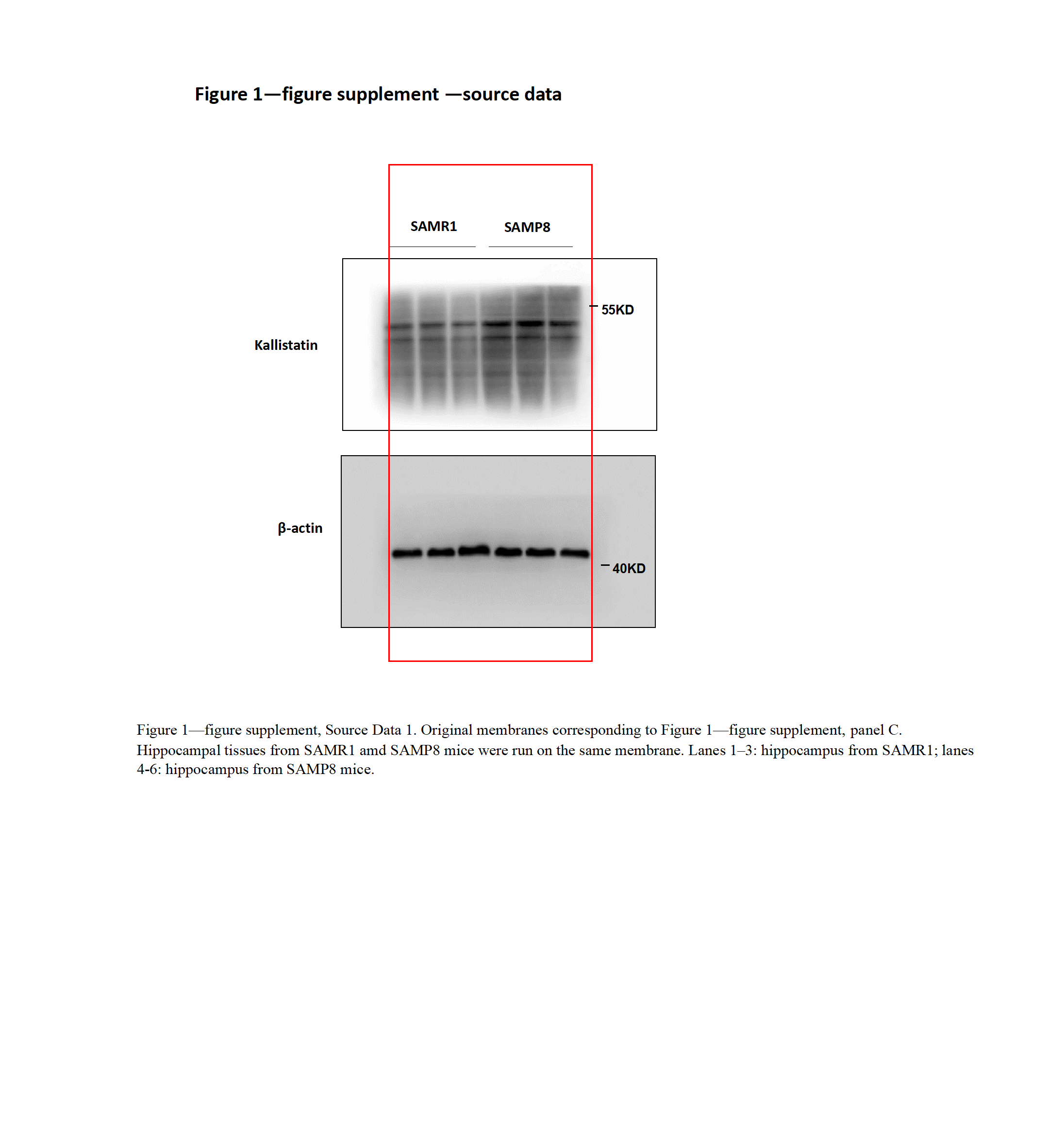

Supplement: Figure 1—figure supplement 1—source data 1. — Western blot analysis of Kallistatin expression in aging model SAMP8 and corresponding control SAMR1 mice hippocampal tissue samples. [file elife-99462-fig1-figsupp1-data1.zip › Figure 1-figure supplement-source data 1/Figure 1-figure supplement source data.png]

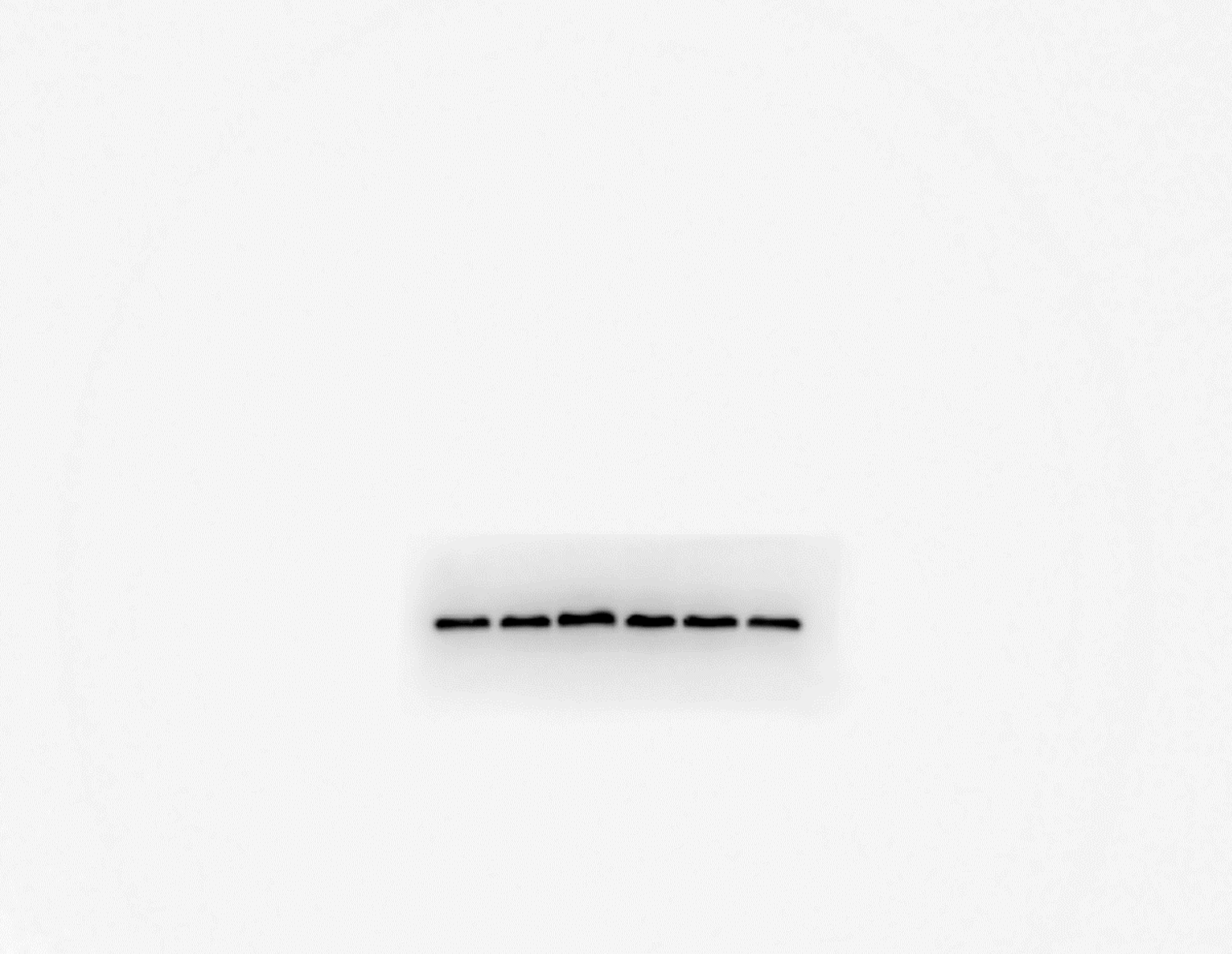

Supplement: Figure 1—figure supplement 1—source data 2. — Western blot analysis of Kallistatin expression in aging model SAMP8 and corresponding control SAMR1 mice hippocampal tissue samples. [file elife-99462-fig1-figsupp1-data2.zip › Fig.S1C Actin hippo.tif]

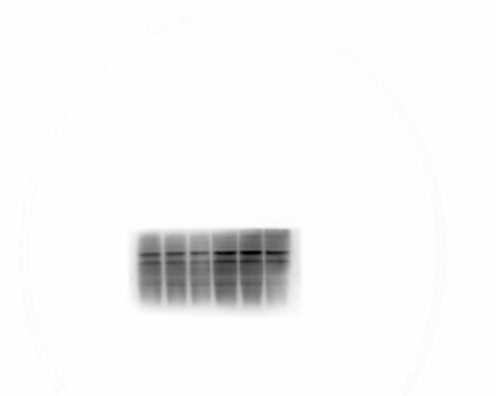

Supplement: Figure 1—figure supplement 1—source data 2. — Western blot analysis of Kallistatin expression in aging model SAMP8 and corresponding control SAMR1 mice hippocampal tissue samples. [file elife-99462-fig1-figsupp1-data2.zip › Fig.S1C KAL hippo.tif]

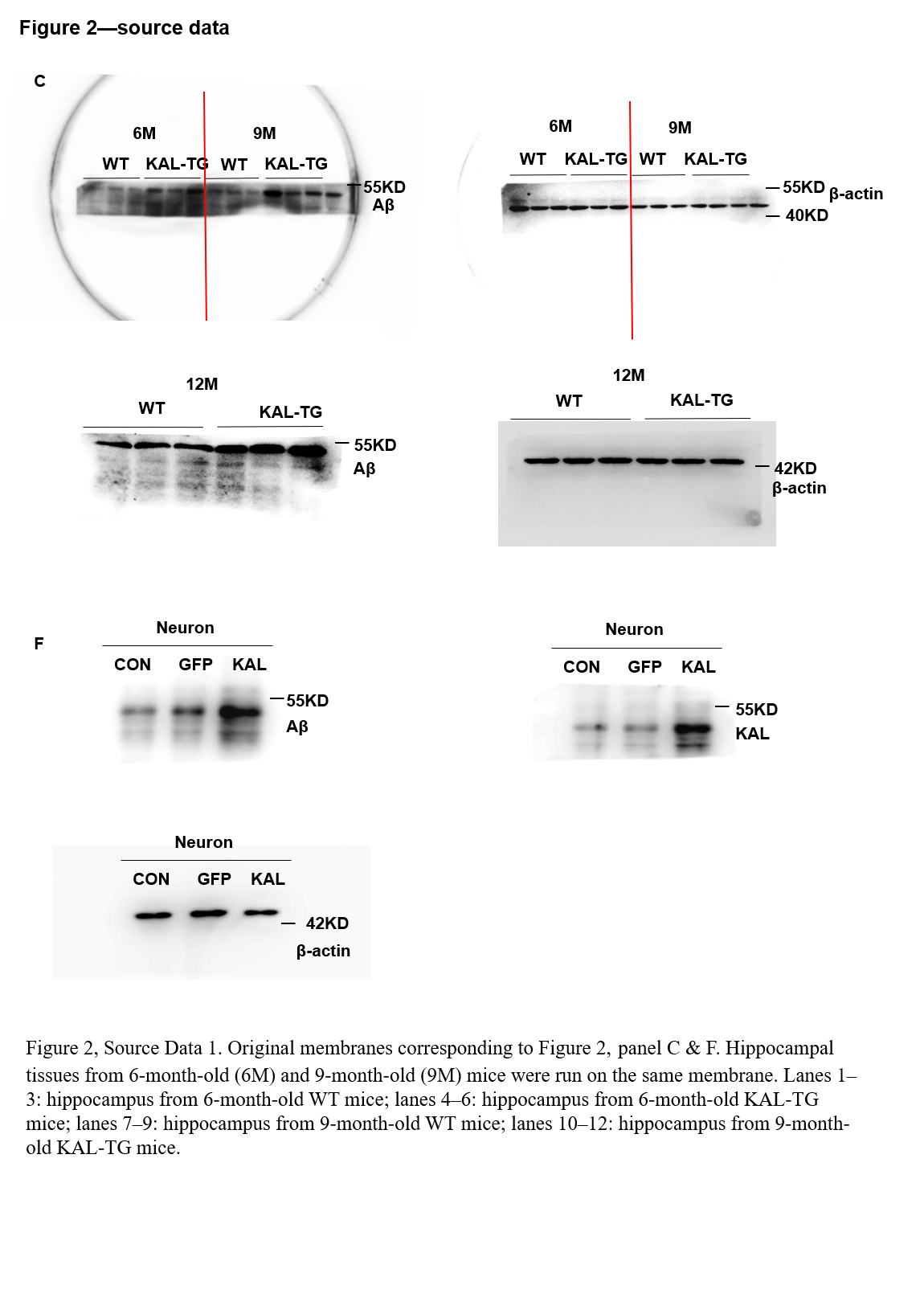

Supplement: Figure 2—source data 1. — Protein levels of Aβ were tested by western blot analysis in hippocampal tissue, n = 3 per group. [file elife-99462-fig2-data1.zip › Figure 2-source data 1/Figure 2-source data.png]

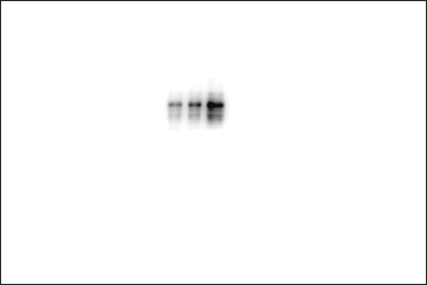

Supplement: Figure 2—source data 2. — Western blot analysis of Aβ protein level in primary hippocampal neurons infected with overexpressing Kallistatin adenovirus and control groups, n = 3 per group. [file elife-99462-fig2-data2.zip › Fig.2F Aα╕åα╕ó.tif]

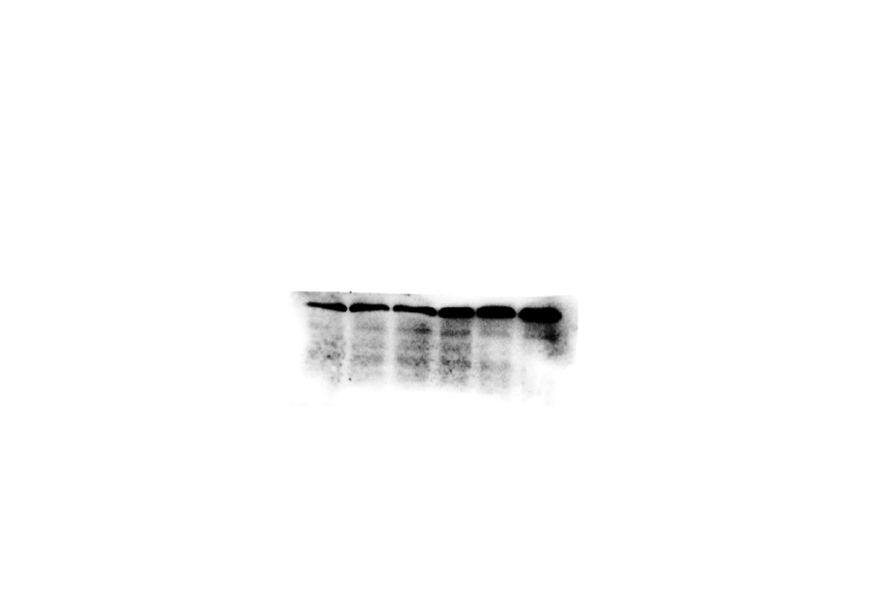

Supplement: Figure 2—source data 2. — Western blot analysis of Aβ protein level in primary hippocampal neurons infected with overexpressing Kallistatin adenovirus and control groups, n = 3 per group. [file elife-99462-fig2-data2.zip › Fig.2C AK╠oA╠éG╠oZ╠î12month.tif]

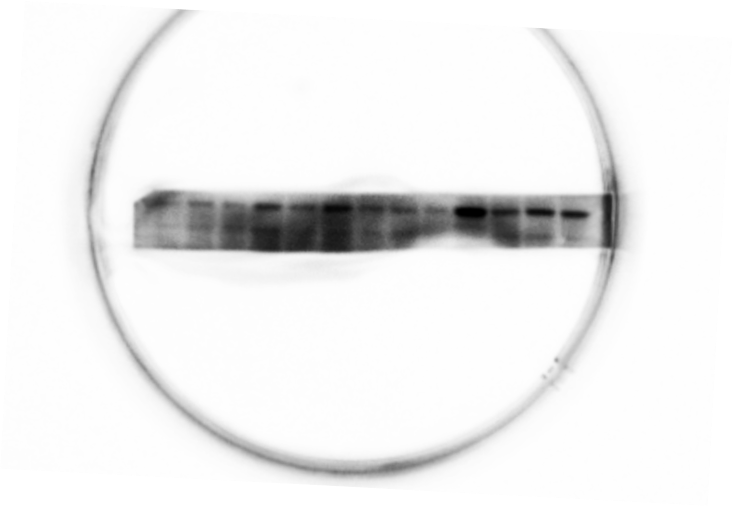

Supplement: Figure 2—source data 2. — Western blot analysis of Aβ protein level in primary hippocampal neurons infected with overexpressing Kallistatin adenovirus and control groups, n = 3 per group. [file elife-99462-fig2-data2.zip › Fig.2C AK╠oA╠éG╠oZ╠î6+9 month.tif]

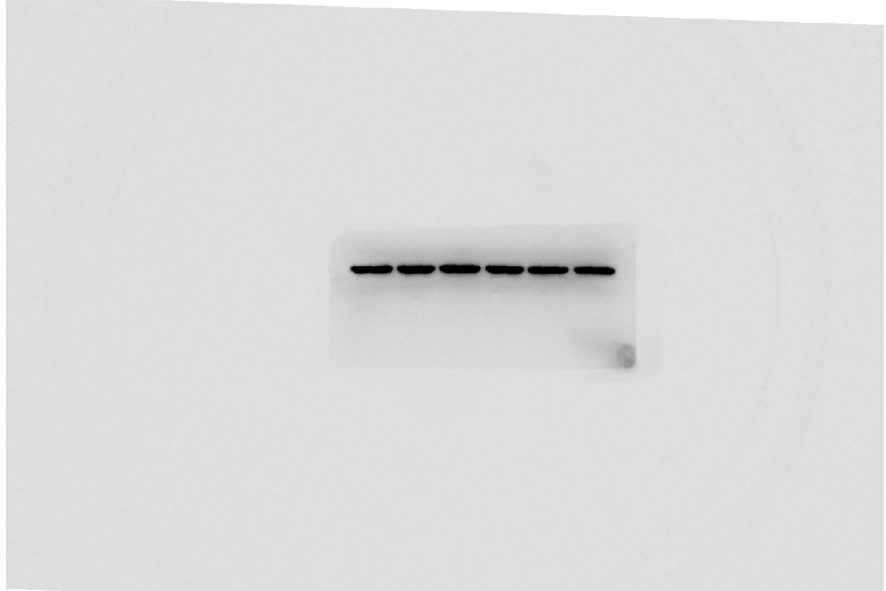

Supplement: Figure 2—source data 2. — Western blot analysis of Aβ protein level in primary hippocampal neurons infected with overexpressing Kallistatin adenovirus and control groups, n = 3 per group. [file elife-99462-fig2-data2.zip › Fig.2C actinG╠oZ╠î12month.tif]

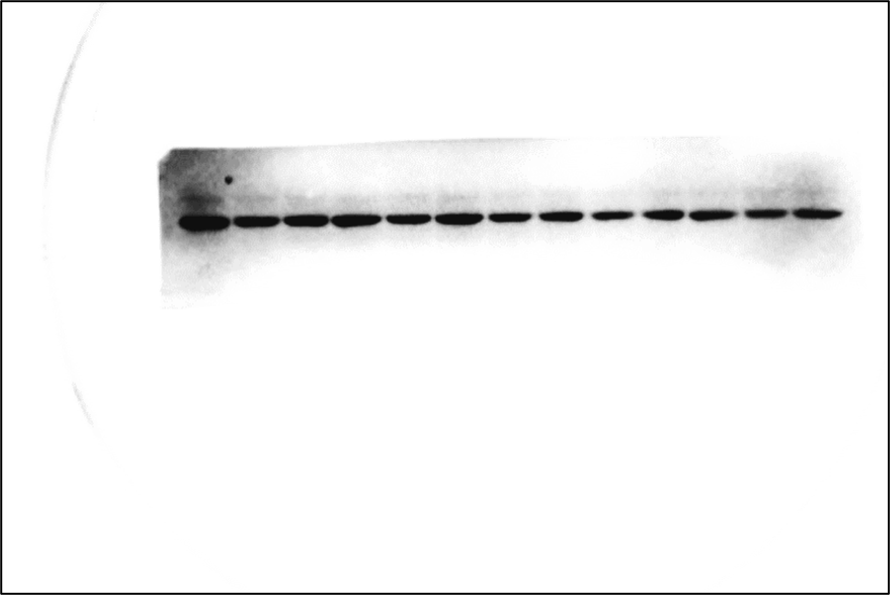

Supplement: Figure 2—source data 2. — Western blot analysis of Aβ protein level in primary hippocampal neurons infected with overexpressing Kallistatin adenovirus and control groups, n = 3 per group. [file elife-99462-fig2-data2.zip › Fig.2C actinG╠oZ╠î6+9 month.tif]

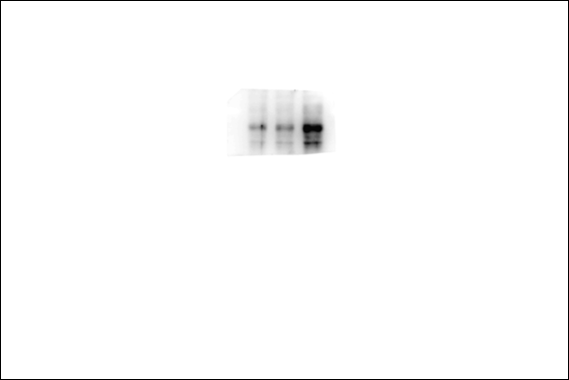

Supplement: Figure 2—source data 2. — Western blot analysis of Aβ protein level in primary hippocampal neurons infected with overexpressing Kallistatin adenovirus and control groups, n = 3 per group. [file elife-99462-fig2-data2.zip › Fig.2F KAL.tif]

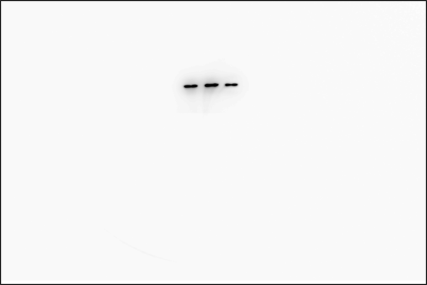

Supplement: Figure 2—source data 2. — Western blot analysis of Aβ protein level in primary hippocampal neurons infected with overexpressing Kallistatin adenovirus and control groups, n = 3 per group. [file elife-99462-fig2-data2.zip › Fig.2F actin.tif]

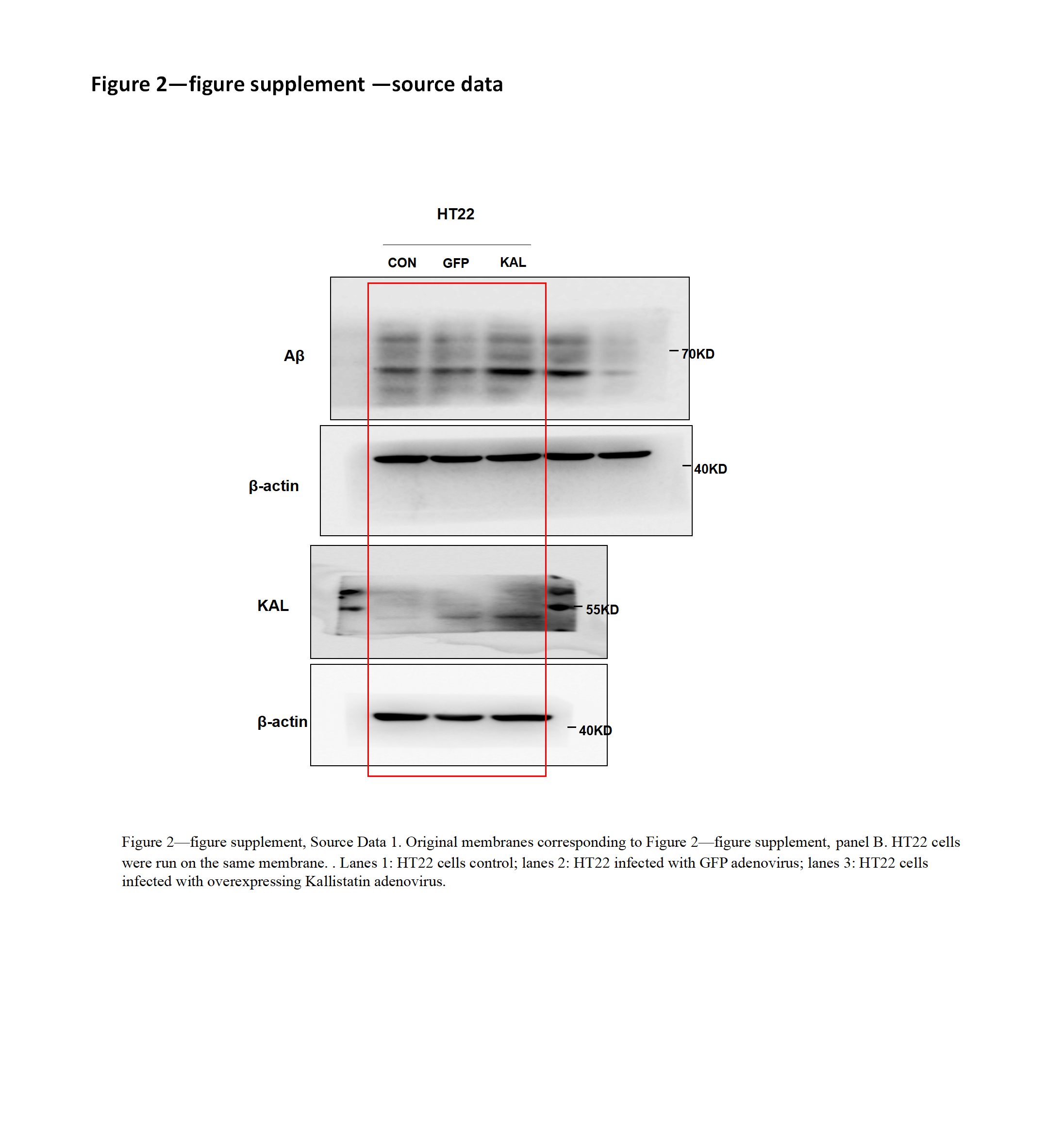

Supplement: Figure 2—figure supplement 1—source data 1. — Western blot analysis of Aβ protein level in HT22 cells infected with overexpressing Kallistatin adenovirus and control groups for 48 hr. [file elife-99462-fig2-figsupp1-data1.zip › Figure 2-figure supplement-source data 1/Figure 2-figure supplement source data.png]

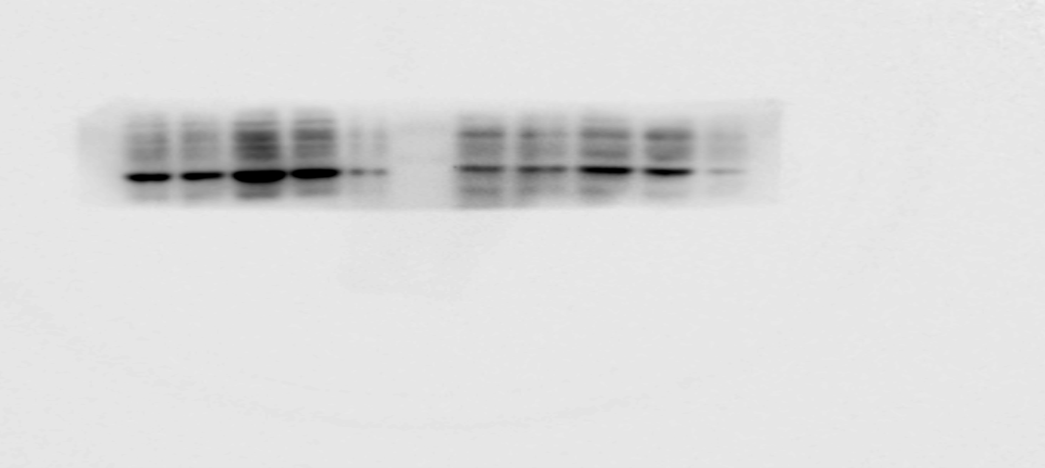

Supplement: Figure 2—figure supplement 1—source data 2. — Western blot analysis of Aβ protein level in HT22 cells infected with overexpressing Kallistatin adenovirus and control groups for 48 hr. [file elife-99462-fig2-figsupp1-data2.zip › Fig.S2C Aα╕åα╕ó HT22.tif]

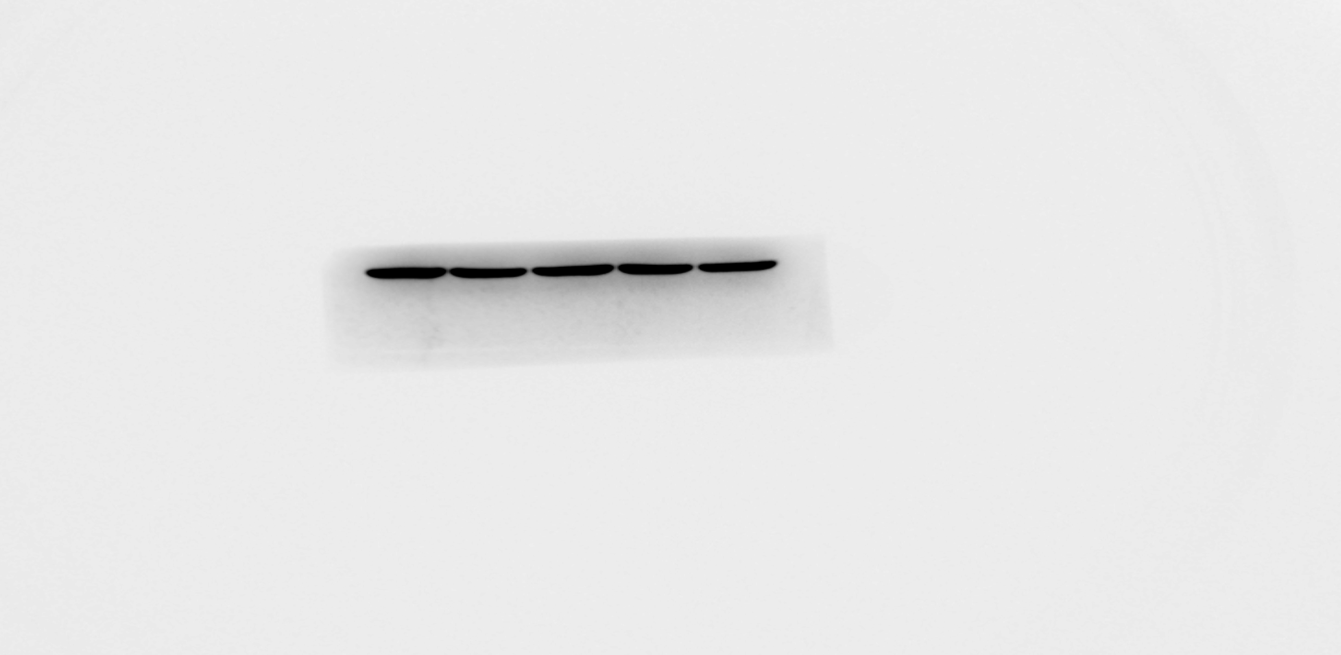

Supplement: Figure 2—figure supplement 1—source data 2. — Western blot analysis of Aβ protein level in HT22 cells infected with overexpressing Kallistatin adenovirus and control groups for 48 hr. [file elife-99462-fig2-figsupp1-data2.zip › Fig.S2C Actin HT22.tif]

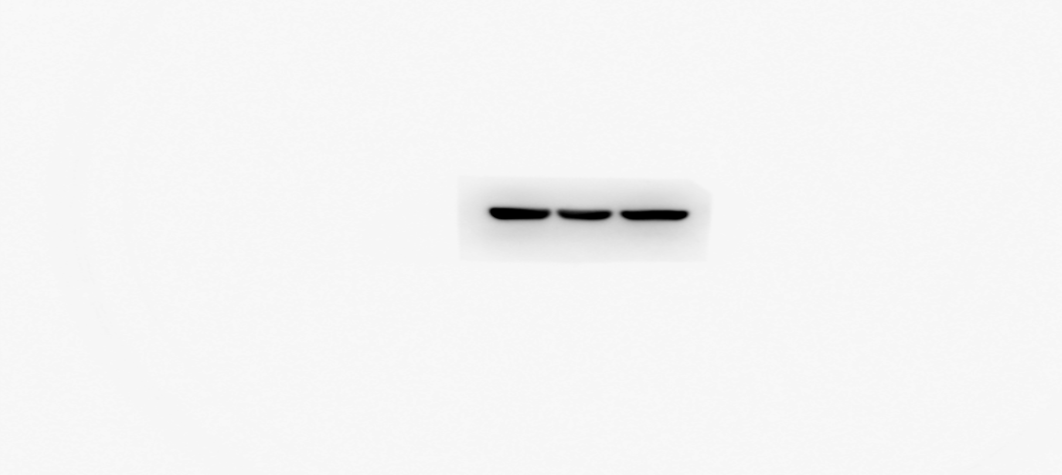

Supplement: Figure 2—figure supplement 1—source data 2. — Western blot analysis of Aβ protein level in HT22 cells infected with overexpressing Kallistatin adenovirus and control groups for 48 hr. [file elife-99462-fig2-figsupp1-data2.zip › Fig.S2C Actin2 HT22.tif]

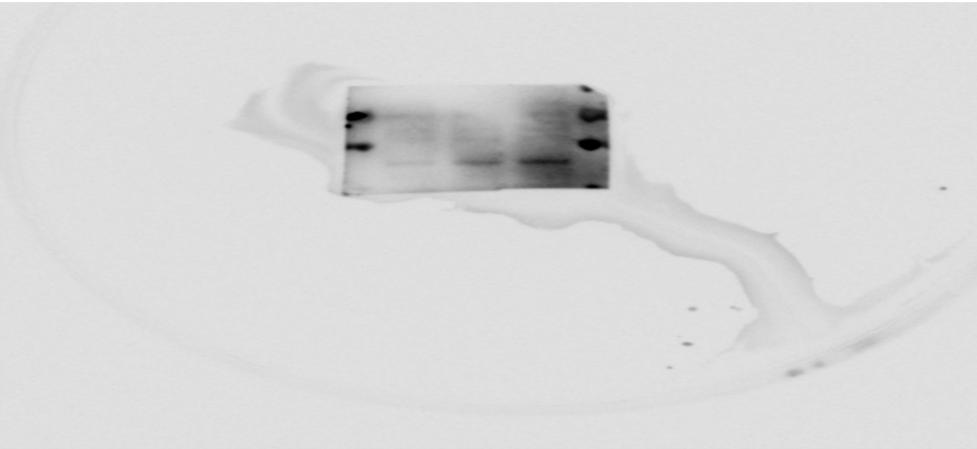

Supplement: Figure 2—figure supplement 1—source data 2. — Western blot analysis of Aβ protein level in HT22 cells infected with overexpressing Kallistatin adenovirus and control groups for 48 hr. [file elife-99462-fig2-figsupp1-data2.zip › Fig.S2C KALT22.tif]

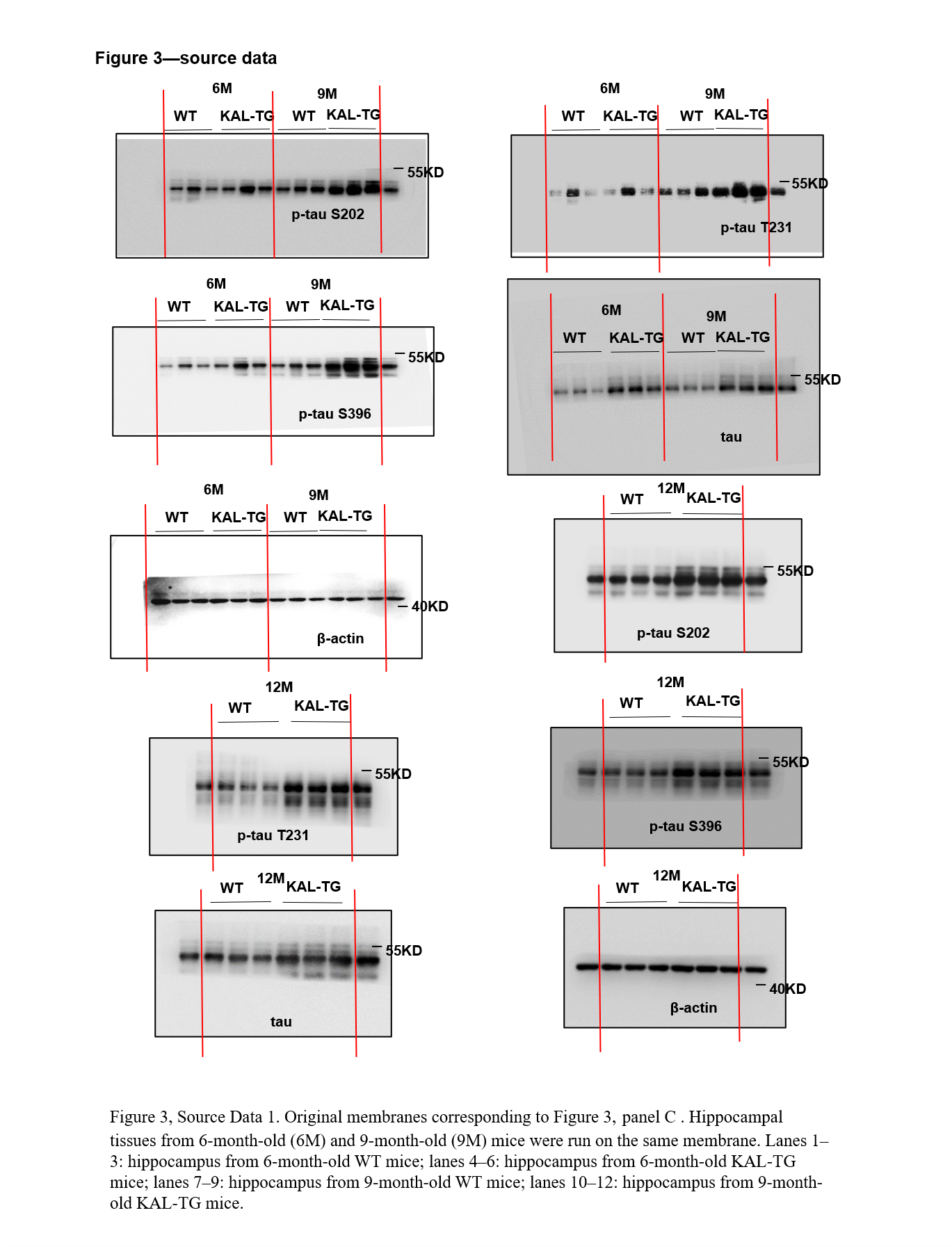

Supplement: Figure 3—source data 1. — Protein levels of phosphorylated tau (p-tau S396, p-tau T231, and p-tau S202) and tau were tested by western blot analysis in hippocampal tissue. [file elife-99462-fig3-data1.zip › Figure 3-source data 1/Figure 3-source data.png]

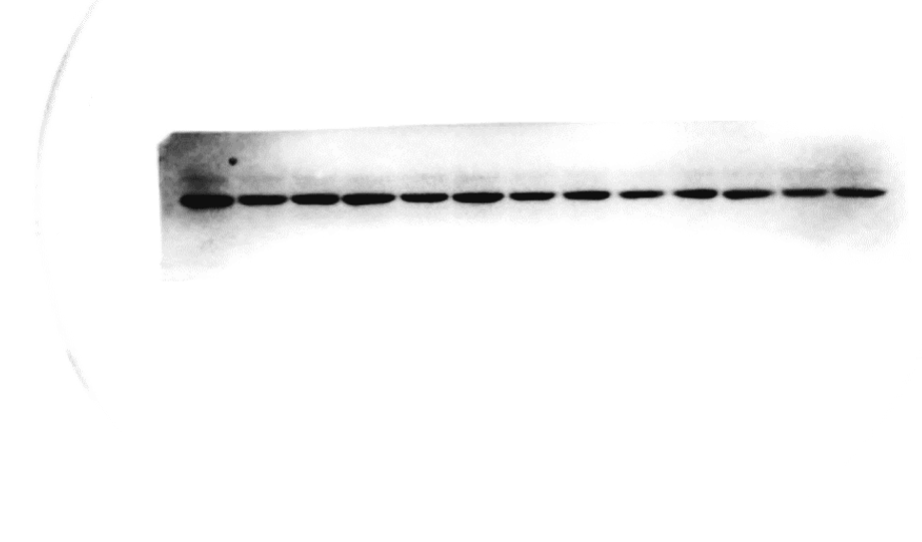

Supplement: Figure 3—source data 2. — Protein levels of phosphorylated tau (p-tau S396, p-tau T231, and p-tau S202) and tau were tested by western blot analysis in hippocampal tissue. [file elife-99462-fig3-data2.zip › Fig.3C actin 6m 9m.tif]

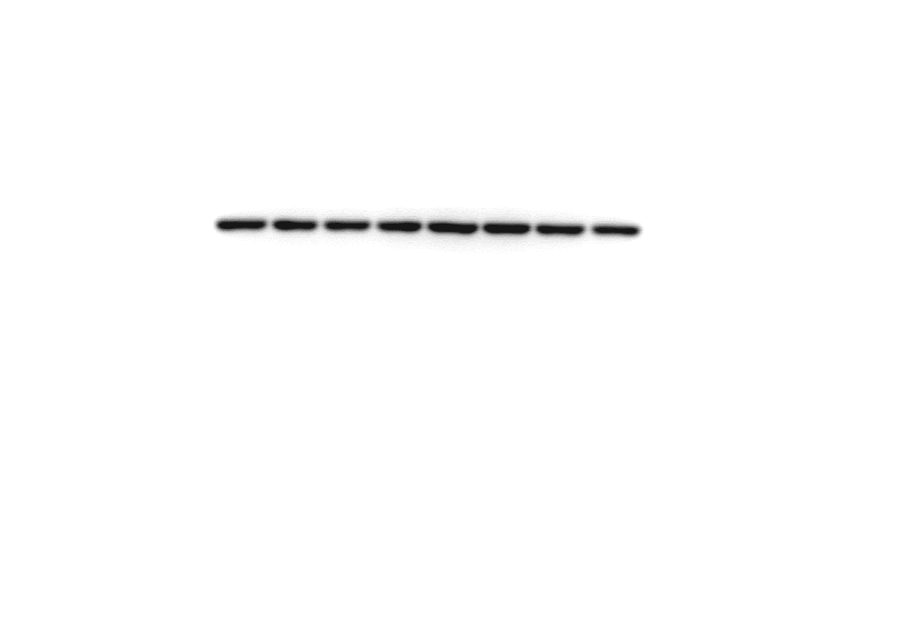

Supplement: Figure 3—source data 2. — Protein levels of phosphorylated tau (p-tau S396, p-tau T231, and p-tau S202) and tau were tested by western blot analysis in hippocampal tissue. [file elife-99462-fig3-data2.zip › Fig.3C actin S202 12m.tif]

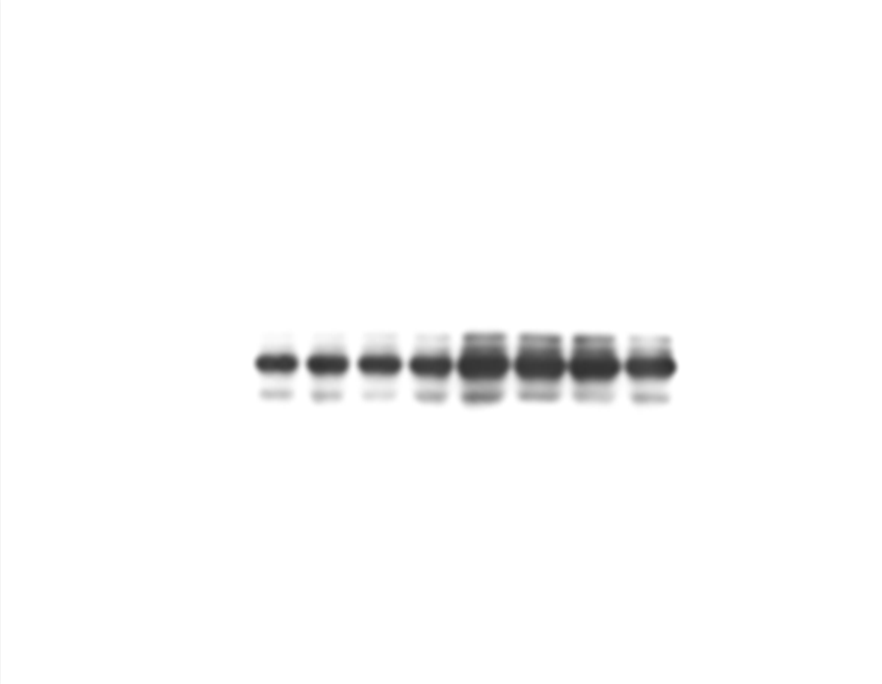

Supplement: Figure 3—source data 2. — Protein levels of phosphorylated tau (p-tau S396, p-tau T231, and p-tau S202) and tau were tested by western blot analysis in hippocampal tissue. [file elife-99462-fig3-data2.zip › Fig.3C ptau S202 12m.tif]

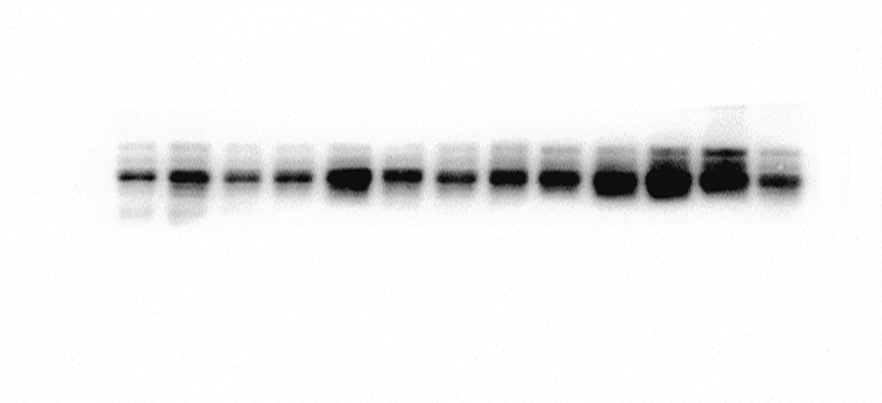

Supplement: Figure 3—source data 2. — Protein levels of phosphorylated tau (p-tau S396, p-tau T231, and p-tau S202) and tau were tested by western blot analysis in hippocampal tissue. [file elife-99462-fig3-data2.zip › Fig.3C ptau S202 6m 9m.tif]

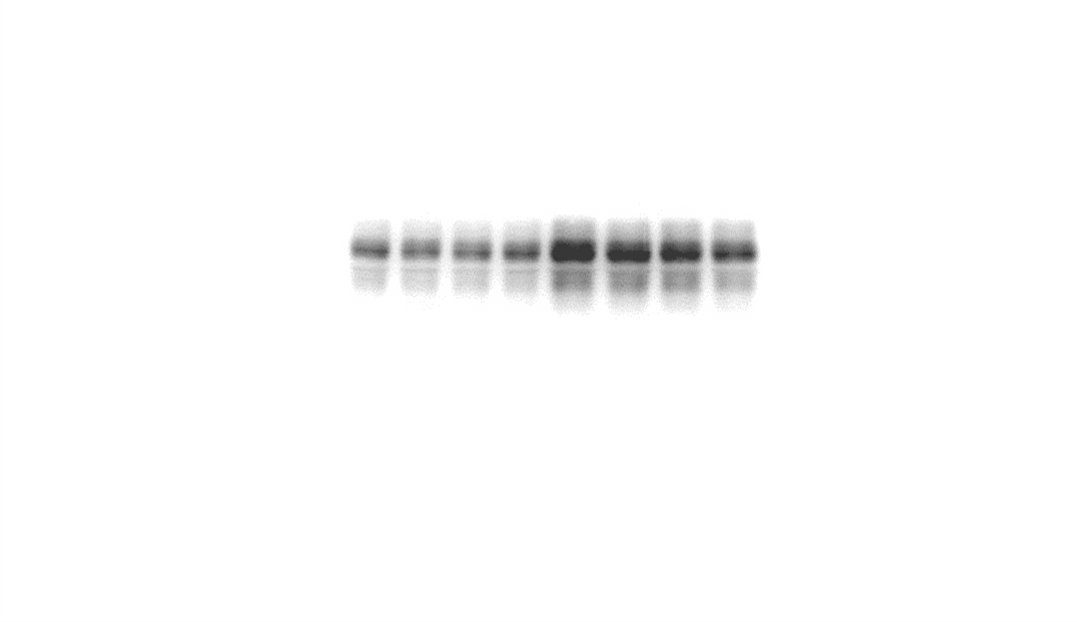

Supplement: Figure 3—source data 2. — Protein levels of phosphorylated tau (p-tau S396, p-tau T231, and p-tau S202) and tau were tested by western blot analysis in hippocampal tissue. [file elife-99462-fig3-data2.zip › Fig.3C ptau S396 12m.tif]

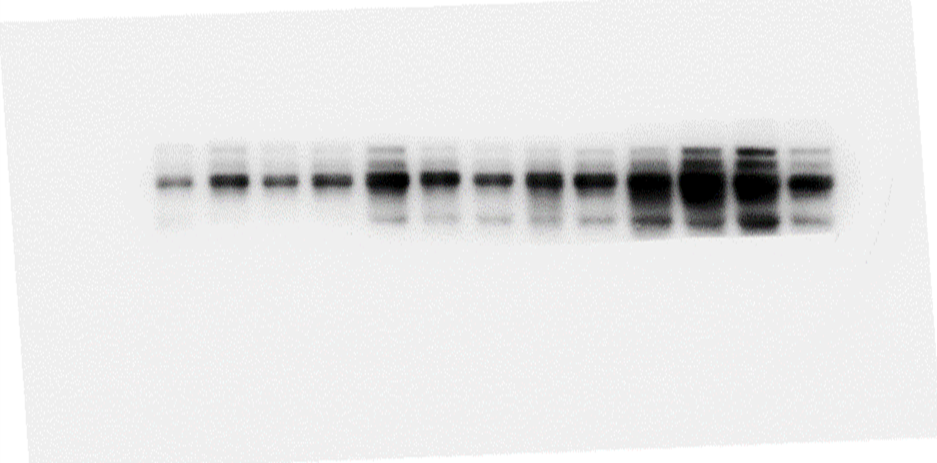

Supplement: Figure 3—source data 2. — Protein levels of phosphorylated tau (p-tau S396, p-tau T231, and p-tau S202) and tau were tested by western blot analysis in hippocampal tissue. [file elife-99462-fig3-data2.zip › Fig.3C ptau S396 6m 9m.tif]

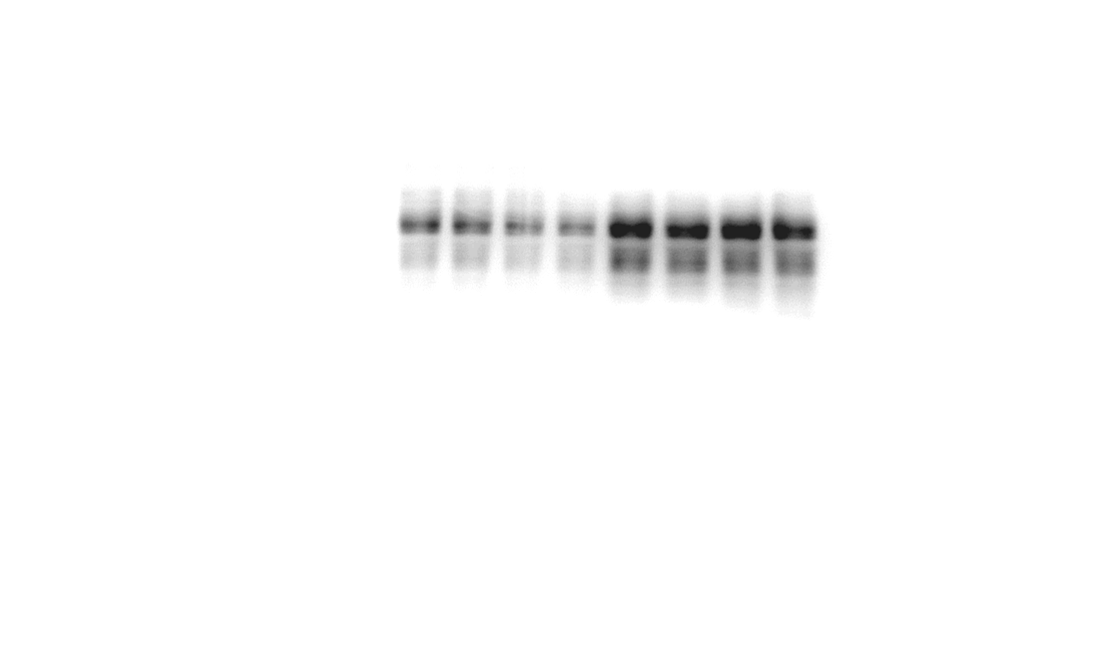

Supplement: Figure 3—source data 2. — Protein levels of phosphorylated tau (p-tau S396, p-tau T231, and p-tau S202) and tau were tested by western blot analysis in hippocampal tissue. [file elife-99462-fig3-data2.zip › Fig.3C ptau T231 12m.tif]

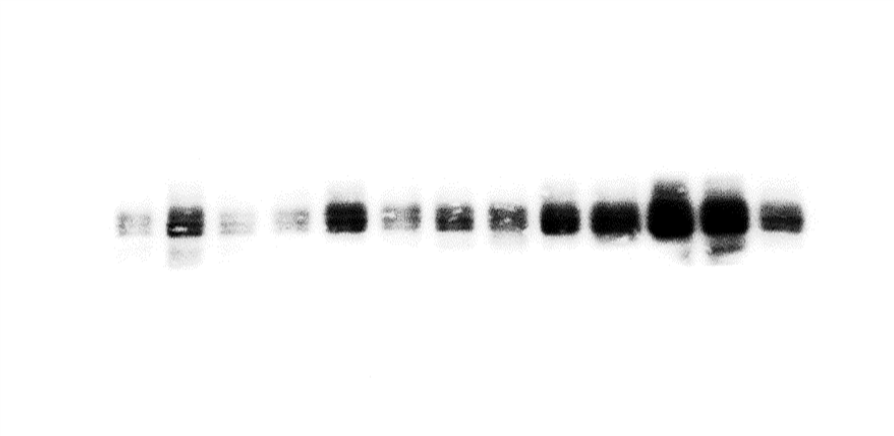

Supplement: Figure 3—source data 2. — Protein levels of phosphorylated tau (p-tau S396, p-tau T231, and p-tau S202) and tau were tested by western blot analysis in hippocampal tissue. [file elife-99462-fig3-data2.zip › Fig.3C ptau T231 6m 9m.tif]

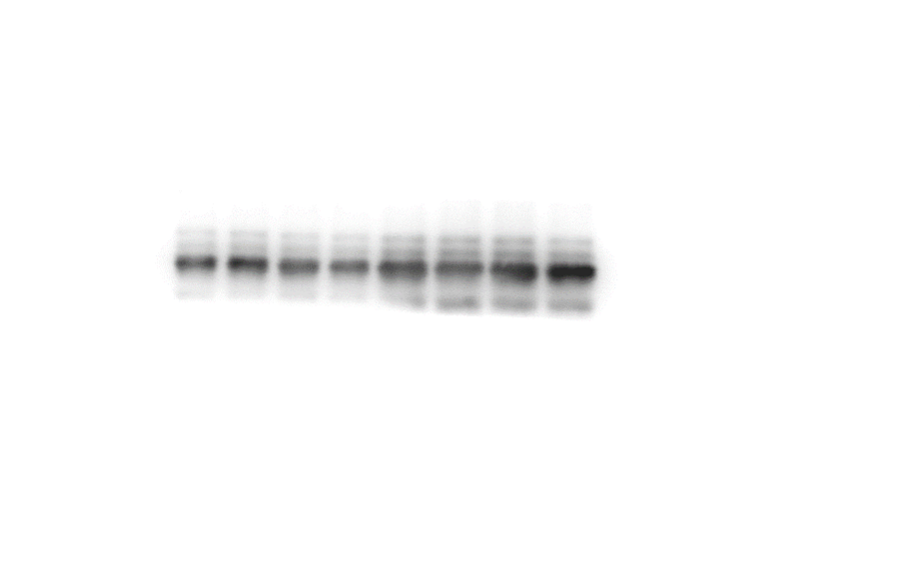

Supplement: Figure 3—source data 2. — Protein levels of phosphorylated tau (p-tau S396, p-tau T231, and p-tau S202) and tau were tested by western blot analysis in hippocampal tissue. [file elife-99462-fig3-data2.zip › Fig.3C tau 12m.tif]

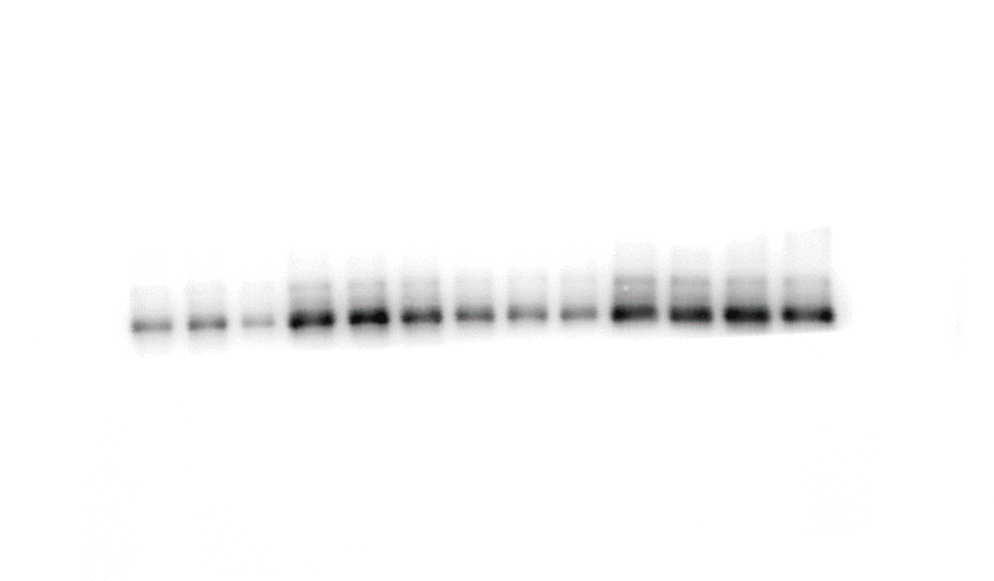

Supplement: Figure 3—source data 2. — Protein levels of phosphorylated tau (p-tau S396, p-tau T231, and p-tau S202) and tau were tested by western blot analysis in hippocampal tissue. [file elife-99462-fig3-data2.zip › Fig.3C tau 6m 9m.tif]

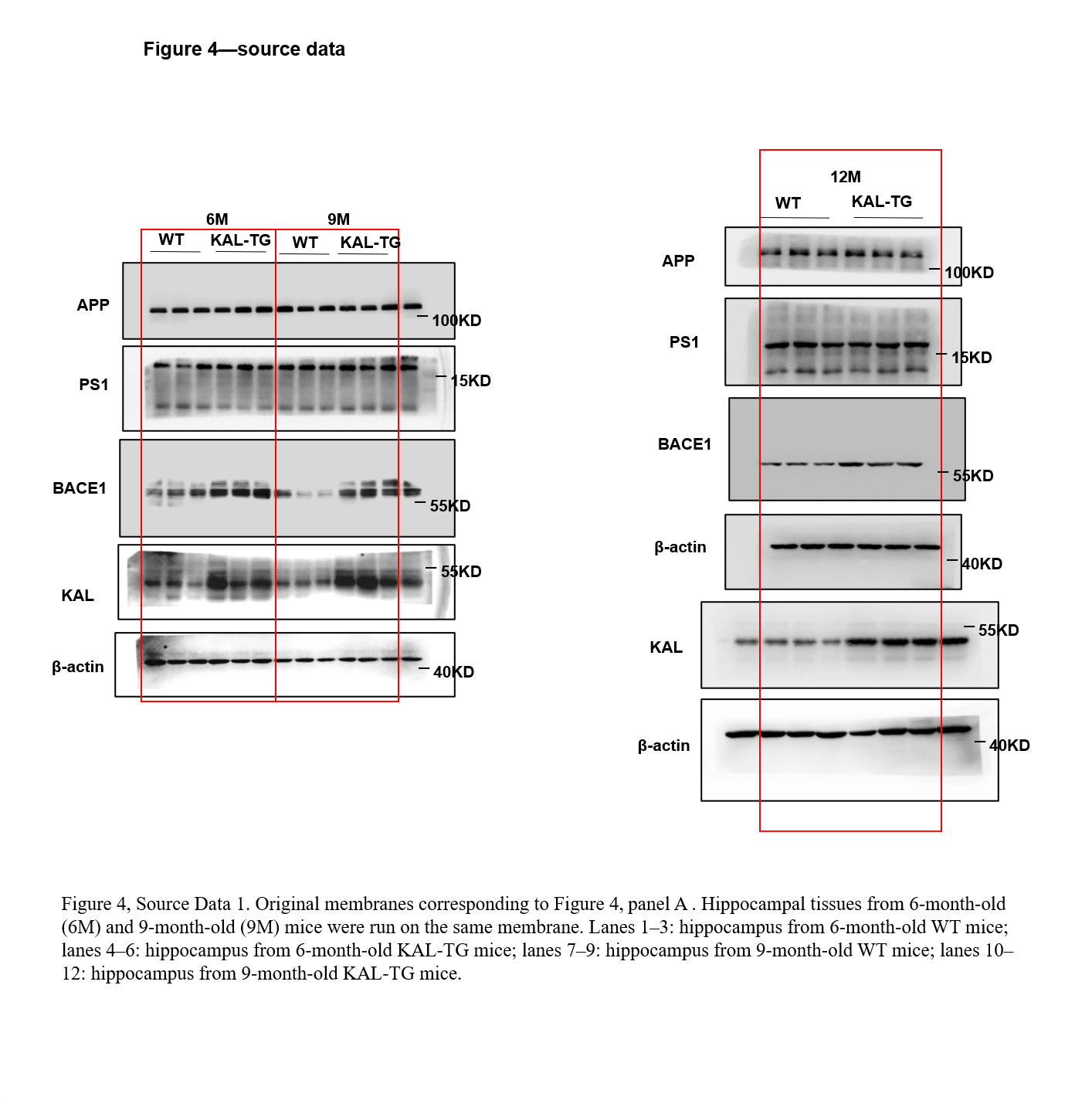

Supplement: Figure 4—source data 1. — Western blot analysis of relevant proteins, such as APP, PS1, and BACE1 during Aβ generation in hippocampal tissue of each time point (6, 9, and 12 M) KAL-TG mice and corresponding WT control groups, n = 3 per group. [file elife-99462-fig4-data1.zip › Figure 4-source data 1/Figure 4-source data.png]

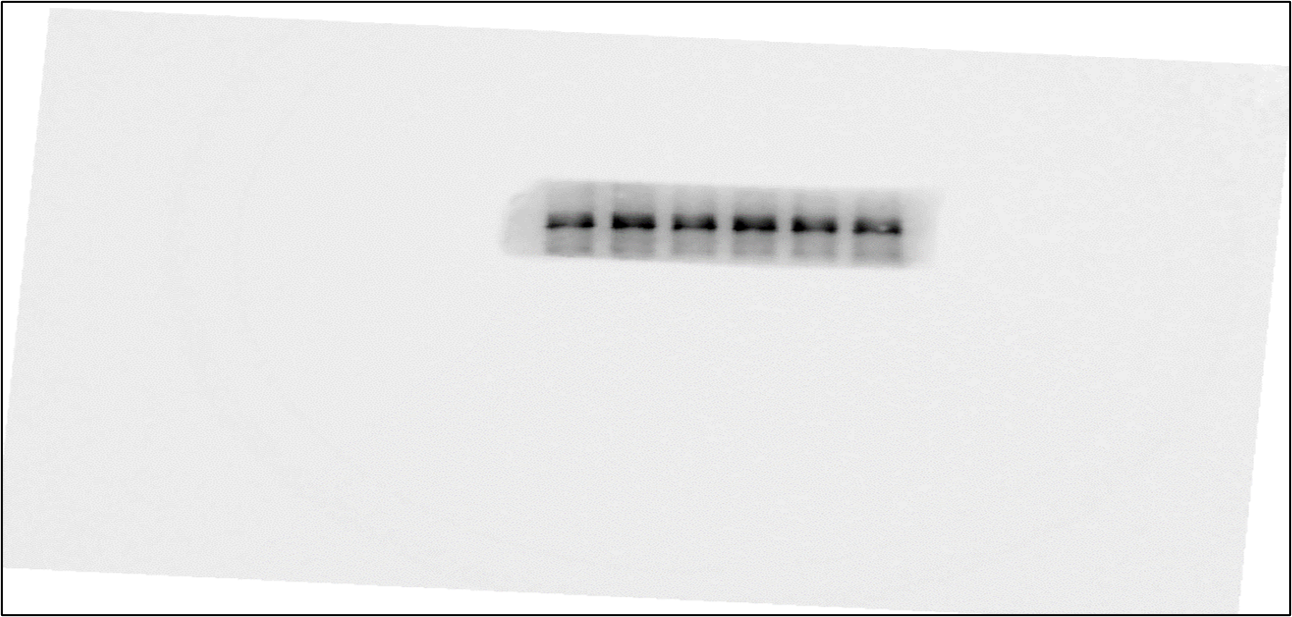

Supplement: Figure 4—source data 2. — Western blot analysis of relevant proteins, such as APP, PS1, and BACE1 during Aβ generation in hippocampal tissue of each time point (6, 9, and 12 M) KAL-TG mice and corresponding WT control groups, n = 3 per group. [file elife-99462-fig4-data2.zip › Fig.4A APP 12m.tif]

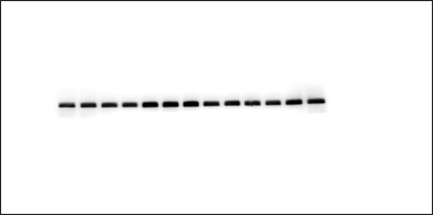

Supplement: Figure 4—source data 2. — Western blot analysis of relevant proteins, such as APP, PS1, and BACE1 during Aβ generation in hippocampal tissue of each time point (6, 9, and 12 M) KAL-TG mice and corresponding WT control groups, n = 3 per group. [file elife-99462-fig4-data2.zip › Fig.4A APP 6m 9m.tif]

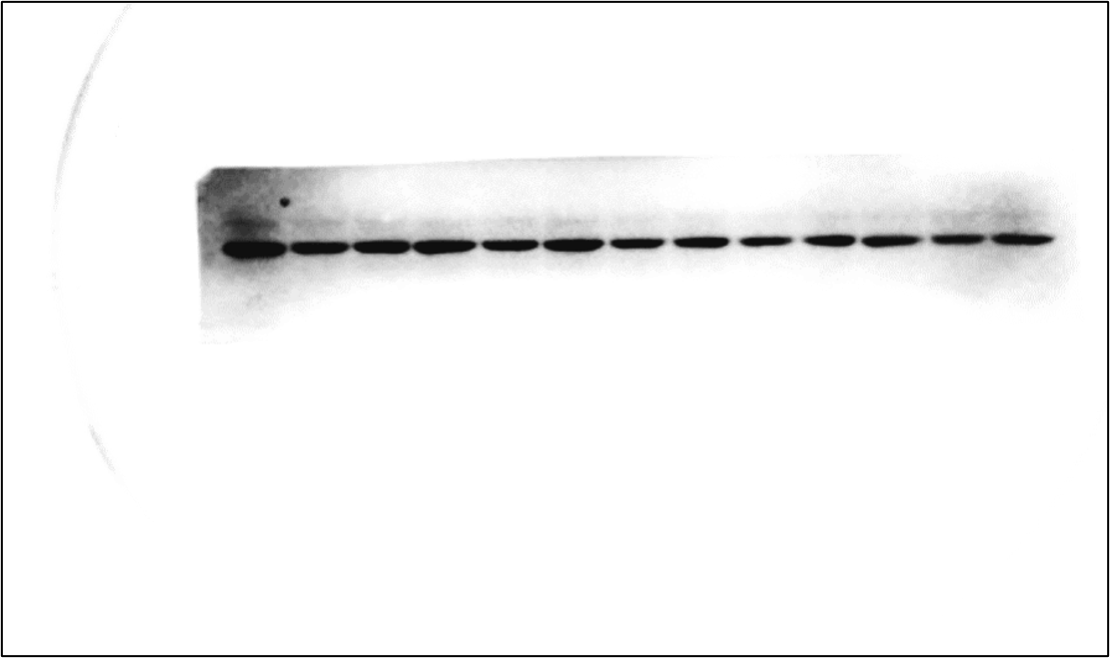

Supplement: Figure 4—source data 2. — Western blot analysis of relevant proteins, such as APP, PS1, and BACE1 during Aβ generation in hippocampal tissue of each time point (6, 9, and 12 M) KAL-TG mice and corresponding WT control groups, n = 3 per group. [file elife-99462-fig4-data2.zip › Fig.4A Actin 6m 9m.tif]

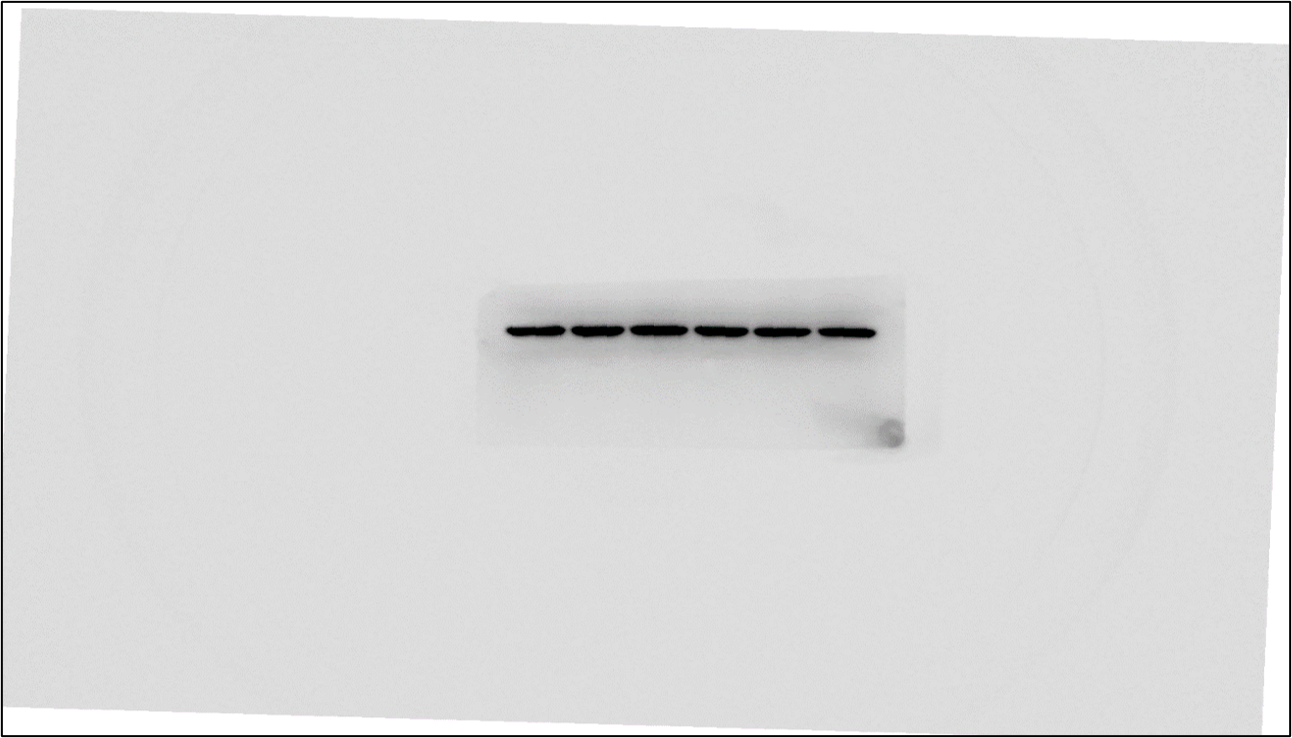

Supplement: Figure 4—source data 2. — Western blot analysis of relevant proteins, such as APP, PS1, and BACE1 during Aβ generation in hippocampal tissue of each time point (6, 9, and 12 M) KAL-TG mice and corresponding WT control groups, n = 3 per group. [file elife-99462-fig4-data2.zip › Fig.4A Actin1 12m.tif]

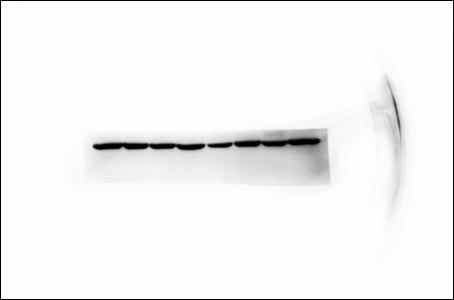

Supplement: Figure 4—source data 2. — Western blot analysis of relevant proteins, such as APP, PS1, and BACE1 during Aβ generation in hippocampal tissue of each time point (6, 9, and 12 M) KAL-TG mice and corresponding WT control groups, n = 3 per group. [file elife-99462-fig4-data2.zip › Fig.4A Actin2 12m.tif]

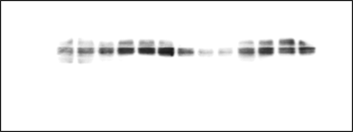

Supplement: Figure 4—source data 2. — Western blot analysis of relevant proteins, such as APP, PS1, and BACE1 during Aβ generation in hippocampal tissue of each time point (6, 9, and 12 M) KAL-TG mice and corresponding WT control groups, n = 3 per group. [file elife-99462-fig4-data2.zip › Fig.4A BACE 6m 9m.tif]

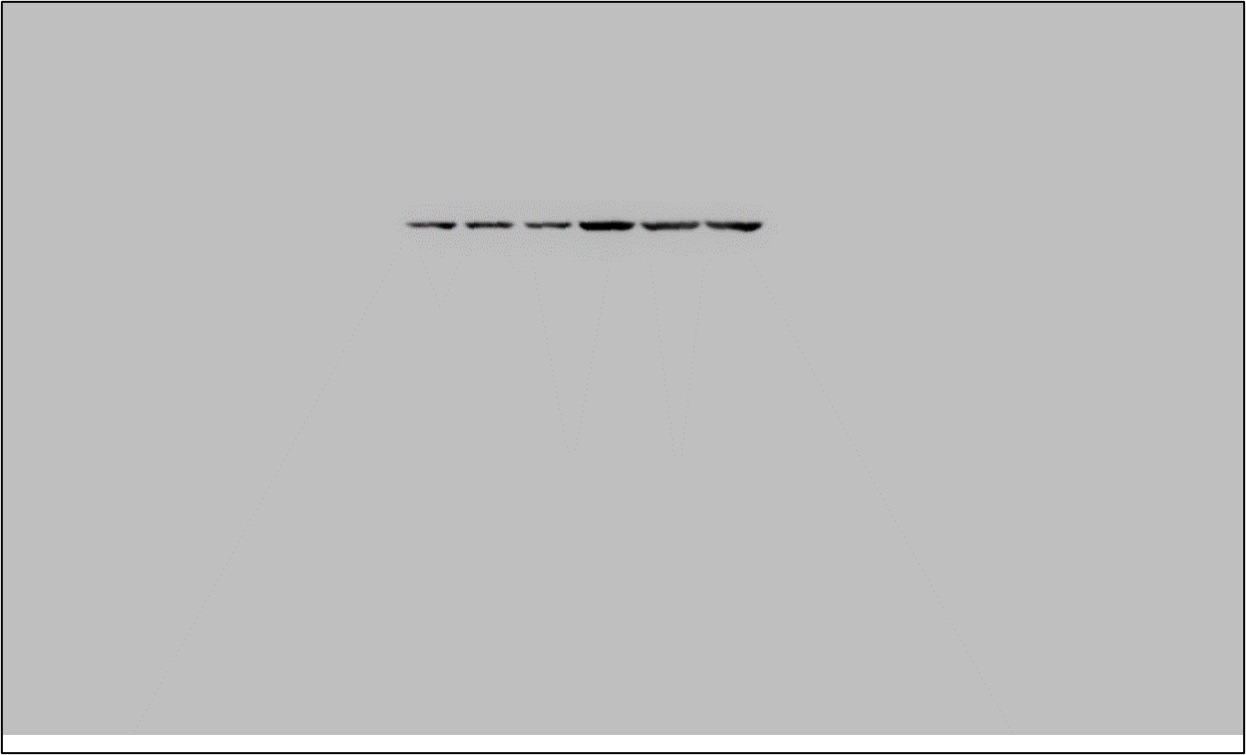

Supplement: Figure 4—source data 2. — Western blot analysis of relevant proteins, such as APP, PS1, and BACE1 during Aβ generation in hippocampal tissue of each time point (6, 9, and 12 M) KAL-TG mice and corresponding WT control groups, n = 3 per group. [file elife-99462-fig4-data2.zip › Fig.4A BACE12m.tif]

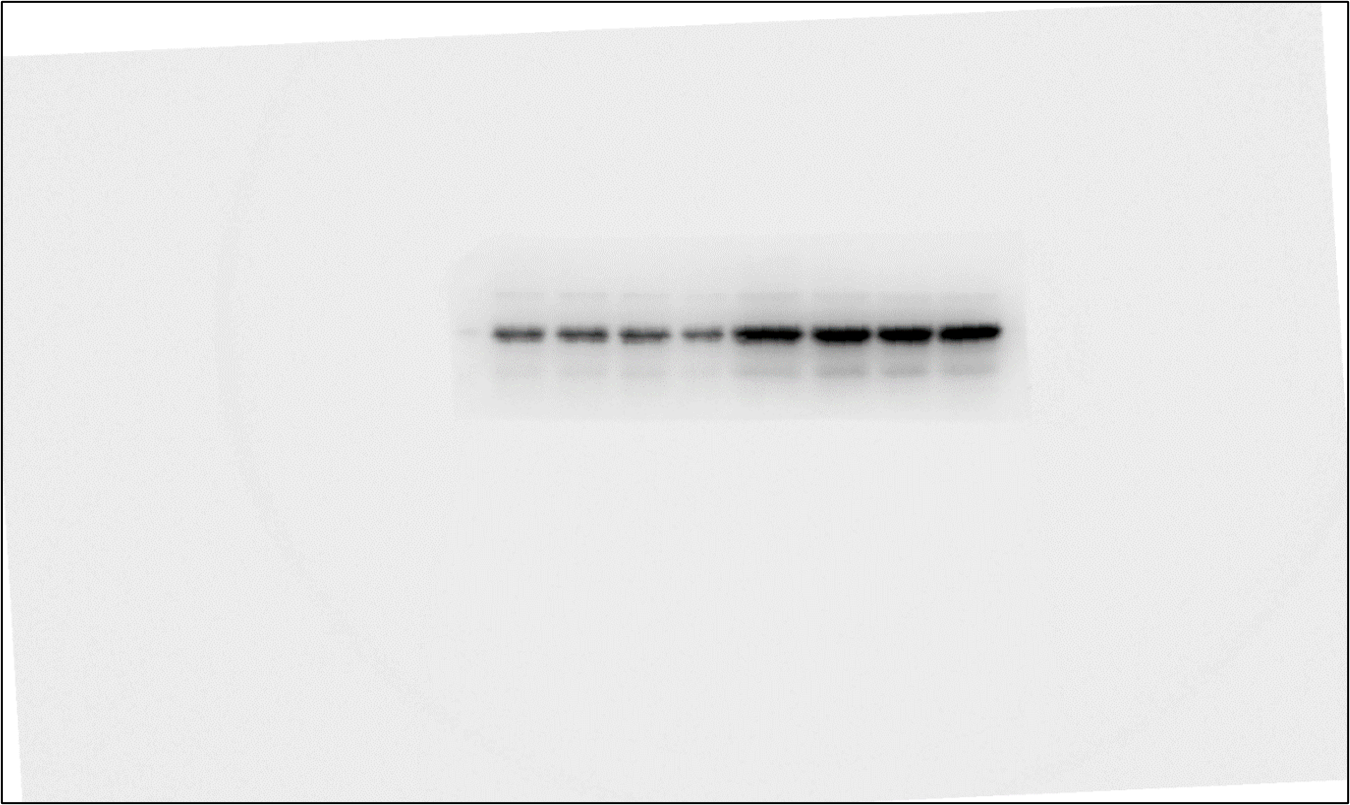

Supplement: Figure 4—source data 2. — Western blot analysis of relevant proteins, such as APP, PS1, and BACE1 during Aβ generation in hippocampal tissue of each time point (6, 9, and 12 M) KAL-TG mice and corresponding WT control groups, n = 3 per group. [file elife-99462-fig4-data2.zip › Fig.4A KAL 12m.tif]

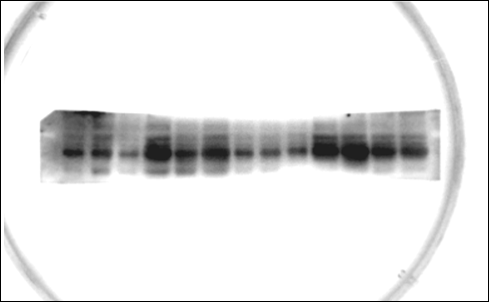

Supplement: Figure 4—source data 2. — Western blot analysis of relevant proteins, such as APP, PS1, and BACE1 during Aβ generation in hippocampal tissue of each time point (6, 9, and 12 M) KAL-TG mice and corresponding WT control groups, n = 3 per group. [file elife-99462-fig4-data2.zip › Fig.4A KAL 6m 9m.tif]

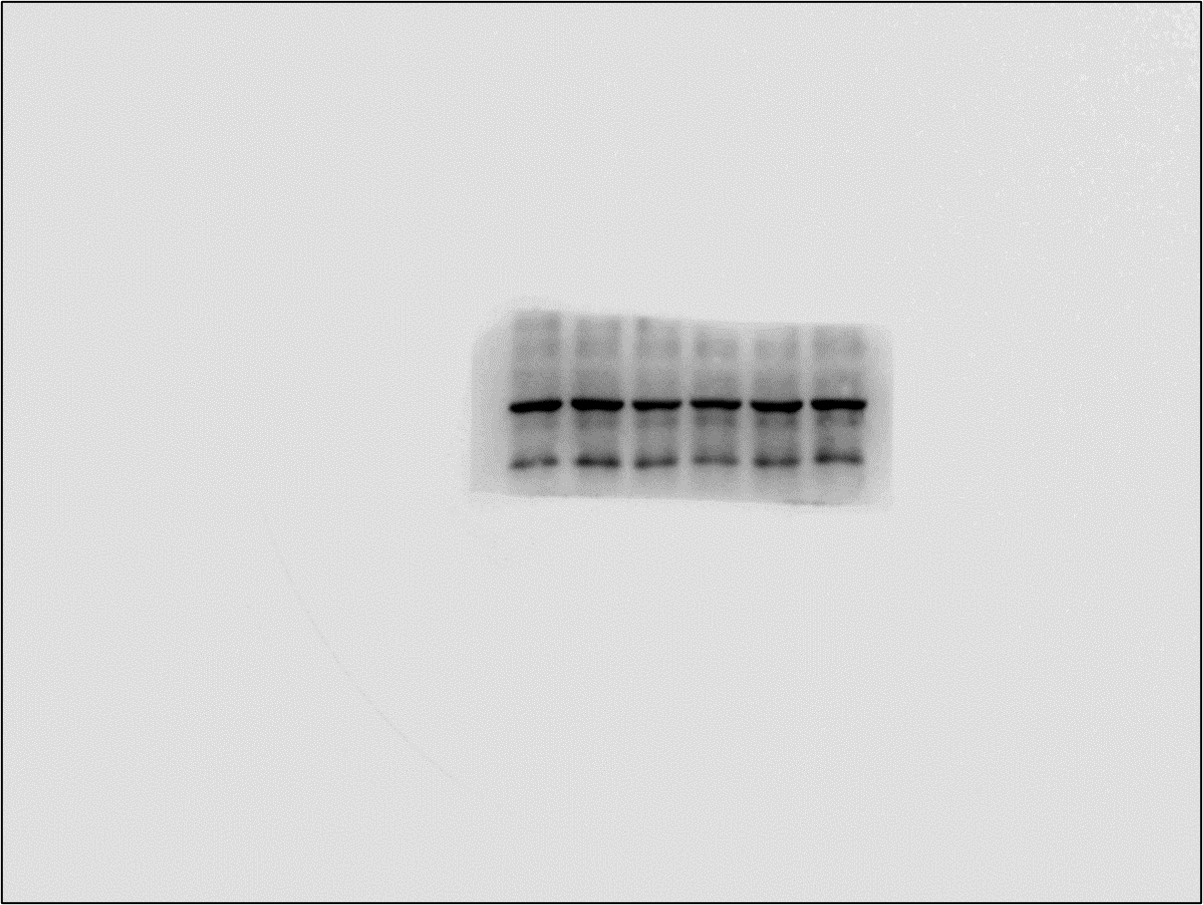

Supplement: Figure 4—source data 2. — Western blot analysis of relevant proteins, such as APP, PS1, and BACE1 during Aβ generation in hippocampal tissue of each time point (6, 9, and 12 M) KAL-TG mice and corresponding WT control groups, n = 3 per group. [file elife-99462-fig4-data2.zip › Fig.4A PS1 12m.tif]

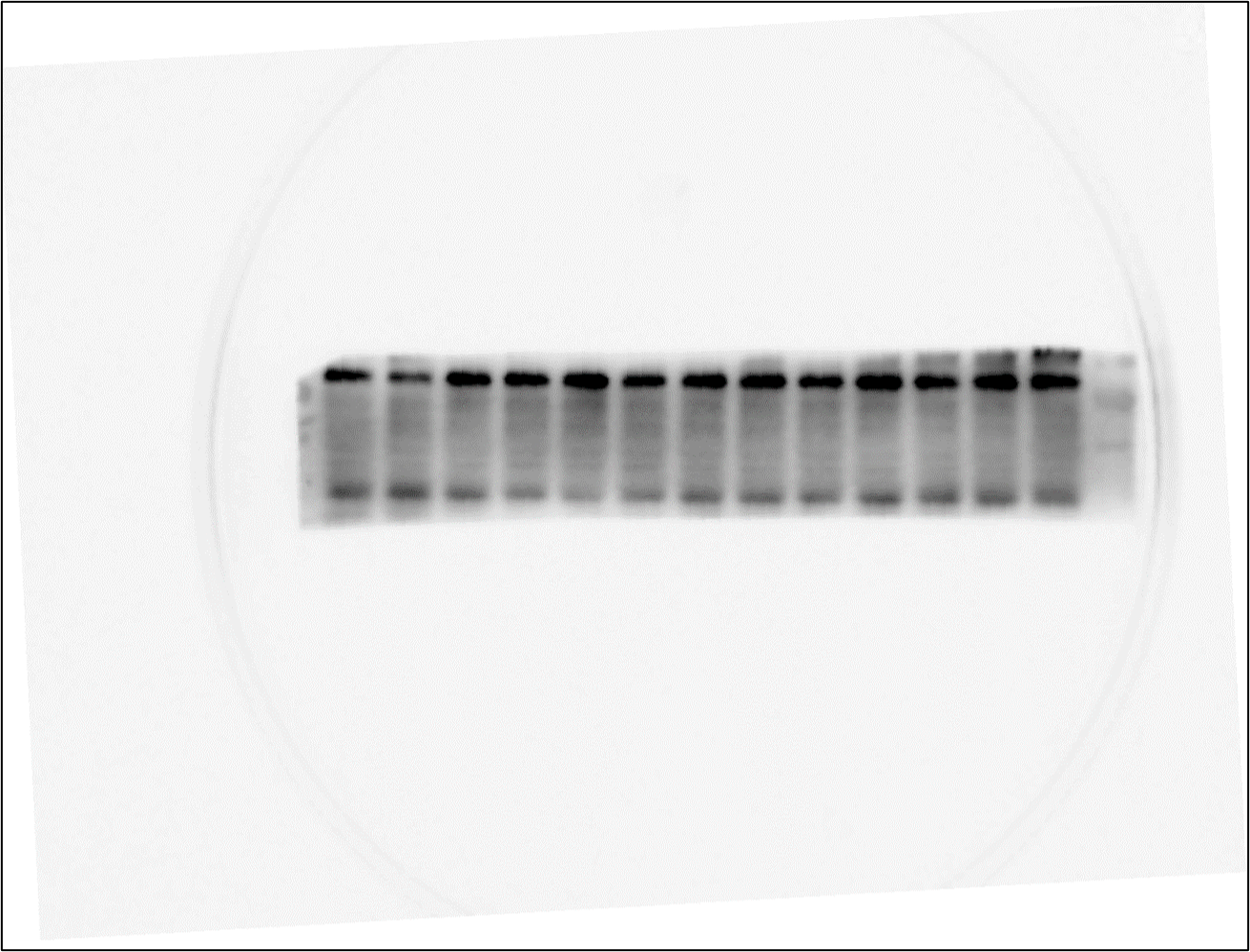

Supplement: Figure 4—source data 2. — Western blot analysis of relevant proteins, such as APP, PS1, and BACE1 during Aβ generation in hippocampal tissue of each time point (6, 9, and 12 M) KAL-TG mice and corresponding WT control groups, n = 3 per group. [file elife-99462-fig4-data2.zip › Fig.4A PS1 6m 9m.tif]

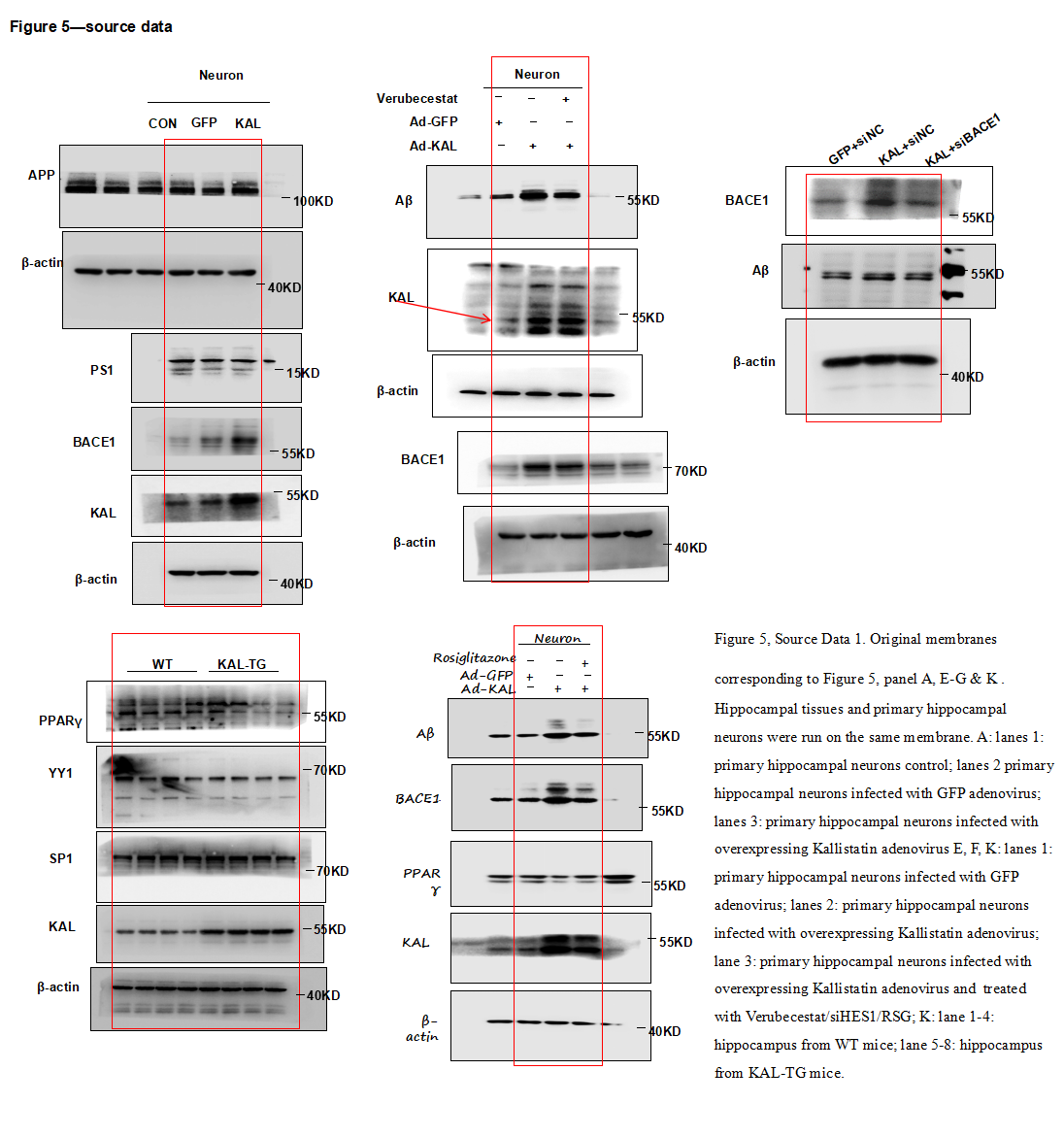

Supplement: Figure 5—source data 1. — Hippocampal tissues and primary hippocampal neurons were run on the same membrane. A: lanes 1: primary hippocampal neurons control; lanes 2: primary hippocampal neurons infected with GFP adenovirus; lanes 3: primary hippocampal neurons infected with overexpressing Kallistatin adenovirus. E, F, G: lanes 1: primary hippocampal neurons infected with GFP adenovirus; lanes 2: primary hippocampal neurons infected with overexpressing Kallistatin adenovirus; lanes 3: primary hippocampal neurons infected with overexpressing Kallistatin adenovirus and treated with Verubecestat/siHESl/RSG; K: lanes 1-4: hippocampus from WT mice; lanes 5-8: hippocampusfom KAL-TG mice. [file elife-99462-fig5-data1.zip › Figure 5-source data 1/Figure 5-source data.png]

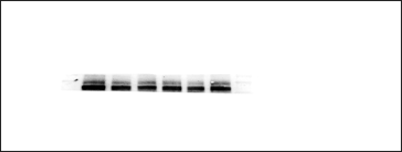

Supplement: Figure 5—source data 2. — Hippocampal tissues and primary hippocampal neurons were run on the same membrane. A: lanes 1: primary hippocampal neurons control; lanes 2: primary hippocampal neurons infected with GFP adenovirus; lanes 3: primary hippocampal neurons infected with overexpressing Kallistatin adenovirus. E, F, G: lanes 1: primary hippocampal neurons infected with GFP adenovirus; lanes 2: primary hippocampal neurons infected with overexpressing Kallistatin adenovirus; lanes 3: primary hippocampal neurons infected with overexpressing Kallistatin adenovirus and treated with Verubecestat/siHESl/RSG; K: lanes 1-4: hippocampus from WT mice; lanes 5-8: hippocampusfom KAL-TG mice. [file elife-99462-fig5-data2.zip › Fig5A APP Neuron.tif]

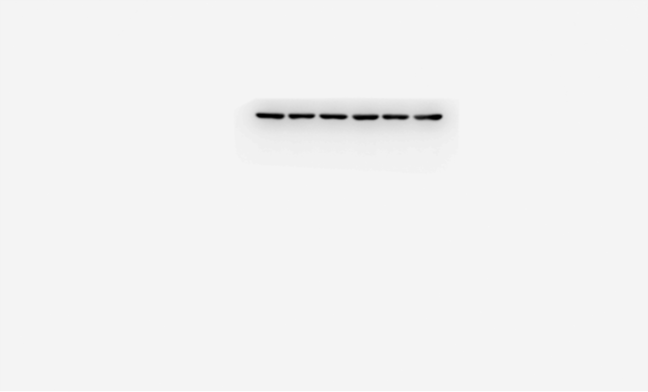

Supplement: Figure 5—source data 2. — Hippocampal tissues and primary hippocampal neurons were run on the same membrane. A: lanes 1: primary hippocampal neurons control; lanes 2: primary hippocampal neurons infected with GFP adenovirus; lanes 3: primary hippocampal neurons infected with overexpressing Kallistatin adenovirus. E, F, G: lanes 1: primary hippocampal neurons infected with GFP adenovirus; lanes 2: primary hippocampal neurons infected with overexpressing Kallistatin adenovirus; lanes 3: primary hippocampal neurons infected with overexpressing Kallistatin adenovirus and treated with Verubecestat/siHESl/RSG; K: lanes 1-4: hippocampus from WT mice; lanes 5-8: hippocampusfom KAL-TG mice. [file elife-99462-fig5-data2.zip › Fig5A Actin1 Neuron.tif]

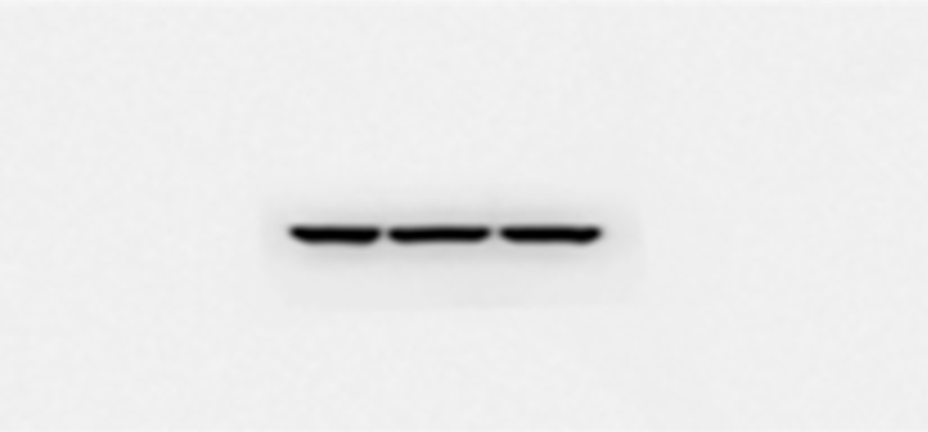

Supplement: Figure 5—source data 2. — Hippocampal tissues and primary hippocampal neurons were run on the same membrane. A: lanes 1: primary hippocampal neurons control; lanes 2: primary hippocampal neurons infected with GFP adenovirus; lanes 3: primary hippocampal neurons infected with overexpressing Kallistatin adenovirus. E, F, G: lanes 1: primary hippocampal neurons infected with GFP adenovirus; lanes 2: primary hippocampal neurons infected with overexpressing Kallistatin adenovirus; lanes 3: primary hippocampal neurons infected with overexpressing Kallistatin adenovirus and treated with Verubecestat/siHESl/RSG; K: lanes 1-4: hippocampus from WT mice; lanes 5-8: hippocampusfom KAL-TG mice. [file elife-99462-fig5-data2.zip › Fig5A Actin2 Neuron.tif]

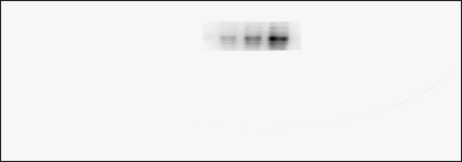

Supplement: Figure 5—source data 2. — Hippocampal tissues and primary hippocampal neurons were run on the same membrane. A: lanes 1: primary hippocampal neurons control; lanes 2: primary hippocampal neurons infected with GFP adenovirus; lanes 3: primary hippocampal neurons infected with overexpressing Kallistatin adenovirus. E, F, G: lanes 1: primary hippocampal neurons infected with GFP adenovirus; lanes 2: primary hippocampal neurons infected with overexpressing Kallistatin adenovirus; lanes 3: primary hippocampal neurons infected with overexpressing Kallistatin adenovirus and treated with Verubecestat/siHESl/RSG; K: lanes 1-4: hippocampus from WT mice; lanes 5-8: hippocampusfom KAL-TG mice. [file elife-99462-fig5-data2.zip › Fig5A BACE1 Neuron.tif]

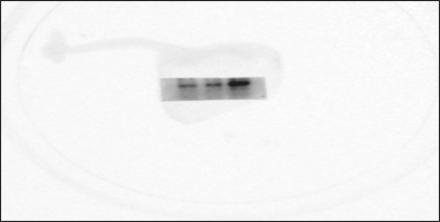

Supplement: Figure 5—source data 2. — Hippocampal tissues and primary hippocampal neurons were run on the same membrane. A: lanes 1: primary hippocampal neurons control; lanes 2: primary hippocampal neurons infected with GFP adenovirus; lanes 3: primary hippocampal neurons infected with overexpressing Kallistatin adenovirus. E, F, G: lanes 1: primary hippocampal neurons infected with GFP adenovirus; lanes 2: primary hippocampal neurons infected with overexpressing Kallistatin adenovirus; lanes 3: primary hippocampal neurons infected with overexpressing Kallistatin adenovirus and treated with Verubecestat/siHESl/RSG; K: lanes 1-4: hippocampus from WT mice; lanes 5-8: hippocampusfom KAL-TG mice. [file elife-99462-fig5-data2.zip › Fig5A KAL Neuron.tif]

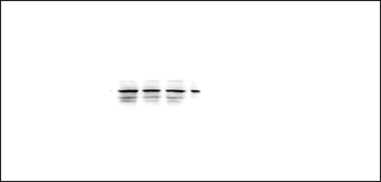

Supplement: Figure 5—source data 2. — Hippocampal tissues and primary hippocampal neurons were run on the same membrane. A: lanes 1: primary hippocampal neurons control; lanes 2: primary hippocampal neurons infected with GFP adenovirus; lanes 3: primary hippocampal neurons infected with overexpressing Kallistatin adenovirus. E, F, G: lanes 1: primary hippocampal neurons infected with GFP adenovirus; lanes 2: primary hippocampal neurons infected with overexpressing Kallistatin adenovirus; lanes 3: primary hippocampal neurons infected with overexpressing Kallistatin adenovirus and treated with Verubecestat/siHESl/RSG; K: lanes 1-4: hippocampus from WT mice; lanes 5-8: hippocampusfom KAL-TG mice. [file elife-99462-fig5-data2.zip › Fig5A PS1 Neuron.tif]

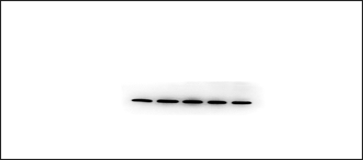

Supplement: Figure 5—source data 2. — Hippocampal tissues and primary hippocampal neurons were run on the same membrane. A: lanes 1: primary hippocampal neurons control; lanes 2: primary hippocampal neurons infected with GFP adenovirus; lanes 3: primary hippocampal neurons infected with overexpressing Kallistatin adenovirus. E, F, G: lanes 1: primary hippocampal neurons infected with GFP adenovirus; lanes 2: primary hippocampal neurons infected with overexpressing Kallistatin adenovirus; lanes 3: primary hippocampal neurons infected with overexpressing Kallistatin adenovirus and treated with Verubecestat/siHESl/RSG; K: lanes 1-4: hippocampus from WT mice; lanes 5-8: hippocampusfom KAL-TG mice. [file elife-99462-fig5-data2.zip › Fig5E Actin1 Neuron.tif]

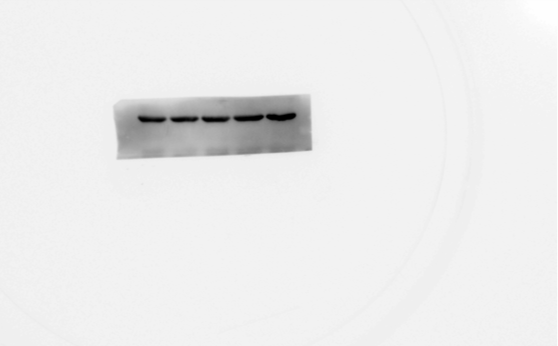

Supplement: Figure 5—source data 2. — Hippocampal tissues and primary hippocampal neurons were run on the same membrane. A: lanes 1: primary hippocampal neurons control; lanes 2: primary hippocampal neurons infected with GFP adenovirus; lanes 3: primary hippocampal neurons infected with overexpressing Kallistatin adenovirus. E, F, G: lanes 1: primary hippocampal neurons infected with GFP adenovirus; lanes 2: primary hippocampal neurons infected with overexpressing Kallistatin adenovirus; lanes 3: primary hippocampal neurons infected with overexpressing Kallistatin adenovirus and treated with Verubecestat/siHESl/RSG; K: lanes 1-4: hippocampus from WT mice; lanes 5-8: hippocampusfom KAL-TG mice. [file elife-99462-fig5-data2.zip › Fig5E Actin2 Neuron.tif]

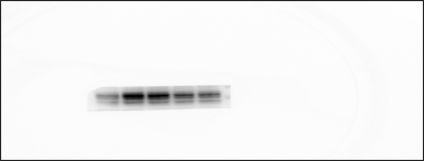

Supplement: Figure 5—source data 2. — Hippocampal tissues and primary hippocampal neurons were run on the same membrane. A: lanes 1: primary hippocampal neurons control; lanes 2: primary hippocampal neurons infected with GFP adenovirus; lanes 3: primary hippocampal neurons infected with overexpressing Kallistatin adenovirus. E, F, G: lanes 1: primary hippocampal neurons infected with GFP adenovirus; lanes 2: primary hippocampal neurons infected with overexpressing Kallistatin adenovirus; lanes 3: primary hippocampal neurons infected with overexpressing Kallistatin adenovirus and treated with Verubecestat/siHESl/RSG; K: lanes 1-4: hippocampus from WT mice; lanes 5-8: hippocampusfom KAL-TG mice. [file elife-99462-fig5-data2.zip › Fig5E BACE1 Neuron.tif]

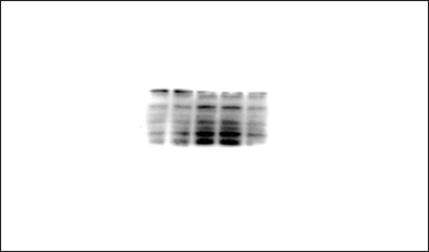

Supplement: Figure 5—source data 2. — Hippocampal tissues and primary hippocampal neurons were run on the same membrane. A: lanes 1: primary hippocampal neurons control; lanes 2: primary hippocampal neurons infected with GFP adenovirus; lanes 3: primary hippocampal neurons infected with overexpressing Kallistatin adenovirus. E, F, G: lanes 1: primary hippocampal neurons infected with GFP adenovirus; lanes 2: primary hippocampal neurons infected with overexpressing Kallistatin adenovirus; lanes 3: primary hippocampal neurons infected with overexpressing Kallistatin adenovirus and treated with Verubecestat/siHESl/RSG; K: lanes 1-4: hippocampus from WT mice; lanes 5-8: hippocampusfom KAL-TG mice. [file elife-99462-fig5-data2.zip › Fig5E KAL Neuron.tif]

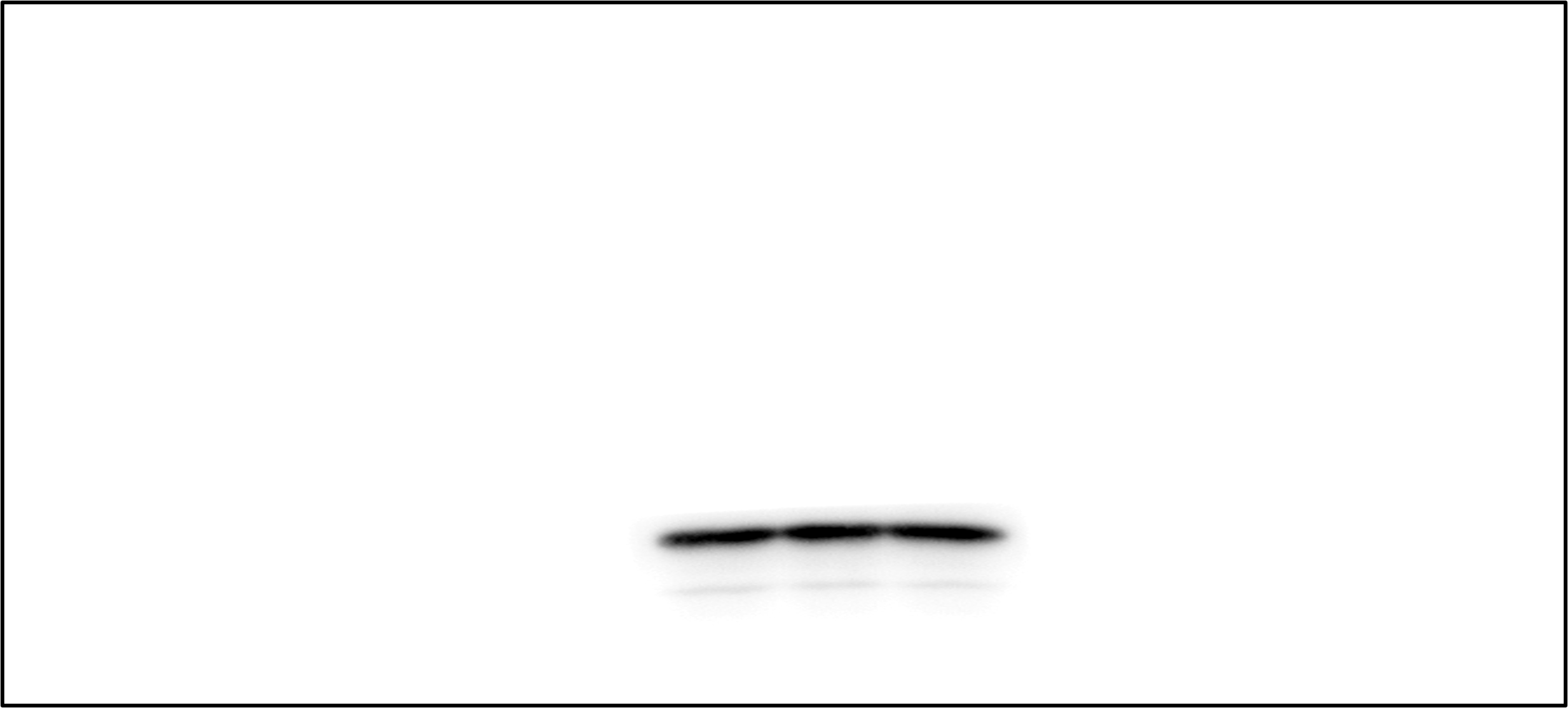

Supplement: Figure 5—source data 2. — Hippocampal tissues and primary hippocampal neurons were run on the same membrane. A: lanes 1: primary hippocampal neurons control; lanes 2: primary hippocampal neurons infected with GFP adenovirus; lanes 3: primary hippocampal neurons infected with overexpressing Kallistatin adenovirus. E, F, G: lanes 1: primary hippocampal neurons infected with GFP adenovirus; lanes 2: primary hippocampal neurons infected with overexpressing Kallistatin adenovirus; lanes 3: primary hippocampal neurons infected with overexpressing Kallistatin adenovirus and treated with Verubecestat/siHESl/RSG; K: lanes 1-4: hippocampus from WT mice; lanes 5-8: hippocampusfom KAL-TG mice. [file elife-99462-fig5-data2.zip › Fig5F Actin Neuron.tif]

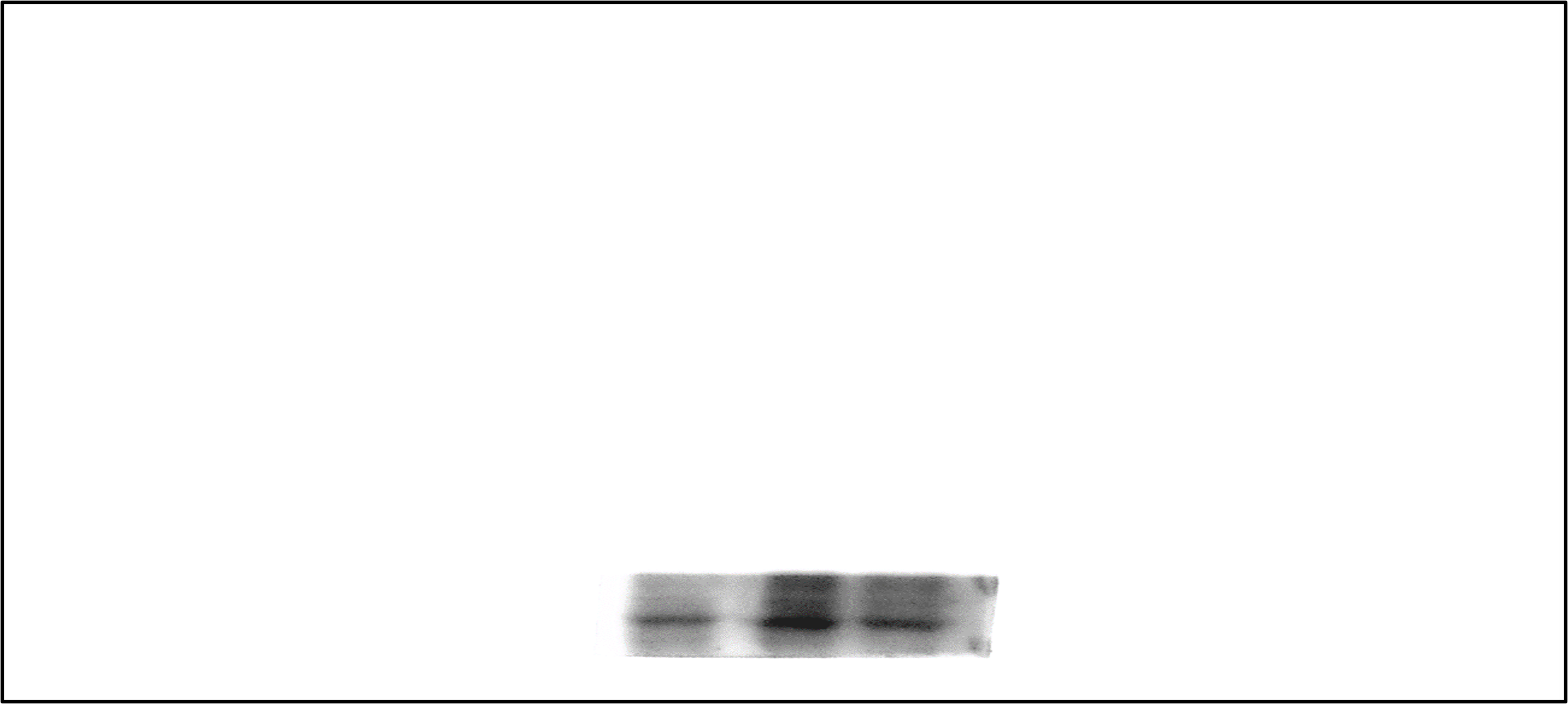

Supplement: Figure 5—source data 2. — Hippocampal tissues and primary hippocampal neurons were run on the same membrane. A: lanes 1: primary hippocampal neurons control; lanes 2: primary hippocampal neurons infected with GFP adenovirus; lanes 3: primary hippocampal neurons infected with overexpressing Kallistatin adenovirus. E, F, G: lanes 1: primary hippocampal neurons infected with GFP adenovirus; lanes 2: primary hippocampal neurons infected with overexpressing Kallistatin adenovirus; lanes 3: primary hippocampal neurons infected with overexpressing Kallistatin adenovirus and treated with Verubecestat/siHESl/RSG; K: lanes 1-4: hippocampus from WT mice; lanes 5-8: hippocampusfom KAL-TG mice. [file elife-99462-fig5-data2.zip › Fig5F BACE1 Neuron.tif]

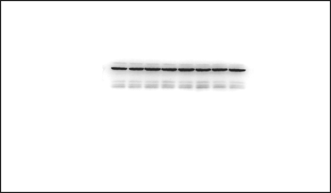

Supplement: Figure 5—source data 2. — Hippocampal tissues and primary hippocampal neurons were run on the same membrane. A: lanes 1: primary hippocampal neurons control; lanes 2: primary hippocampal neurons infected with GFP adenovirus; lanes 3: primary hippocampal neurons infected with overexpressing Kallistatin adenovirus. E, F, G: lanes 1: primary hippocampal neurons infected with GFP adenovirus; lanes 2: primary hippocampal neurons infected with overexpressing Kallistatin adenovirus; lanes 3: primary hippocampal neurons infected with overexpressing Kallistatin adenovirus and treated with Verubecestat/siHESl/RSG; K: lanes 1-4: hippocampus from WT mice; lanes 5-8: hippocampusfom KAL-TG mice. [file elife-99462-fig5-data2.zip › Fig5G Actin hippocampal tissue.tif]

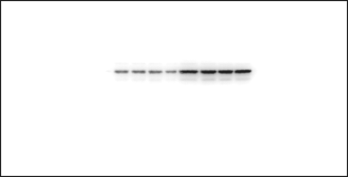

Supplement: Figure 5—source data 2. — Hippocampal tissues and primary hippocampal neurons were run on the same membrane. A: lanes 1: primary hippocampal neurons control; lanes 2: primary hippocampal neurons infected with GFP adenovirus; lanes 3: primary hippocampal neurons infected with overexpressing Kallistatin adenovirus. E, F, G: lanes 1: primary hippocampal neurons infected with GFP adenovirus; lanes 2: primary hippocampal neurons infected with overexpressing Kallistatin adenovirus; lanes 3: primary hippocampal neurons infected with overexpressing Kallistatin adenovirus and treated with Verubecestat/siHESl/RSG; K: lanes 1-4: hippocampus from WT mice; lanes 5-8: hippocampusfom KAL-TG mice. [file elife-99462-fig5-data2.zip › Fig5G KAL hippocampal tissue.tif]

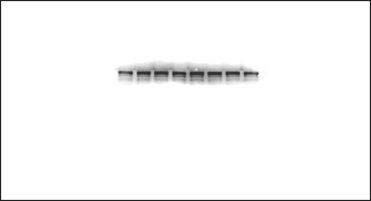

Supplement: Figure 5—source data 2. — Hippocampal tissues and primary hippocampal neurons were run on the same membrane. A: lanes 1: primary hippocampal neurons control; lanes 2: primary hippocampal neurons infected with GFP adenovirus; lanes 3: primary hippocampal neurons infected with overexpressing Kallistatin adenovirus. E, F, G: lanes 1: primary hippocampal neurons infected with GFP adenovirus; lanes 2: primary hippocampal neurons infected with overexpressing Kallistatin adenovirus; lanes 3: primary hippocampal neurons infected with overexpressing Kallistatin adenovirus and treated with Verubecestat/siHESl/RSG; K: lanes 1-4: hippocampus from WT mice; lanes 5-8: hippocampusfom KAL-TG mice. [file elife-99462-fig5-data2.zip › Fig5G SP1 hippocampal tissue.tif]

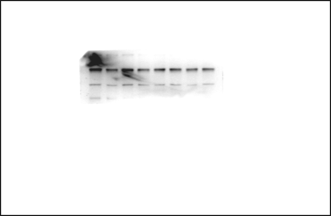

Supplement: Figure 5—source data 2. — Hippocampal tissues and primary hippocampal neurons were run on the same membrane. A: lanes 1: primary hippocampal neurons control; lanes 2: primary hippocampal neurons infected with GFP adenovirus; lanes 3: primary hippocampal neurons infected with overexpressing Kallistatin adenovirus. E, F, G: lanes 1: primary hippocampal neurons infected with GFP adenovirus; lanes 2: primary hippocampal neurons infected with overexpressing Kallistatin adenovirus; lanes 3: primary hippocampal neurons infected with overexpressing Kallistatin adenovirus and treated with Verubecestat/siHESl/RSG; K: lanes 1-4: hippocampus from WT mice; lanes 5-8: hippocampusfom KAL-TG mice. [file elife-99462-fig5-data2.zip › Fig5G YY1 hippocampal tissue.tif]

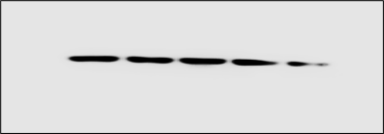

Supplement: Figure 5—source data 2. — Hippocampal tissues and primary hippocampal neurons were run on the same membrane. A: lanes 1: primary hippocampal neurons control; lanes 2: primary hippocampal neurons infected with GFP adenovirus; lanes 3: primary hippocampal neurons infected with overexpressing Kallistatin adenovirus. E, F, G: lanes 1: primary hippocampal neurons infected with GFP adenovirus; lanes 2: primary hippocampal neurons infected with overexpressing Kallistatin adenovirus; lanes 3: primary hippocampal neurons infected with overexpressing Kallistatin adenovirus and treated with Verubecestat/siHESl/RSG; K: lanes 1-4: hippocampus from WT mice; lanes 5-8: hippocampusfom KAL-TG mice. [file elife-99462-fig5-data2.zip › Fig5K Actin Neuron.tif]

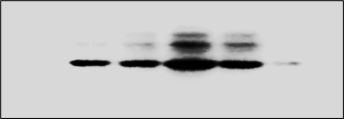

Supplement: Figure 5—source data 2. — Hippocampal tissues and primary hippocampal neurons were run on the same membrane. A: lanes 1: primary hippocampal neurons control; lanes 2: primary hippocampal neurons infected with GFP adenovirus; lanes 3: primary hippocampal neurons infected with overexpressing Kallistatin adenovirus. E, F, G: lanes 1: primary hippocampal neurons infected with GFP adenovirus; lanes 2: primary hippocampal neurons infected with overexpressing Kallistatin adenovirus; lanes 3: primary hippocampal neurons infected with overexpressing Kallistatin adenovirus and treated with Verubecestat/siHESl/RSG; K: lanes 1-4: hippocampus from WT mice; lanes 5-8: hippocampusfom KAL-TG mice. [file elife-99462-fig5-data2.zip › Fig5K BACE1 Neuron.tif]

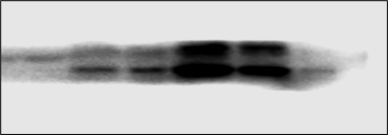

Supplement: Figure 5—source data 2. — Hippocampal tissues and primary hippocampal neurons were run on the same membrane. A: lanes 1: primary hippocampal neurons control; lanes 2: primary hippocampal neurons infected with GFP adenovirus; lanes 3: primary hippocampal neurons infected with overexpressing Kallistatin adenovirus. E, F, G: lanes 1: primary hippocampal neurons infected with GFP adenovirus; lanes 2: primary hippocampal neurons infected with overexpressing Kallistatin adenovirus; lanes 3: primary hippocampal neurons infected with overexpressing Kallistatin adenovirus and treated with Verubecestat/siHESl/RSG; K: lanes 1-4: hippocampus from WT mice; lanes 5-8: hippocampusfom KAL-TG mice. [file elife-99462-fig5-data2.zip › Fig5K KAL Neuron.tif]

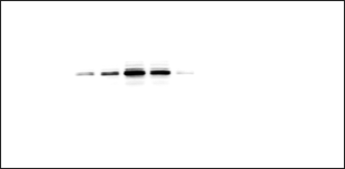

Supplement: Figure 5—source data 2. — Hippocampal tissues and primary hippocampal neurons were run on the same membrane. A: lanes 1: primary hippocampal neurons control; lanes 2: primary hippocampal neurons infected with GFP adenovirus; lanes 3: primary hippocampal neurons infected with overexpressing Kallistatin adenovirus. E, F, G: lanes 1: primary hippocampal neurons infected with GFP adenovirus; lanes 2: primary hippocampal neurons infected with overexpressing Kallistatin adenovirus; lanes 3: primary hippocampal neurons infected with overexpressing Kallistatin adenovirus and treated with Verubecestat/siHESl/RSG; K: lanes 1-4: hippocampus from WT mice; lanes 5-8: hippocampusfom KAL-TG mice. [file elife-99462-fig5-data2.zip › Fig5E Aα╕åα╕ó Neuron.tif]

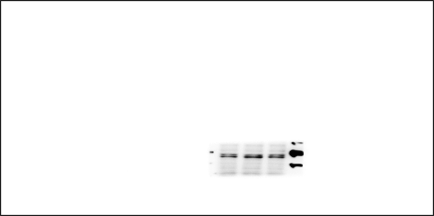

Supplement: Figure 5—source data 2. — Hippocampal tissues and primary hippocampal neurons were run on the same membrane. A: lanes 1: primary hippocampal neurons control; lanes 2: primary hippocampal neurons infected with GFP adenovirus; lanes 3: primary hippocampal neurons infected with overexpressing Kallistatin adenovirus. E, F, G: lanes 1: primary hippocampal neurons infected with GFP adenovirus; lanes 2: primary hippocampal neurons infected with overexpressing Kallistatin adenovirus; lanes 3: primary hippocampal neurons infected with overexpressing Kallistatin adenovirus and treated with Verubecestat/siHESl/RSG; K: lanes 1-4: hippocampus from WT mice; lanes 5-8: hippocampusfom KAL-TG mice. [file elife-99462-fig5-data2.zip › Fig5F Aα╕åα╕ó Neuron.tif]

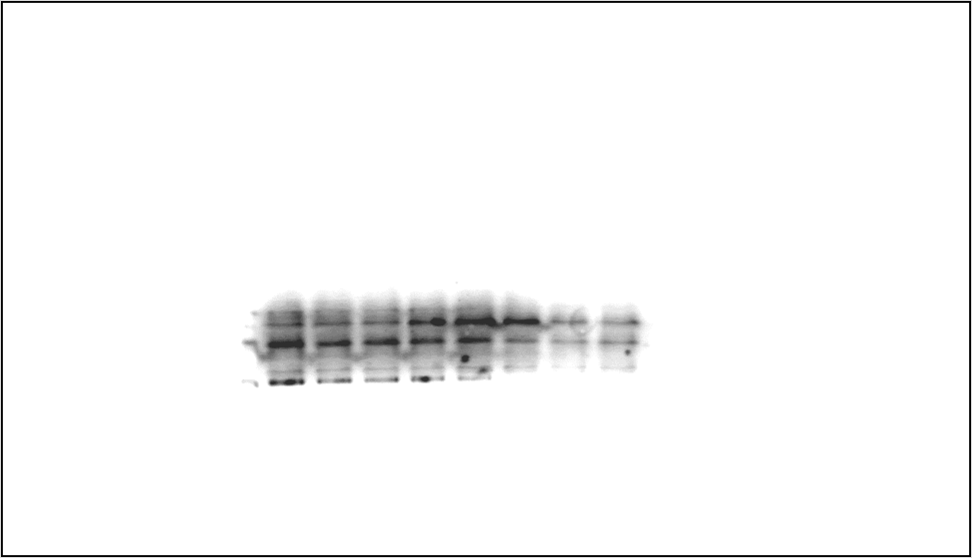

Supplement: Figure 5—source data 2. — Hippocampal tissues and primary hippocampal neurons were run on the same membrane. A: lanes 1: primary hippocampal neurons control; lanes 2: primary hippocampal neurons infected with GFP adenovirus; lanes 3: primary hippocampal neurons infected with overexpressing Kallistatin adenovirus. E, F, G: lanes 1: primary hippocampal neurons infected with GFP adenovirus; lanes 2: primary hippocampal neurons infected with overexpressing Kallistatin adenovirus; lanes 3: primary hippocampal neurons infected with overexpressing Kallistatin adenovirus and treated with Verubecestat/siHESl/RSG; K: lanes 1-4: hippocampus from WT mice; lanes 5-8: hippocampusfom KAL-TG mice. [file elife-99462-fig5-data2.zip › Fig5G PPARα╕åα╕ú hippocampal tissue.tif]

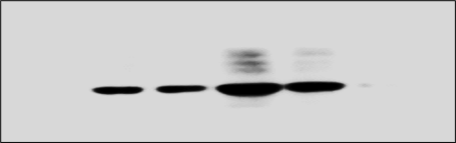

Supplement: Figure 5—source data 2. — Hippocampal tissues and primary hippocampal neurons were run on the same membrane. A: lanes 1: primary hippocampal neurons control; lanes 2: primary hippocampal neurons infected with GFP adenovirus; lanes 3: primary hippocampal neurons infected with overexpressing Kallistatin adenovirus. E, F, G: lanes 1: primary hippocampal neurons infected with GFP adenovirus; lanes 2: primary hippocampal neurons infected with overexpressing Kallistatin adenovirus; lanes 3: primary hippocampal neurons infected with overexpressing Kallistatin adenovirus and treated with Verubecestat/siHESl/RSG; K: lanes 1-4: hippocampus from WT mice; lanes 5-8: hippocampusfom KAL-TG mice. [file elife-99462-fig5-data2.zip › Fig5K Aα╕åα╕ó Neuron.tif]

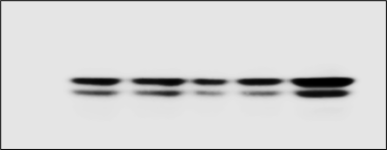

Supplement: Figure 5—source data 2. — Hippocampal tissues and primary hippocampal neurons were run on the same membrane. A: lanes 1: primary hippocampal neurons control; lanes 2: primary hippocampal neurons infected with GFP adenovirus; lanes 3: primary hippocampal neurons infected with overexpressing Kallistatin adenovirus. E, F, G: lanes 1: primary hippocampal neurons infected with GFP adenovirus; lanes 2: primary hippocampal neurons infected with overexpressing Kallistatin adenovirus; lanes 3: primary hippocampal neurons infected with overexpressing Kallistatin adenovirus and treated with Verubecestat/siHESl/RSG; K: lanes 1-4: hippocampus from WT mice; lanes 5-8: hippocampusfom KAL-TG mice. [file elife-99462-fig5-data2.zip › Fig5K PPARα╕åα╕ú Neuron.tif]

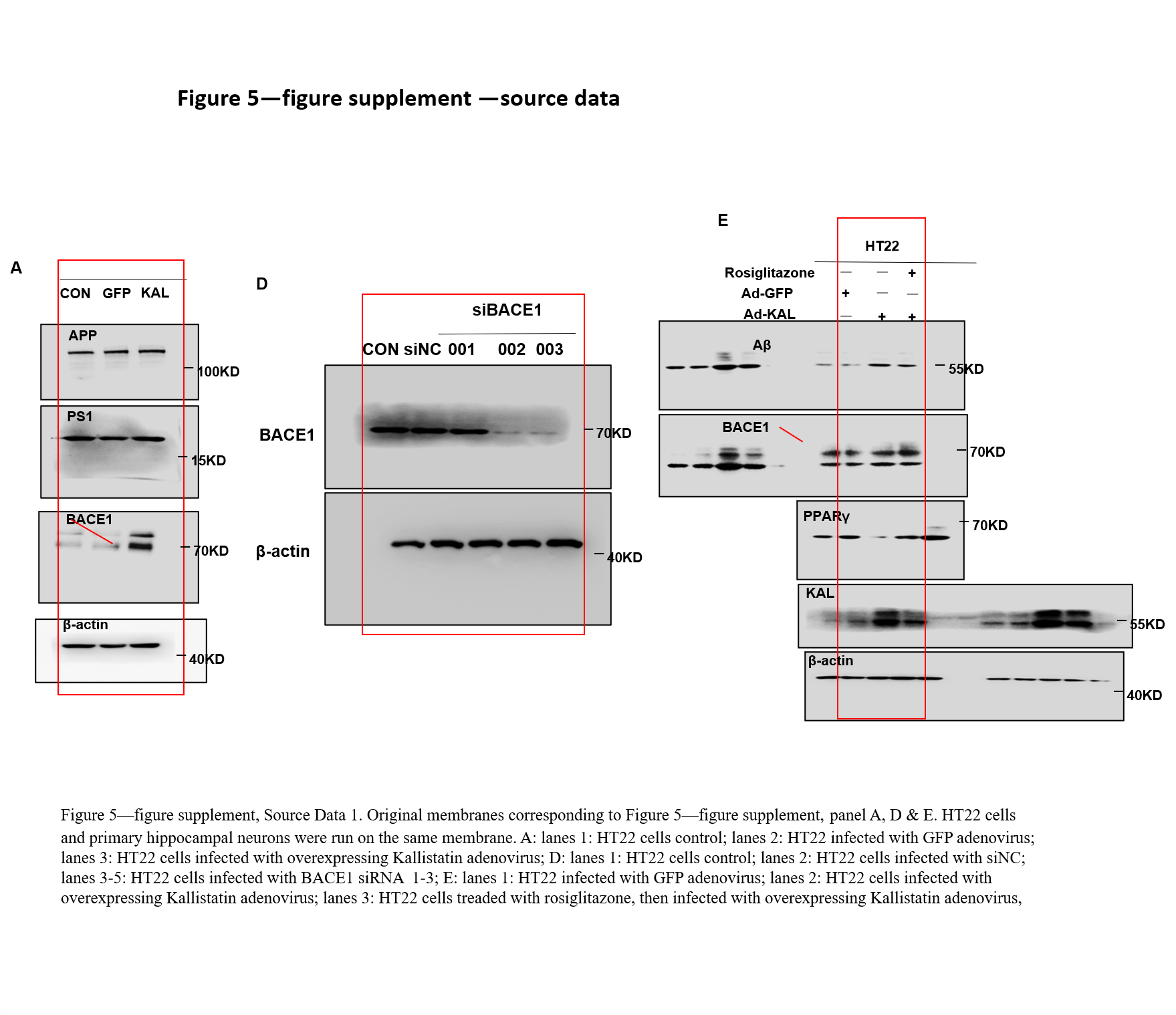

Supplement: Figure 5—figure supplement 1—source data 1. — HT22 cells and primary hippocampal neurons were run on the same membrane. A: lanes 1: HT22 cells control; lanes 2: HT22 infected with GFP adenovirus; lanes 3: 1T22 cells imfected with overexpressimg Kalistatin adenovirus; D: lanes 1: HiT22 cells control; lanes 2: HT22 cells infected with siNC; lanes 3-5: HT22 cells infected with BACE1 siRNA 1-3; E: lanes 1: HT22 infected with GFP adenovirus; lanes 2: HT22 cells infected with overexpressing Kalistatin adenovirus; lanes 3: HT22 cels treaded with rosiglitazone, then infected with overexpressing Kallistatin adenovirus. [file elife-99462-fig5-figsupp1-data1.zip › Figure 5-figure supplement-source data 1/Figure 5-figure supplement-source data.png]

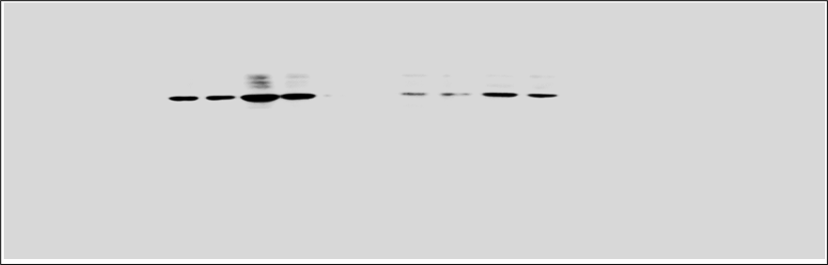

Supplement: Figure 5—figure supplement 1—source data 2. — HT22 cells and primary hippocampal neurons were run on the same membrane. A: lanes 1: HT22 cells control; lanes 2: HT22 infected with GFP adenovirus; lanes 3: 1T22 cells imfected with overexpressimg Kalistatin adenovirus; D: lanes 1: HiT22 cells control; lanes 2: HT22 cells infected with siNC; lanes 3-5: HT22 cells infected with BACE1 siRNA 1-3; E: lanes 1: HT22 infected with GFP adenovirus; lanes 2: HT22 cells infected with overexpressing Kalistatin adenovirus; lanes 3: HT22 cels treaded with rosiglitazone, then infected with overexpressing Kallistatin adenovirus. [file elife-99462-fig5-figsupp1-data2.zip › Fig.S5E Aα╕åα╕ó HT22.tif]

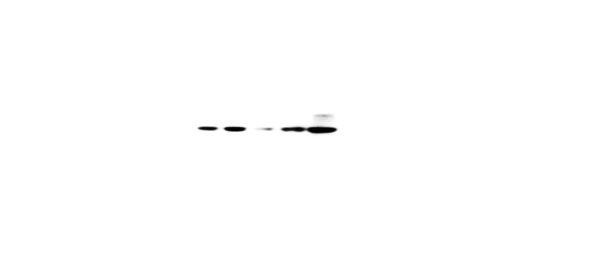

Supplement: Figure 5—figure supplement 1—source data 2. — HT22 cells and primary hippocampal neurons were run on the same membrane. A: lanes 1: HT22 cells control; lanes 2: HT22 infected with GFP adenovirus; lanes 3: 1T22 cells imfected with overexpressimg Kalistatin adenovirus; D: lanes 1: HiT22 cells control; lanes 2: HT22 cells infected with siNC; lanes 3-5: HT22 cells infected with BACE1 siRNA 1-3; E: lanes 1: HT22 infected with GFP adenovirus; lanes 2: HT22 cells infected with overexpressing Kalistatin adenovirus; lanes 3: HT22 cels treaded with rosiglitazone, then infected with overexpressing Kallistatin adenovirus. [file elife-99462-fig5-figsupp1-data2.zip › Fig.S5E PPARα╕åα╕ú HT22.tif]

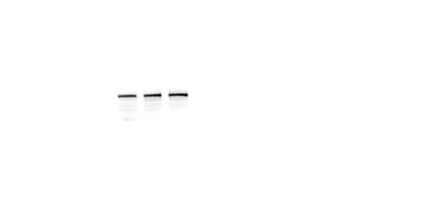

Supplement: Figure 5—figure supplement 1—source data 2. — HT22 cells and primary hippocampal neurons were run on the same membrane. A: lanes 1: HT22 cells control; lanes 2: HT22 infected with GFP adenovirus; lanes 3: 1T22 cells imfected with overexpressimg Kalistatin adenovirus; D: lanes 1: HiT22 cells control; lanes 2: HT22 cells infected with siNC; lanes 3-5: HT22 cells infected with BACE1 siRNA 1-3; E: lanes 1: HT22 infected with GFP adenovirus; lanes 2: HT22 cells infected with overexpressing Kalistatin adenovirus; lanes 3: HT22 cels treaded with rosiglitazone, then infected with overexpressing Kallistatin adenovirus. [file elife-99462-fig5-figsupp1-data2.zip › Fig.S5A APP HT22.tif]

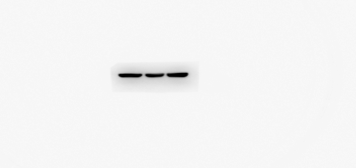

Supplement: Figure 5—figure supplement 1—source data 2. — HT22 cells and primary hippocampal neurons were run on the same membrane. A: lanes 1: HT22 cells control; lanes 2: HT22 infected with GFP adenovirus; lanes 3: 1T22 cells imfected with overexpressimg Kalistatin adenovirus; D: lanes 1: HiT22 cells control; lanes 2: HT22 cells infected with siNC; lanes 3-5: HT22 cells infected with BACE1 siRNA 1-3; E: lanes 1: HT22 infected with GFP adenovirus; lanes 2: HT22 cells infected with overexpressing Kalistatin adenovirus; lanes 3: HT22 cels treaded with rosiglitazone, then infected with overexpressing Kallistatin adenovirus. [file elife-99462-fig5-figsupp1-data2.zip › Fig.S5A Actin HT22.tif]

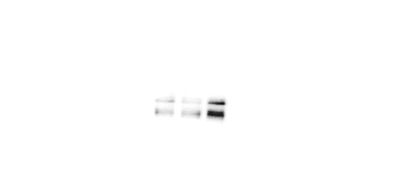

Supplement: Figure 5—figure supplement 1—source data 2. — HT22 cells and primary hippocampal neurons were run on the same membrane. A: lanes 1: HT22 cells control; lanes 2: HT22 infected with GFP adenovirus; lanes 3: 1T22 cells imfected with overexpressimg Kalistatin adenovirus; D: lanes 1: HiT22 cells control; lanes 2: HT22 cells infected with siNC; lanes 3-5: HT22 cells infected with BACE1 siRNA 1-3; E: lanes 1: HT22 infected with GFP adenovirus; lanes 2: HT22 cells infected with overexpressing Kalistatin adenovirus; lanes 3: HT22 cels treaded with rosiglitazone, then infected with overexpressing Kallistatin adenovirus. [file elife-99462-fig5-figsupp1-data2.zip › Fig.S5A KAL HT22.tif]

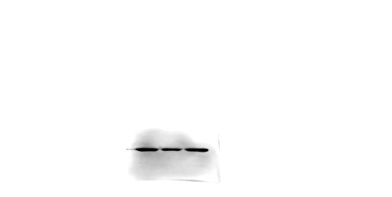

Supplement: Figure 5—figure supplement 1—source data 2. — HT22 cells and primary hippocampal neurons were run on the same membrane. A: lanes 1: HT22 cells control; lanes 2: HT22 infected with GFP adenovirus; lanes 3: 1T22 cells imfected with overexpressimg Kalistatin adenovirus; D: lanes 1: HiT22 cells control; lanes 2: HT22 cells infected with siNC; lanes 3-5: HT22 cells infected with BACE1 siRNA 1-3; E: lanes 1: HT22 infected with GFP adenovirus; lanes 2: HT22 cells infected with overexpressing Kalistatin adenovirus; lanes 3: HT22 cels treaded with rosiglitazone, then infected with overexpressing Kallistatin adenovirus. [file elife-99462-fig5-figsupp1-data2.zip › Fig.S5A PS1 HT22.tif]

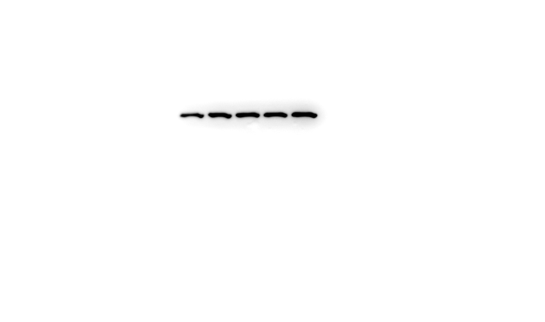

Supplement: Figure 5—figure supplement 1—source data 2. — HT22 cells and primary hippocampal neurons were run on the same membrane. A: lanes 1: HT22 cells control; lanes 2: HT22 infected with GFP adenovirus; lanes 3: 1T22 cells imfected with overexpressimg Kalistatin adenovirus; D: lanes 1: HiT22 cells control; lanes 2: HT22 cells infected with siNC; lanes 3-5: HT22 cells infected with BACE1 siRNA 1-3; E: lanes 1: HT22 infected with GFP adenovirus; lanes 2: HT22 cells infected with overexpressing Kalistatin adenovirus; lanes 3: HT22 cels treaded with rosiglitazone, then infected with overexpressing Kallistatin adenovirus. [file elife-99462-fig5-figsupp1-data2.zip › Fig.S5D Actin HT22.tif]

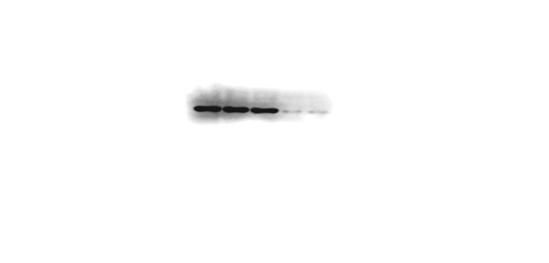

Supplement: Figure 5—figure supplement 1—source data 2. — HT22 cells and primary hippocampal neurons were run on the same membrane. A: lanes 1: HT22 cells control; lanes 2: HT22 infected with GFP adenovirus; lanes 3: 1T22 cells imfected with overexpressimg Kalistatin adenovirus; D: lanes 1: HiT22 cells control; lanes 2: HT22 cells infected with siNC; lanes 3-5: HT22 cells infected with BACE1 siRNA 1-3; E: lanes 1: HT22 infected with GFP adenovirus; lanes 2: HT22 cells infected with overexpressing Kalistatin adenovirus; lanes 3: HT22 cels treaded with rosiglitazone, then infected with overexpressing Kallistatin adenovirus. [file elife-99462-fig5-figsupp1-data2.zip › Fig.S5D BACE1 HT22.tif]

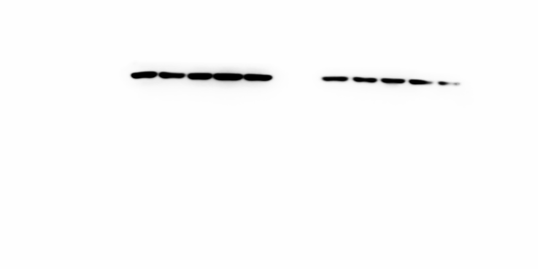

Supplement: Figure 5—figure supplement 1—source data 2. — HT22 cells and primary hippocampal neurons were run on the same membrane. A: lanes 1: HT22 cells control; lanes 2: HT22 infected with GFP adenovirus; lanes 3: 1T22 cells imfected with overexpressimg Kalistatin adenovirus; D: lanes 1: HiT22 cells control; lanes 2: HT22 cells infected with siNC; lanes 3-5: HT22 cells infected with BACE1 siRNA 1-3; E: lanes 1: HT22 infected with GFP adenovirus; lanes 2: HT22 cells infected with overexpressing Kalistatin adenovirus; lanes 3: HT22 cels treaded with rosiglitazone, then infected with overexpressing Kallistatin adenovirus. [file elife-99462-fig5-figsupp1-data2.zip › Fig.S5E Actin HT22.tif]

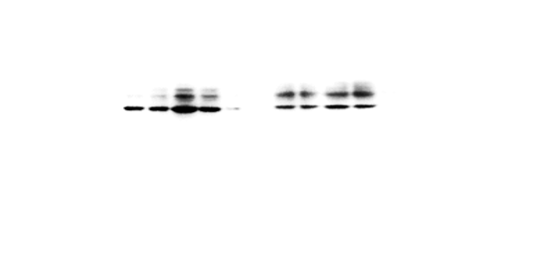

Supplement: Figure 5—figure supplement 1—source data 2. — HT22 cells and primary hippocampal neurons were run on the same membrane. A: lanes 1: HT22 cells control; lanes 2: HT22 infected with GFP adenovirus; lanes 3: 1T22 cells imfected with overexpressimg Kalistatin adenovirus; D: lanes 1: HiT22 cells control; lanes 2: HT22 cells infected with siNC; lanes 3-5: HT22 cells infected with BACE1 siRNA 1-3; E: lanes 1: HT22 infected with GFP adenovirus; lanes 2: HT22 cells infected with overexpressing Kalistatin adenovirus; lanes 3: HT22 cels treaded with rosiglitazone, then infected with overexpressing Kallistatin adenovirus. [file elife-99462-fig5-figsupp1-data2.zip › Fig.S5E BACE1 HT22.tif]

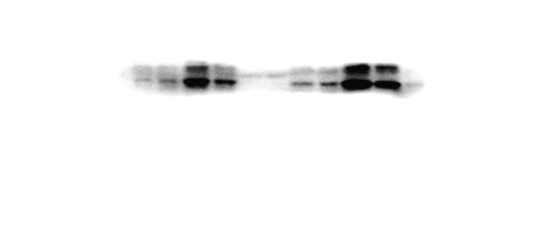

Supplement: Figure 5—figure supplement 1—source data 2. — HT22 cells and primary hippocampal neurons were run on the same membrane. A: lanes 1: HT22 cells control; lanes 2: HT22 infected with GFP adenovirus; lanes 3: 1T22 cells imfected with overexpressimg Kalistatin adenovirus; D: lanes 1: HiT22 cells control; lanes 2: HT22 cells infected with siNC; lanes 3-5: HT22 cells infected with BACE1 siRNA 1-3; E: lanes 1: HT22 infected with GFP adenovirus; lanes 2: HT22 cells infected with overexpressing Kalistatin adenovirus; lanes 3: HT22 cels treaded with rosiglitazone, then infected with overexpressing Kallistatin adenovirus. [file elife-99462-fig5-figsupp1-data2.zip › Fig.S5E KAL HT22.tif]

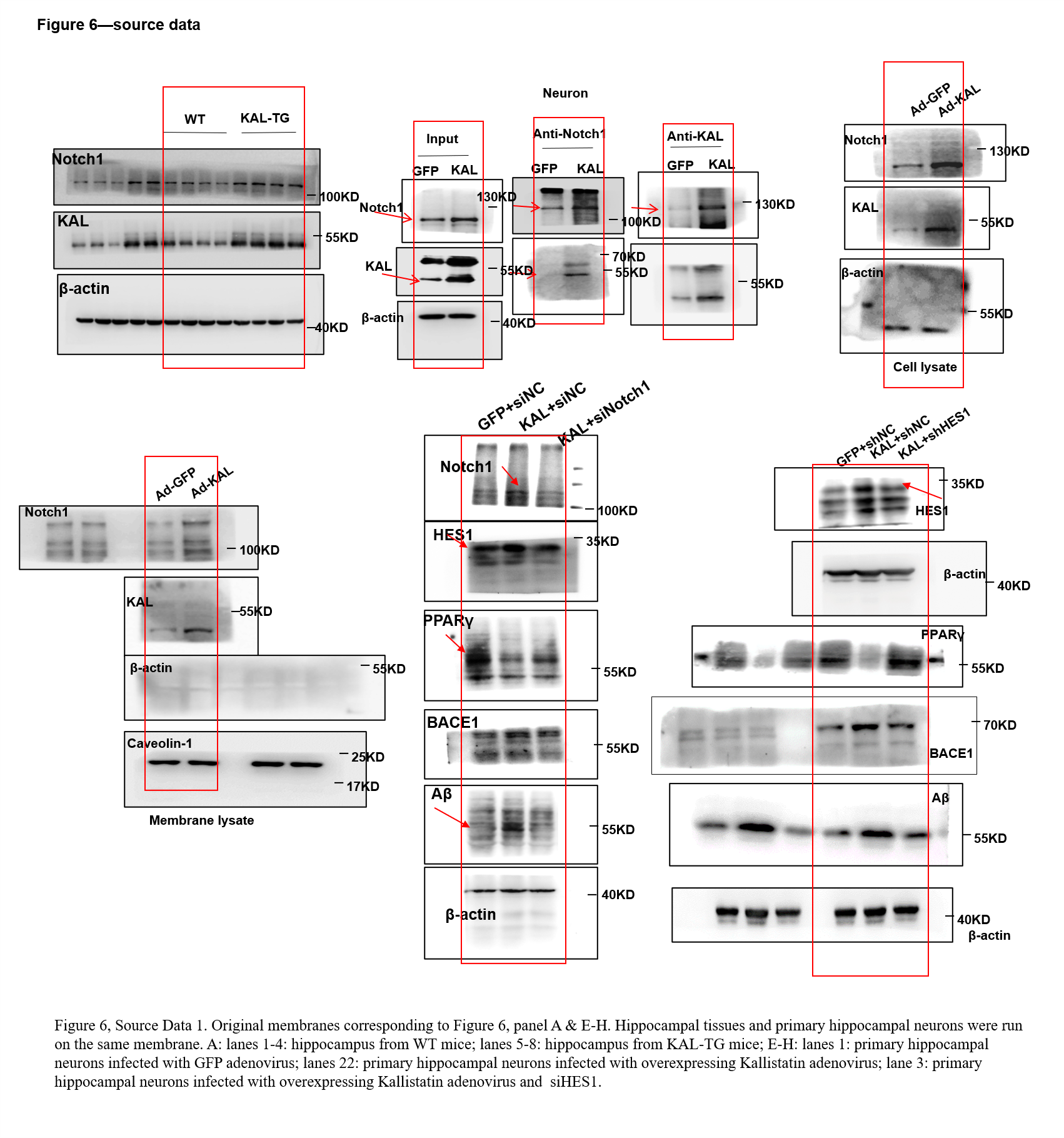

Supplement: Figure 6—source data 1. — Hippocampal tissues and primary hippocampal neurons were run on the same membrane. A: lanes 1-4: hippocampus from WT mice; lanes 5-8: hippocampus from KAL-TG mice; E–H: lanes 1: primary hippocampal neurons infected with GFP adenovirnus, lanes 2: primary hippocampal neurons infected with overexpressing Kallistatin adenovirus; lane 3: primary hippocampal neurons infected with overexpressing Kallistatin adenovirus and siHES1. [file elife-99462-fig6-data1.zip › Figure 6-source data 1/Figure 6-source data.png]

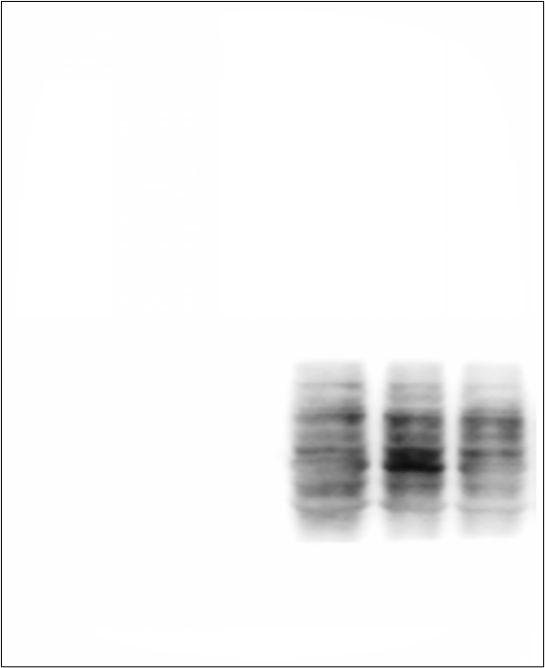

Supplement: Figure 6—source data 2. — Hippocampal tissues and primary hippocampal neurons were run on the same membrane. A: lanes 1-4: hippocampus from WT mice; lanes 5-8: hippocampus from KAL-TG mice; E–H: lanes 1: primary hippocampal neurons infected with GFP adenovirnus, lanes 2: primary hippocampal neurons infected with overexpressing Kallistatin adenovirus; lane 3: primary hippocampal neurons infected with overexpressing Kallistatin adenovirus and siHES1. [file elife-99462-fig6-data2.zip › Fig.6G Aα╕åα╕ó Neuron.tif]

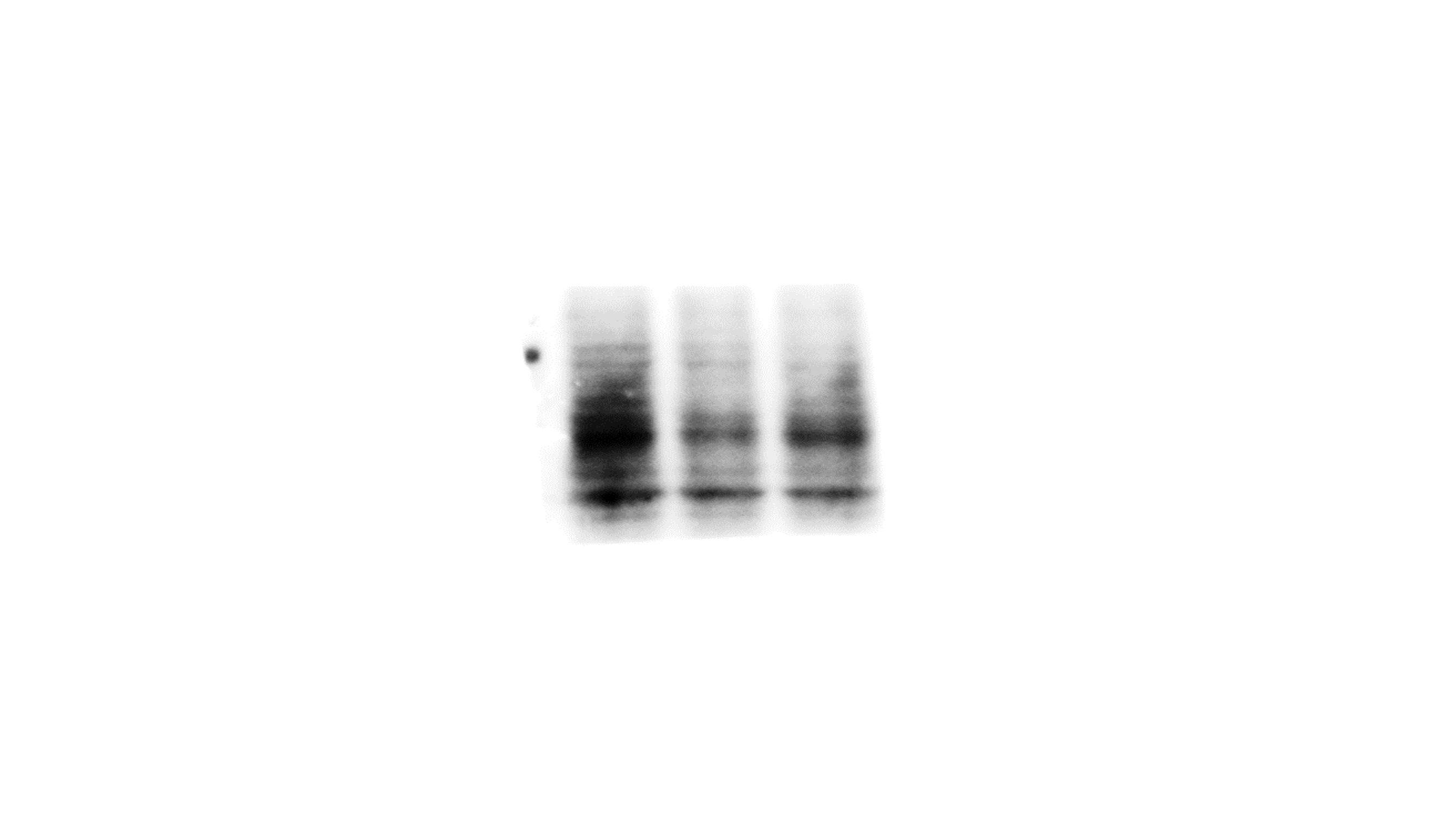

Supplement: Figure 6—source data 2. — Hippocampal tissues and primary hippocampal neurons were run on the same membrane. A: lanes 1-4: hippocampus from WT mice; lanes 5-8: hippocampus from KAL-TG mice; E–H: lanes 1: primary hippocampal neurons infected with GFP adenovirnus, lanes 2: primary hippocampal neurons infected with overexpressing Kallistatin adenovirus; lane 3: primary hippocampal neurons infected with overexpressing Kallistatin adenovirus and siHES1. [file elife-99462-fig6-data2.zip › Fig.6G PPARα╕åα╕ú Neuron.tif]

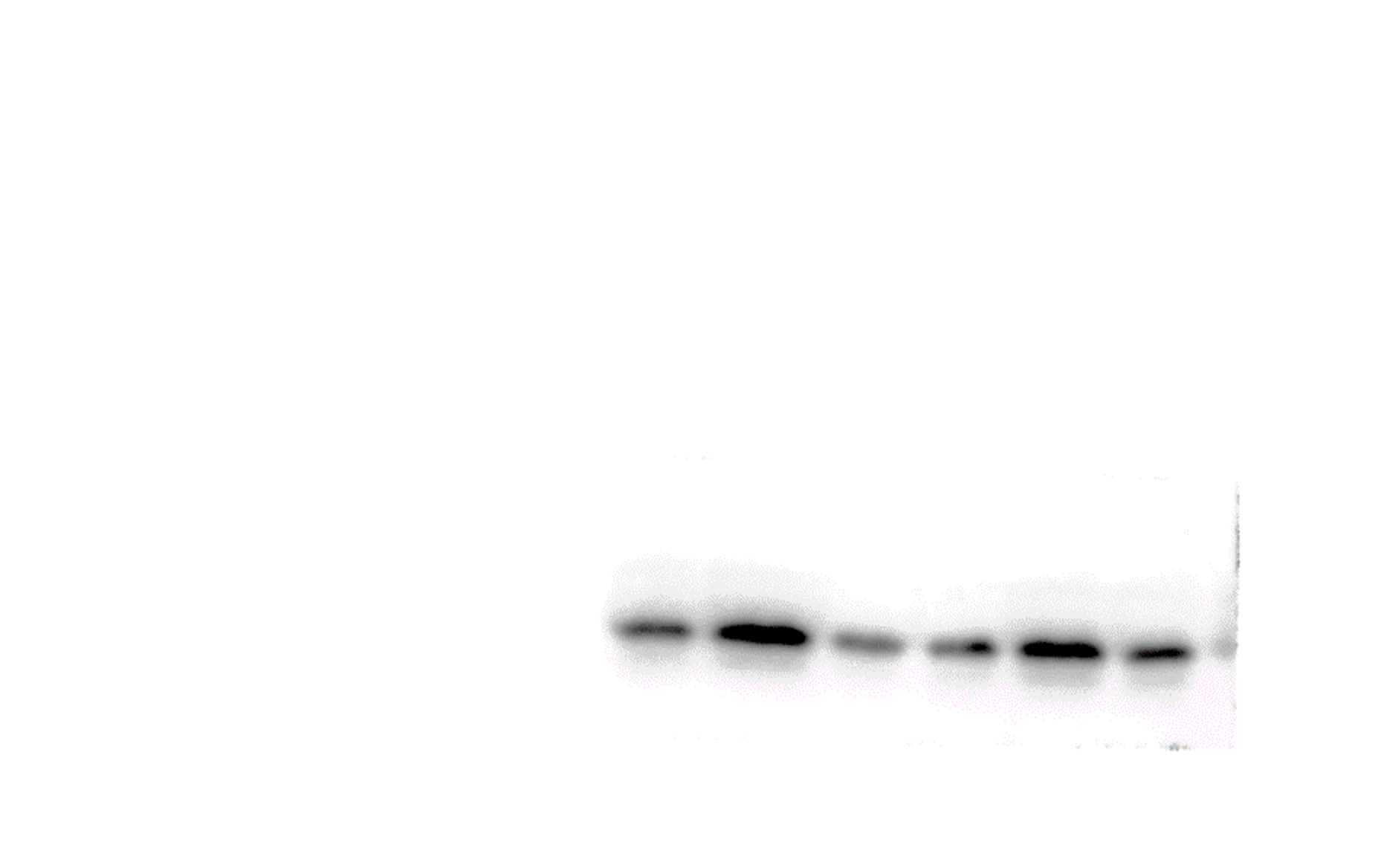

Supplement: Figure 6—source data 2. — Hippocampal tissues and primary hippocampal neurons were run on the same membrane. A: lanes 1-4: hippocampus from WT mice; lanes 5-8: hippocampus from KAL-TG mice; E–H: lanes 1: primary hippocampal neurons infected with GFP adenovirnus, lanes 2: primary hippocampal neurons infected with overexpressing Kallistatin adenovirus; lane 3: primary hippocampal neurons infected with overexpressing Kallistatin adenovirus and siHES1. [file elife-99462-fig6-data2.zip › Fig.6H Aα╕åα╕ó Neuron.tif]

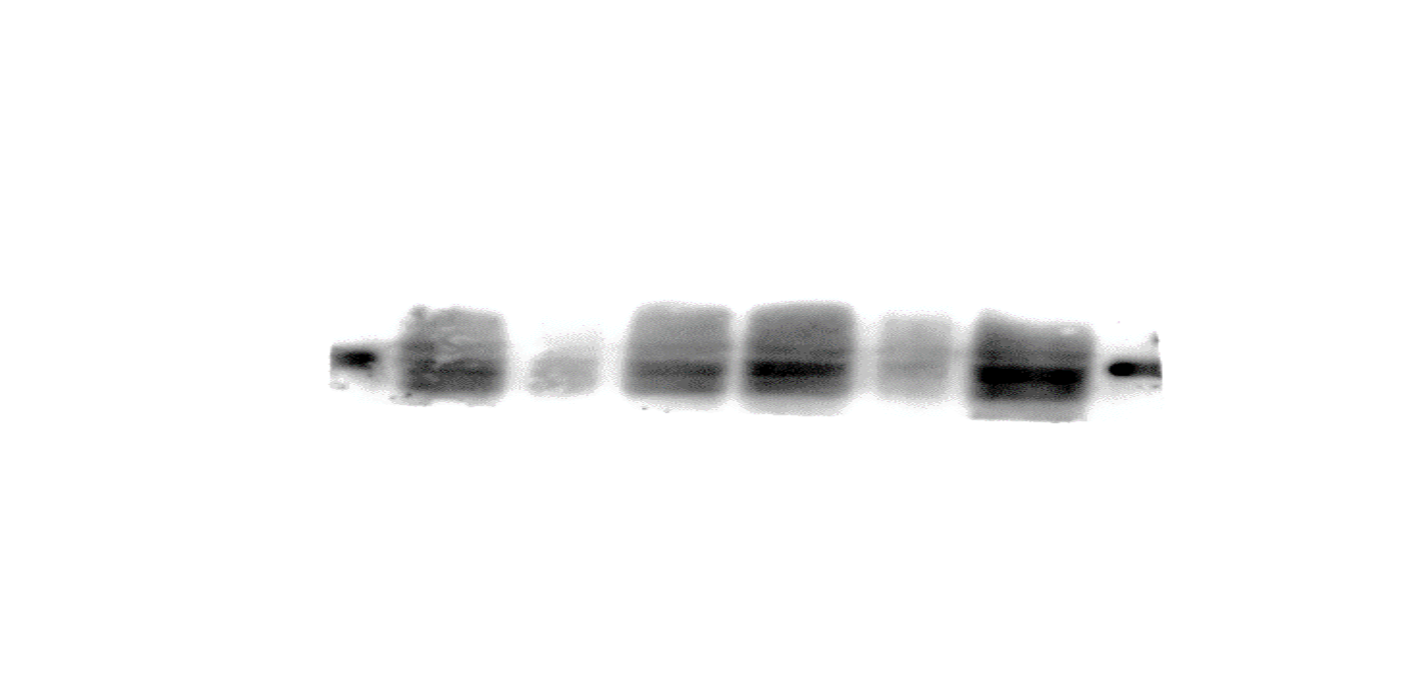

Supplement: Figure 6—source data 2. — Hippocampal tissues and primary hippocampal neurons were run on the same membrane. A: lanes 1-4: hippocampus from WT mice; lanes 5-8: hippocampus from KAL-TG mice; E–H: lanes 1: primary hippocampal neurons infected with GFP adenovirnus, lanes 2: primary hippocampal neurons infected with overexpressing Kallistatin adenovirus; lane 3: primary hippocampal neurons infected with overexpressing Kallistatin adenovirus and siHES1. [file elife-99462-fig6-data2.zip › Fig.6H PPARα╕åα╕ú Neuron.tif]

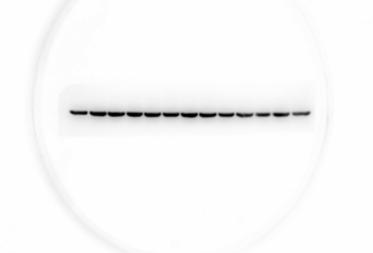

Supplement: Figure 6—source data 2. — Hippocampal tissues and primary hippocampal neurons were run on the same membrane. A: lanes 1-4: hippocampus from WT mice; lanes 5-8: hippocampus from KAL-TG mice; E–H: lanes 1: primary hippocampal neurons infected with GFP adenovirnus, lanes 2: primary hippocampal neurons infected with overexpressing Kallistatin adenovirus; lane 3: primary hippocampal neurons infected with overexpressing Kallistatin adenovirus and siHES1. [file elife-99462-fig6-data2.zip › Fig.6A Actin hippo.tif]

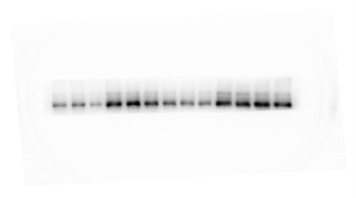

Supplement: Figure 6—source data 2. — Hippocampal tissues and primary hippocampal neurons were run on the same membrane. A: lanes 1-4: hippocampus from WT mice; lanes 5-8: hippocampus from KAL-TG mice; E–H: lanes 1: primary hippocampal neurons infected with GFP adenovirnus, lanes 2: primary hippocampal neurons infected with overexpressing Kallistatin adenovirus; lane 3: primary hippocampal neurons infected with overexpressing Kallistatin adenovirus and siHES1. [file elife-99462-fig6-data2.zip › Fig.6A KAL hippo.tif]

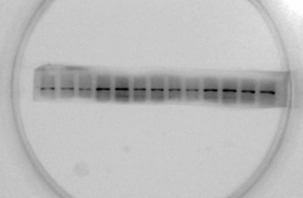

Supplement: Figure 6—source data 2. — Hippocampal tissues and primary hippocampal neurons were run on the same membrane. A: lanes 1-4: hippocampus from WT mice; lanes 5-8: hippocampus from KAL-TG mice; E–H: lanes 1: primary hippocampal neurons infected with GFP adenovirnus, lanes 2: primary hippocampal neurons infected with overexpressing Kallistatin adenovirus; lane 3: primary hippocampal neurons infected with overexpressing Kallistatin adenovirus and siHES1. [file elife-99462-fig6-data2.zip › Fig.6A Notch1 hippo.tif]

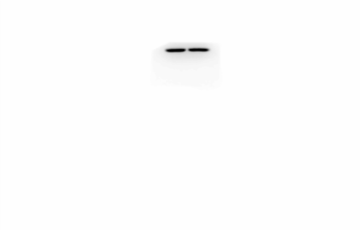

Supplement: Figure 6—source data 2. — Hippocampal tissues and primary hippocampal neurons were run on the same membrane. A: lanes 1-4: hippocampus from WT mice; lanes 5-8: hippocampus from KAL-TG mice; E–H: lanes 1: primary hippocampal neurons infected with GFP adenovirnus, lanes 2: primary hippocampal neurons infected with overexpressing Kallistatin adenovirus; lane 3: primary hippocampal neurons infected with overexpressing Kallistatin adenovirus and siHES1. [file elife-99462-fig6-data2.zip › Fig.6E Actin Neuron.tif]

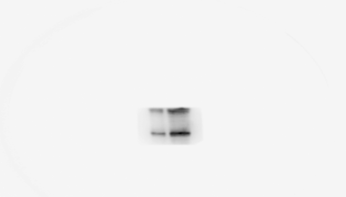

Supplement: Figure 6—source data 2. — Hippocampal tissues and primary hippocampal neurons were run on the same membrane. A: lanes 1-4: hippocampus from WT mice; lanes 5-8: hippocampus from KAL-TG mice; E–H: lanes 1: primary hippocampal neurons infected with GFP adenovirnus, lanes 2: primary hippocampal neurons infected with overexpressing Kallistatin adenovirus; lane 3: primary hippocampal neurons infected with overexpressing Kallistatin adenovirus and siHES1. [file elife-99462-fig6-data2.zip › Fig.6E KAL anti KAL Neuron.tif]

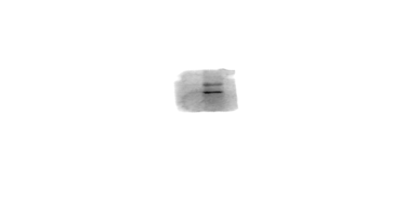

Supplement: Figure 6—source data 2. — Hippocampal tissues and primary hippocampal neurons were run on the same membrane. A: lanes 1-4: hippocampus from WT mice; lanes 5-8: hippocampus from KAL-TG mice; E–H: lanes 1: primary hippocampal neurons infected with GFP adenovirnus, lanes 2: primary hippocampal neurons infected with overexpressing Kallistatin adenovirus; lane 3: primary hippocampal neurons infected with overexpressing Kallistatin adenovirus and siHES1. [file elife-99462-fig6-data2.zip › Fig.6E KAL anti notch1 Neuron.tif]

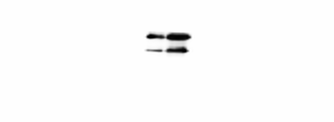

Supplement: Figure 6—source data 2. — Hippocampal tissues and primary hippocampal neurons were run on the same membrane. A: lanes 1-4: hippocampus from WT mice; lanes 5-8: hippocampus from KAL-TG mice; E–H: lanes 1: primary hippocampal neurons infected with GFP adenovirnus, lanes 2: primary hippocampal neurons infected with overexpressing Kallistatin adenovirus; lane 3: primary hippocampal neurons infected with overexpressing Kallistatin adenovirus and siHES1. [file elife-99462-fig6-data2.zip › Fig.6E KAL input Neuron.tif]

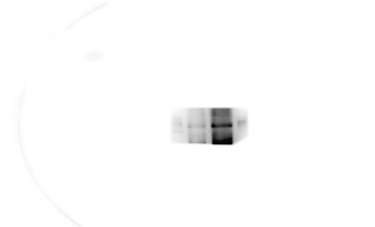

Supplement: Figure 6—source data 2. — Hippocampal tissues and primary hippocampal neurons were run on the same membrane. A: lanes 1-4: hippocampus from WT mice; lanes 5-8: hippocampus from KAL-TG mice; E–H: lanes 1: primary hippocampal neurons infected with GFP adenovirnus, lanes 2: primary hippocampal neurons infected with overexpressing Kallistatin adenovirus; lane 3: primary hippocampal neurons infected with overexpressing Kallistatin adenovirus and siHES1. [file elife-99462-fig6-data2.zip › Fig.6E Notch1 anti KAL Neuron.tif]

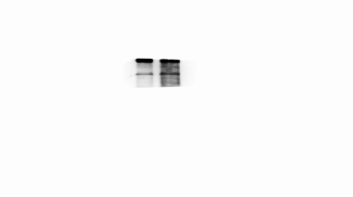

Supplement: Figure 6—source data 2. — Hippocampal tissues and primary hippocampal neurons were run on the same membrane. A: lanes 1-4: hippocampus from WT mice; lanes 5-8: hippocampus from KAL-TG mice; E–H: lanes 1: primary hippocampal neurons infected with GFP adenovirnus, lanes 2: primary hippocampal neurons infected with overexpressing Kallistatin adenovirus; lane 3: primary hippocampal neurons infected with overexpressing Kallistatin adenovirus and siHES1. [file elife-99462-fig6-data2.zip › Fig.6E Notch1 anti notch1 Neuron.tif]

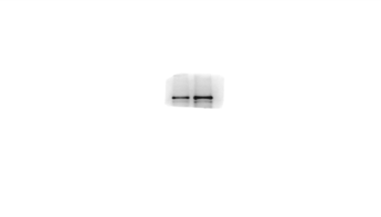

Supplement: Figure 6—source data 2. — Hippocampal tissues and primary hippocampal neurons were run on the same membrane. A: lanes 1-4: hippocampus from WT mice; lanes 5-8: hippocampus from KAL-TG mice; E–H: lanes 1: primary hippocampal neurons infected with GFP adenovirnus, lanes 2: primary hippocampal neurons infected with overexpressing Kallistatin adenovirus; lane 3: primary hippocampal neurons infected with overexpressing Kallistatin adenovirus and siHES1. [file elife-99462-fig6-data2.zip › Fig.6E Notch1 input Neuron.tif]

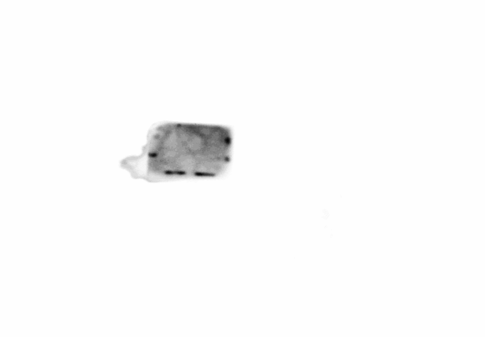

Supplement: Figure 6—source data 2. — Hippocampal tissues and primary hippocampal neurons were run on the same membrane. A: lanes 1-4: hippocampus from WT mice; lanes 5-8: hippocampus from KAL-TG mice; E–H: lanes 1: primary hippocampal neurons infected with GFP adenovirnus, lanes 2: primary hippocampal neurons infected with overexpressing Kallistatin adenovirus; lane 3: primary hippocampal neurons infected with overexpressing Kallistatin adenovirus and siHES1. [file elife-99462-fig6-data2.zip › Fig.6F Actin cell lysate Neuron.tif]

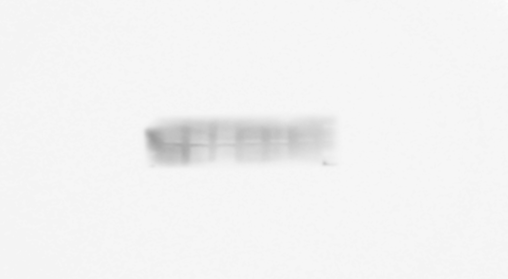

Supplement: Figure 6—source data 2. — Hippocampal tissues and primary hippocampal neurons were run on the same membrane. A: lanes 1-4: hippocampus from WT mice; lanes 5-8: hippocampus from KAL-TG mice; E–H: lanes 1: primary hippocampal neurons infected with GFP adenovirnus, lanes 2: primary hippocampal neurons infected with overexpressing Kallistatin adenovirus; lane 3: primary hippocampal neurons infected with overexpressing Kallistatin adenovirus and siHES1. [file elife-99462-fig6-data2.zip › Fig.6F Actin menbrane Neuron.tif]

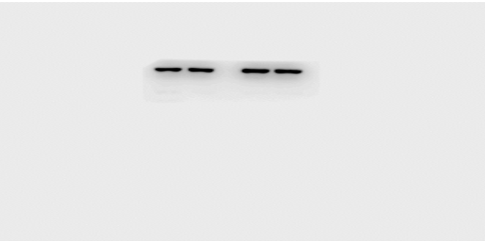

Supplement: Figure 6—source data 2. — Hippocampal tissues and primary hippocampal neurons were run on the same membrane. A: lanes 1-4: hippocampus from WT mice; lanes 5-8: hippocampus from KAL-TG mice; E–H: lanes 1: primary hippocampal neurons infected with GFP adenovirnus, lanes 2: primary hippocampal neurons infected with overexpressing Kallistatin adenovirus; lane 3: primary hippocampal neurons infected with overexpressing Kallistatin adenovirus and siHES1. [file elife-99462-fig6-data2.zip › Fig.6F Caveolin1 menbrane Neuron.tif]

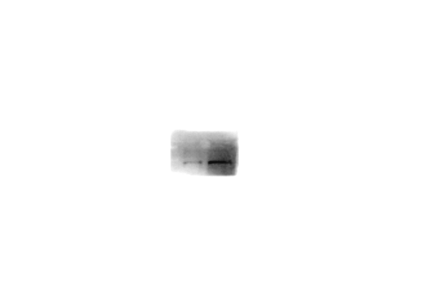

Supplement: Figure 6—source data 2. — Hippocampal tissues and primary hippocampal neurons were run on the same membrane. A: lanes 1-4: hippocampus from WT mice; lanes 5-8: hippocampus from KAL-TG mice; E–H: lanes 1: primary hippocampal neurons infected with GFP adenovirnus, lanes 2: primary hippocampal neurons infected with overexpressing Kallistatin adenovirus; lane 3: primary hippocampal neurons infected with overexpressing Kallistatin adenovirus and siHES1. [file elife-99462-fig6-data2.zip › Fig.6F KAL cell lysate Neuron.tif]

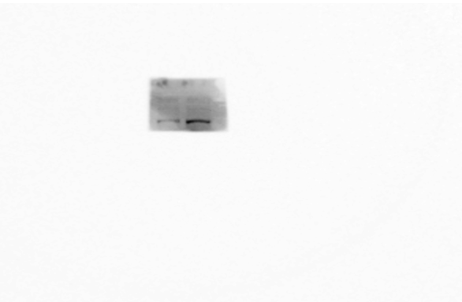

Supplement: Figure 6—source data 2. — Hippocampal tissues and primary hippocampal neurons were run on the same membrane. A: lanes 1-4: hippocampus from WT mice; lanes 5-8: hippocampus from KAL-TG mice; E–H: lanes 1: primary hippocampal neurons infected with GFP adenovirnus, lanes 2: primary hippocampal neurons infected with overexpressing Kallistatin adenovirus; lane 3: primary hippocampal neurons infected with overexpressing Kallistatin adenovirus and siHES1. [file elife-99462-fig6-data2.zip › Fig.6F KAL menbrane Neuron.tif]

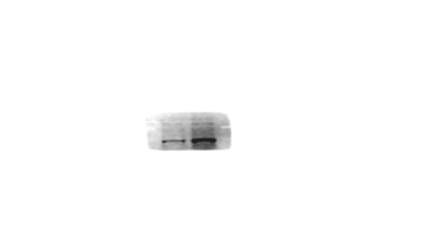

Supplement: Figure 6—source data 2. — Hippocampal tissues and primary hippocampal neurons were run on the same membrane. A: lanes 1-4: hippocampus from WT mice; lanes 5-8: hippocampus from KAL-TG mice; E–H: lanes 1: primary hippocampal neurons infected with GFP adenovirnus, lanes 2: primary hippocampal neurons infected with overexpressing Kallistatin adenovirus; lane 3: primary hippocampal neurons infected with overexpressing Kallistatin adenovirus and siHES1. [file elife-99462-fig6-data2.zip › Fig.6F Notch1 cell lysate Neuron.tif]

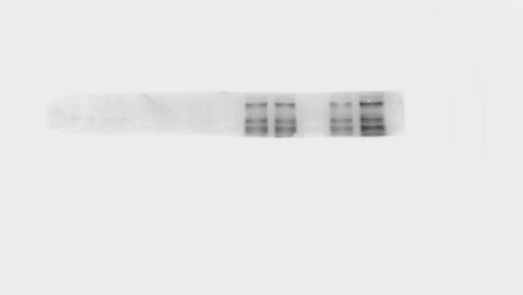

Supplement: Figure 6—source data 2. — Hippocampal tissues and primary hippocampal neurons were run on the same membrane. A: lanes 1-4: hippocampus from WT mice; lanes 5-8: hippocampus from KAL-TG mice; E–H: lanes 1: primary hippocampal neurons infected with GFP adenovirnus, lanes 2: primary hippocampal neurons infected with overexpressing Kallistatin adenovirus; lane 3: primary hippocampal neurons infected with overexpressing Kallistatin adenovirus and siHES1. [file elife-99462-fig6-data2.zip › Fig.6F Notch1 menbrane Neuron.tif]

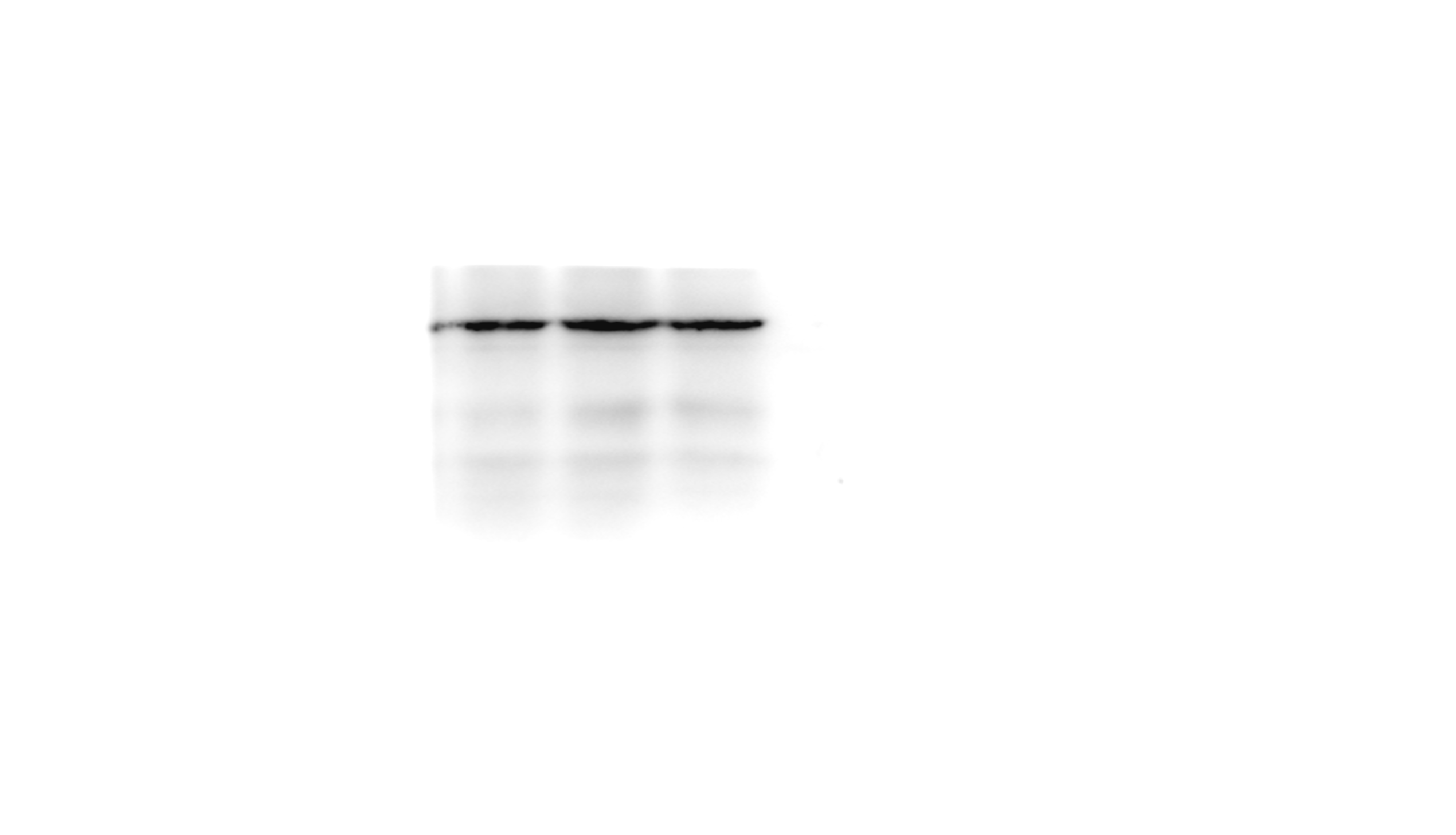

Supplement: Figure 6—source data 2. — Hippocampal tissues and primary hippocampal neurons were run on the same membrane. A: lanes 1-4: hippocampus from WT mice; lanes 5-8: hippocampus from KAL-TG mice; E–H: lanes 1: primary hippocampal neurons infected with GFP adenovirnus, lanes 2: primary hippocampal neurons infected with overexpressing Kallistatin adenovirus; lane 3: primary hippocampal neurons infected with overexpressing Kallistatin adenovirus and siHES1. [file elife-99462-fig6-data2.zip › Fig.6G Actin Neuron.tif]

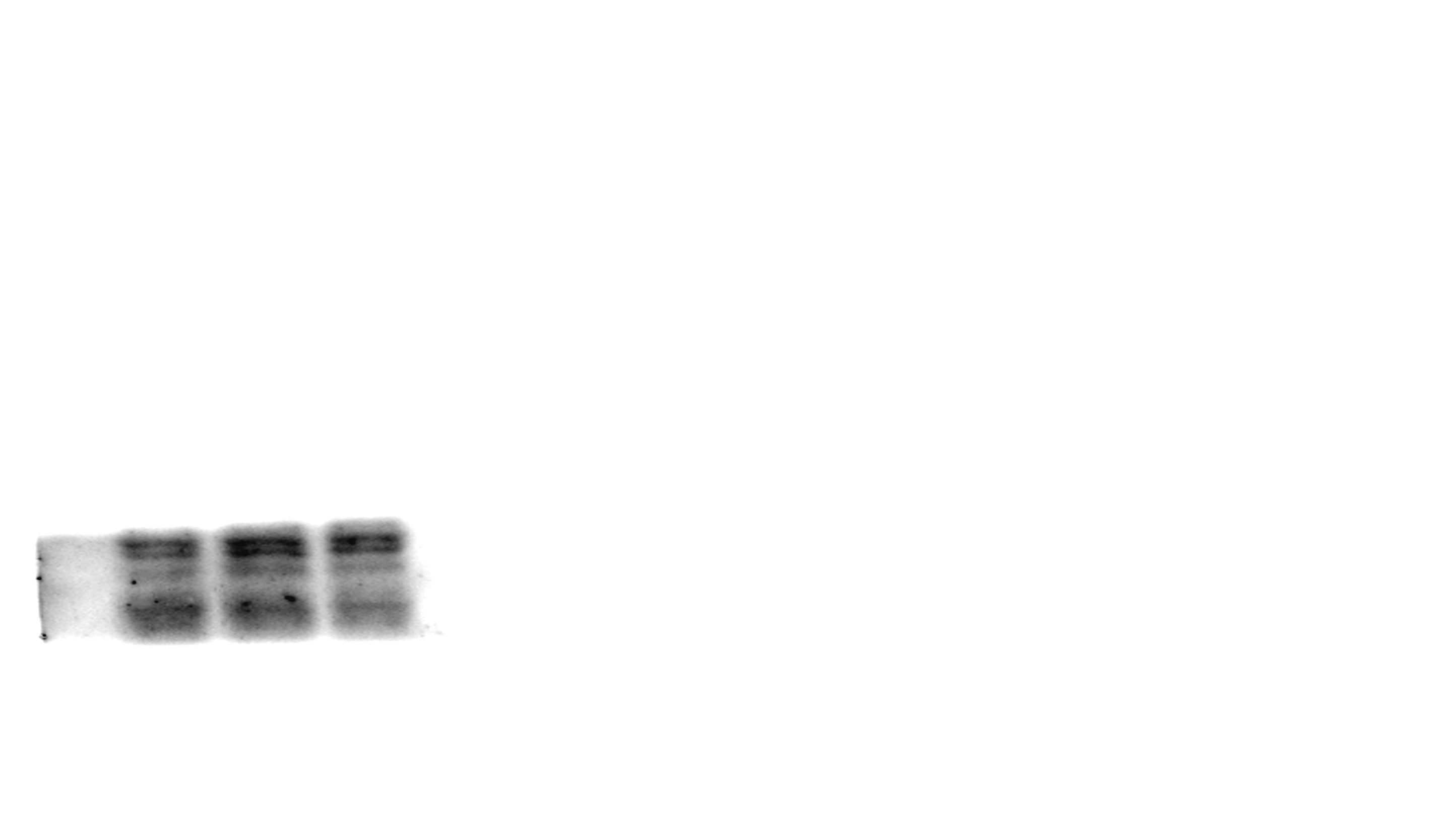

Supplement: Figure 6—source data 2. — Hippocampal tissues and primary hippocampal neurons were run on the same membrane. A: lanes 1-4: hippocampus from WT mice; lanes 5-8: hippocampus from KAL-TG mice; E–H: lanes 1: primary hippocampal neurons infected with GFP adenovirnus, lanes 2: primary hippocampal neurons infected with overexpressing Kallistatin adenovirus; lane 3: primary hippocampal neurons infected with overexpressing Kallistatin adenovirus and siHES1. [file elife-99462-fig6-data2.zip › Fig.6G BACE1 Neuron.tif]
