# Supplementary figures and images for: The novel role of Kallistatin in linking metabolic syndromes and cognitive memory deterioration by inducing amyloid-β plaques accumulation and tau protein hyperphosphorylation (part 2 of 2)
Source: eLife. 2025 Aug 5;13:RP99462. doi: 10.7554/eLife.99462 (PMC12324742; doi:10.7554/eLife.99462)

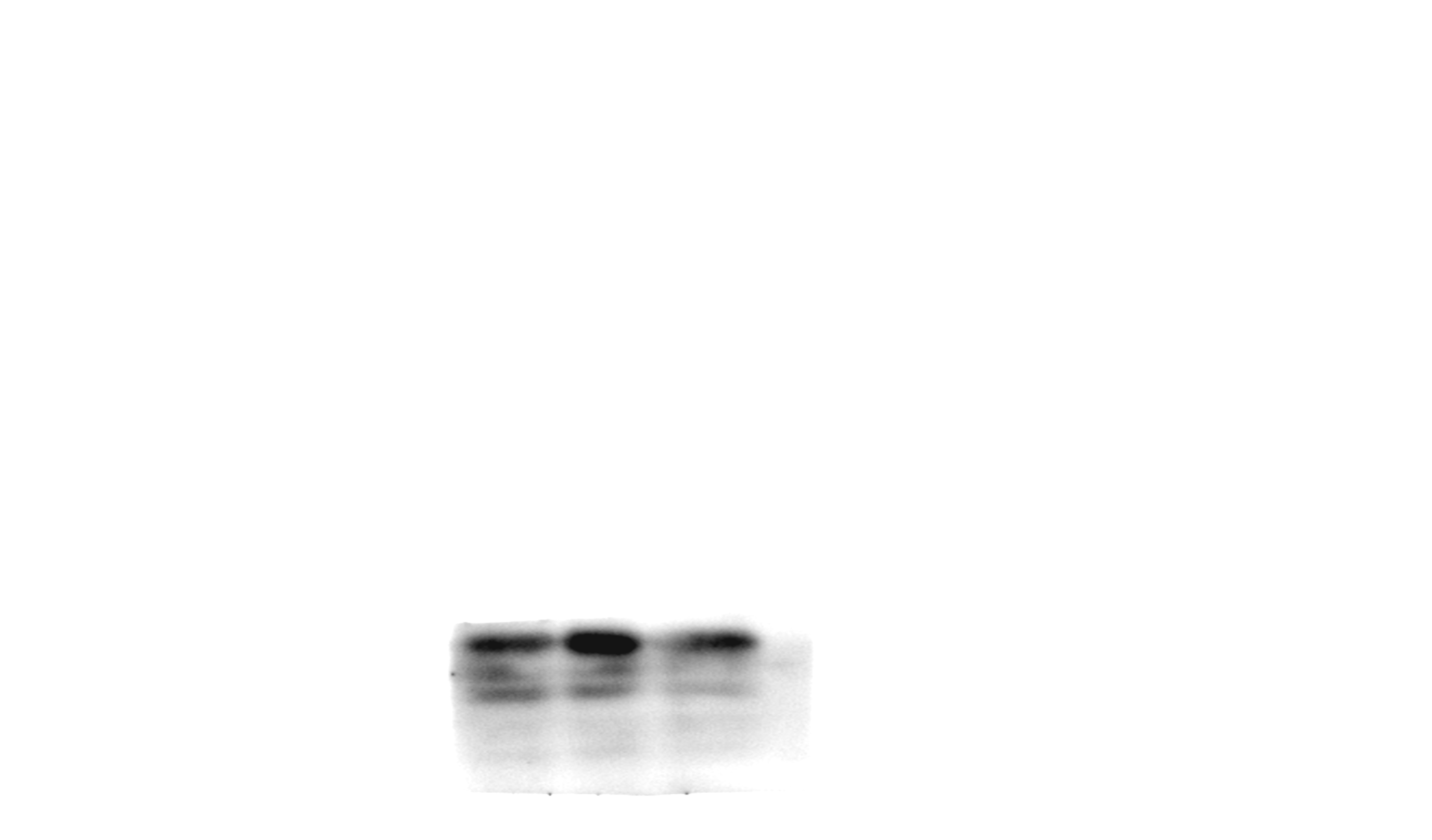

Supplement: Figure 6—source data 2. — Hippocampal tissues and primary hippocampal neurons were run on the same membrane. A: lanes 1-4: hippocampus from WT mice; lanes 5-8: hippocampus from KAL-TG mice; E–H: lanes 1: primary hippocampal neurons infected with GFP adenovirnus, lanes 2: primary hippocampal neurons infected with overexpressing Kallistatin adenovirus; lane 3: primary hippocampal neurons infected with overexpressing Kallistatin adenovirus and siHES1. [file elife-99462-fig6-data2.zip › Fig.6G HES1 Neuron.tif]

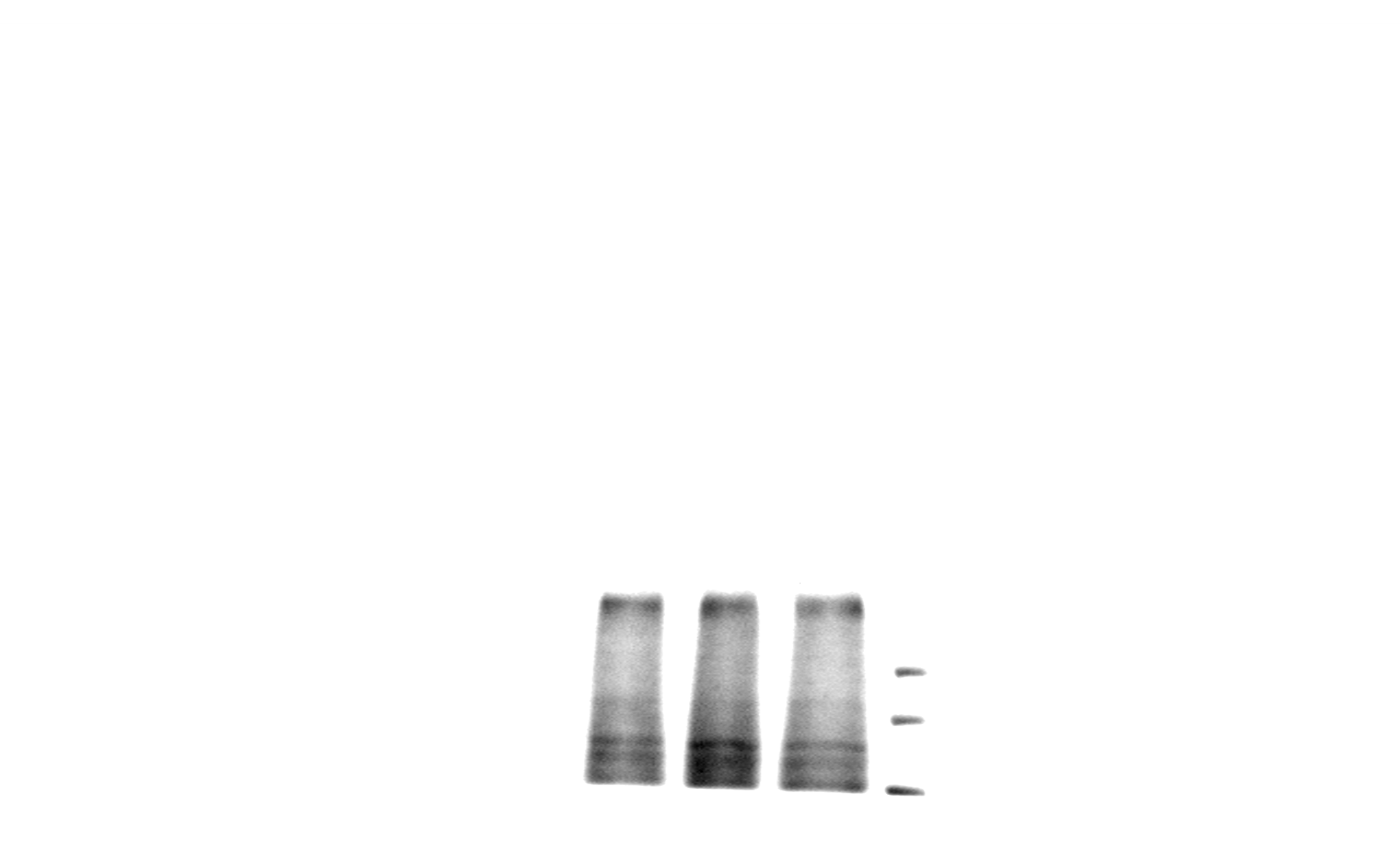

Supplement: Figure 6—source data 2. — Hippocampal tissues and primary hippocampal neurons were run on the same membrane. A: lanes 1-4: hippocampus from WT mice; lanes 5-8: hippocampus from KAL-TG mice; E–H: lanes 1: primary hippocampal neurons infected with GFP adenovirnus, lanes 2: primary hippocampal neurons infected with overexpressing Kallistatin adenovirus; lane 3: primary hippocampal neurons infected with overexpressing Kallistatin adenovirus and siHES1. [file elife-99462-fig6-data2.zip › Fig.6G Notch1 Neuron.tif]

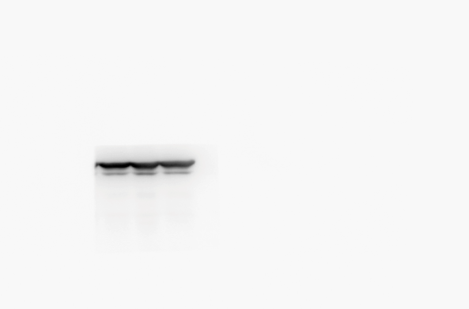

Supplement: Figure 6—source data 2. — Hippocampal tissues and primary hippocampal neurons were run on the same membrane. A: lanes 1-4: hippocampus from WT mice; lanes 5-8: hippocampus from KAL-TG mice; E–H: lanes 1: primary hippocampal neurons infected with GFP adenovirnus, lanes 2: primary hippocampal neurons infected with overexpressing Kallistatin adenovirus; lane 3: primary hippocampal neurons infected with overexpressing Kallistatin adenovirus and siHES1. [file elife-99462-fig6-data2.zip › Fig.6H Actin1 Neuron.tif]

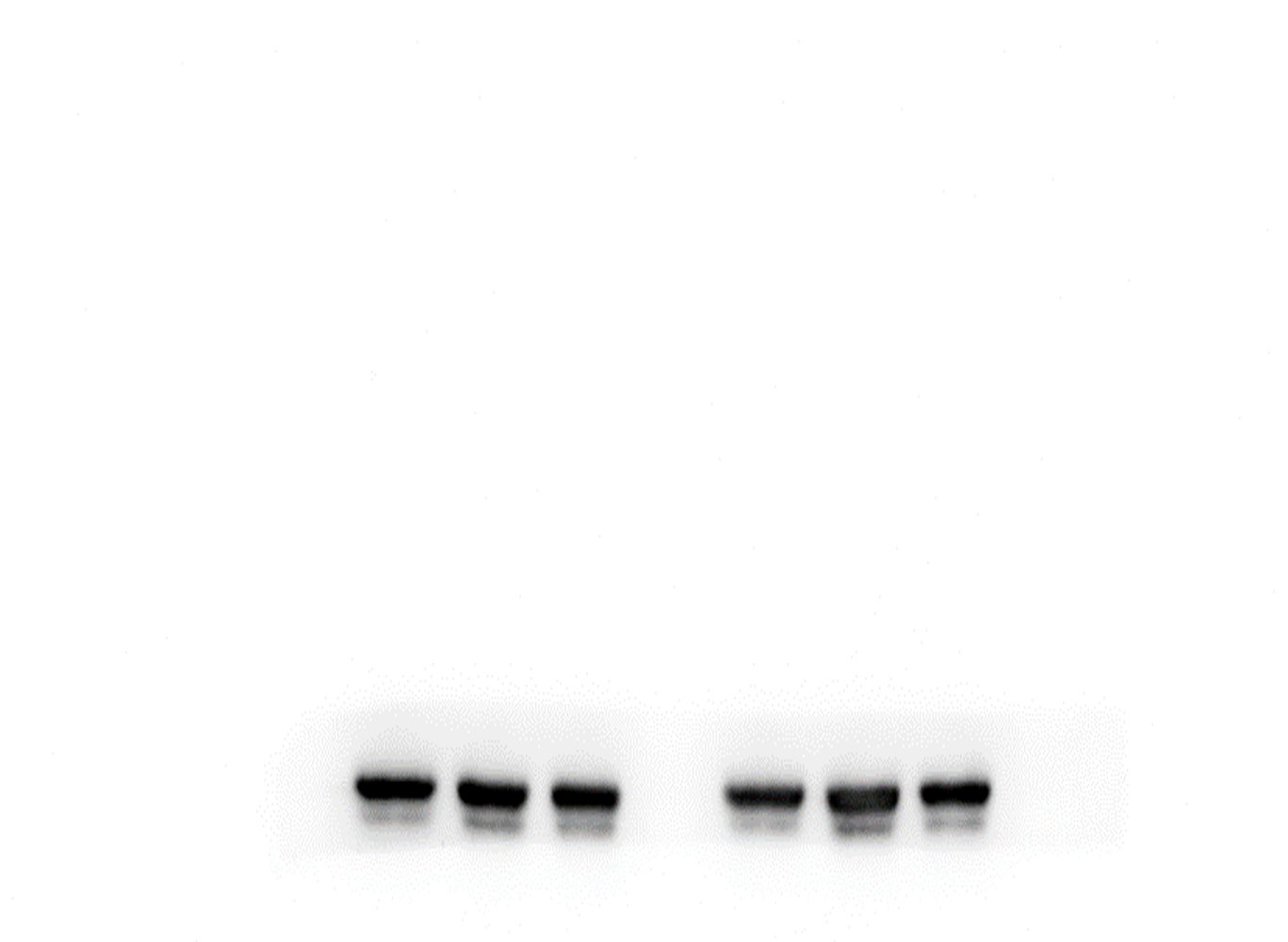

Supplement: Figure 6—source data 2. — Hippocampal tissues and primary hippocampal neurons were run on the same membrane. A: lanes 1-4: hippocampus from WT mice; lanes 5-8: hippocampus from KAL-TG mice; E–H: lanes 1: primary hippocampal neurons infected with GFP adenovirnus, lanes 2: primary hippocampal neurons infected with overexpressing Kallistatin adenovirus; lane 3: primary hippocampal neurons infected with overexpressing Kallistatin adenovirus and siHES1. [file elife-99462-fig6-data2.zip › Fig.6H Actin2 Neuron.tif]

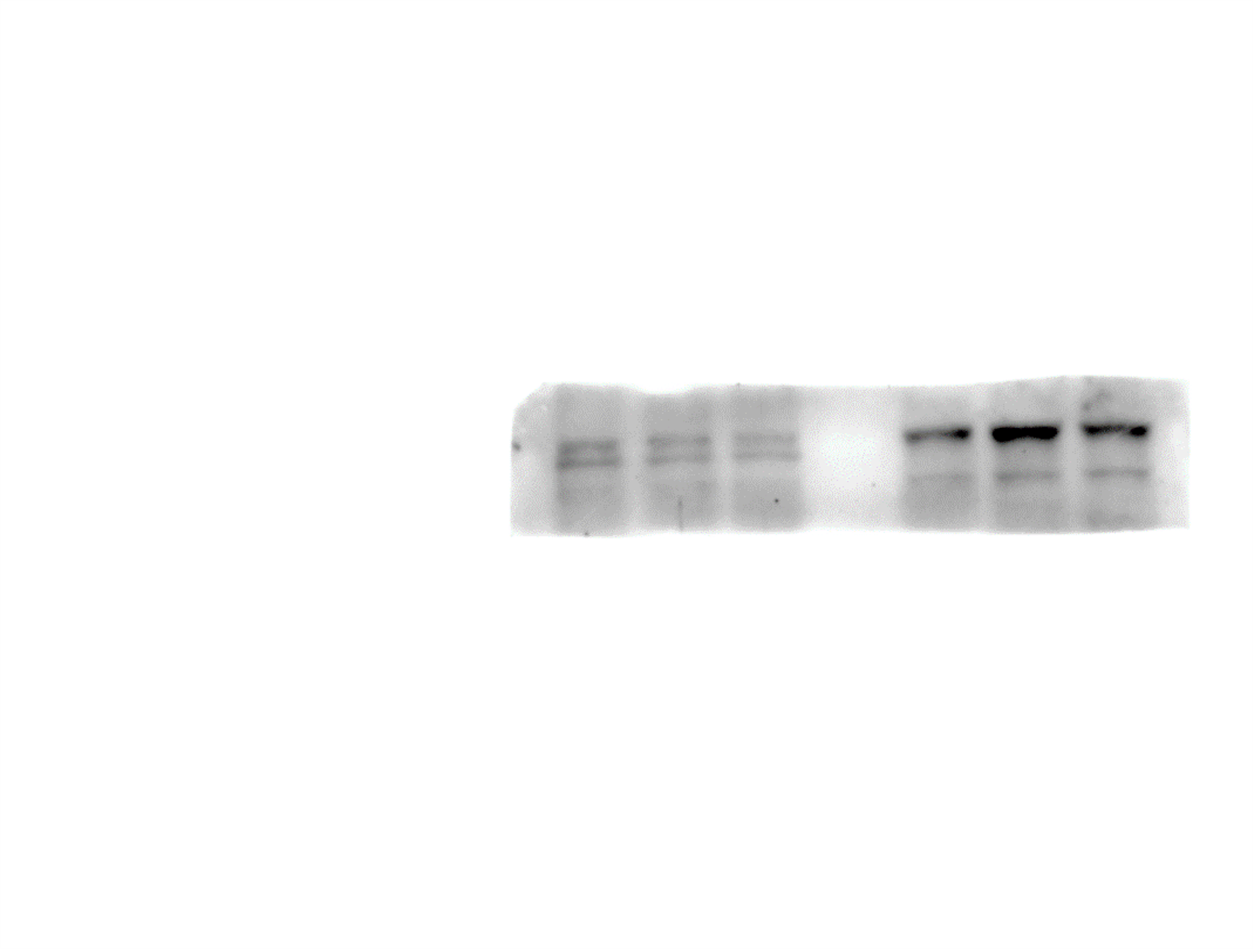

Supplement: Figure 6—source data 2. — Hippocampal tissues and primary hippocampal neurons were run on the same membrane. A: lanes 1-4: hippocampus from WT mice; lanes 5-8: hippocampus from KAL-TG mice; E–H: lanes 1: primary hippocampal neurons infected with GFP adenovirnus, lanes 2: primary hippocampal neurons infected with overexpressing Kallistatin adenovirus; lane 3: primary hippocampal neurons infected with overexpressing Kallistatin adenovirus and siHES1. [file elife-99462-fig6-data2.zip › Fig.6H BACE1 Neuron.tif]

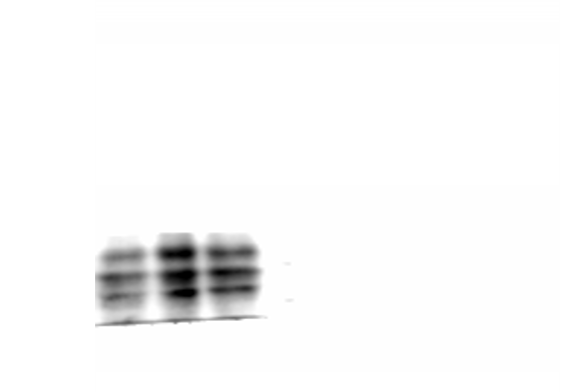

Supplement: Figure 6—source data 2. — Hippocampal tissues and primary hippocampal neurons were run on the same membrane. A: lanes 1-4: hippocampus from WT mice; lanes 5-8: hippocampus from KAL-TG mice; E–H: lanes 1: primary hippocampal neurons infected with GFP adenovirnus, lanes 2: primary hippocampal neurons infected with overexpressing Kallistatin adenovirus; lane 3: primary hippocampal neurons infected with overexpressing Kallistatin adenovirus and siHES1. [file elife-99462-fig6-data2.zip › Fig.6H HES1 Neuron.tif]

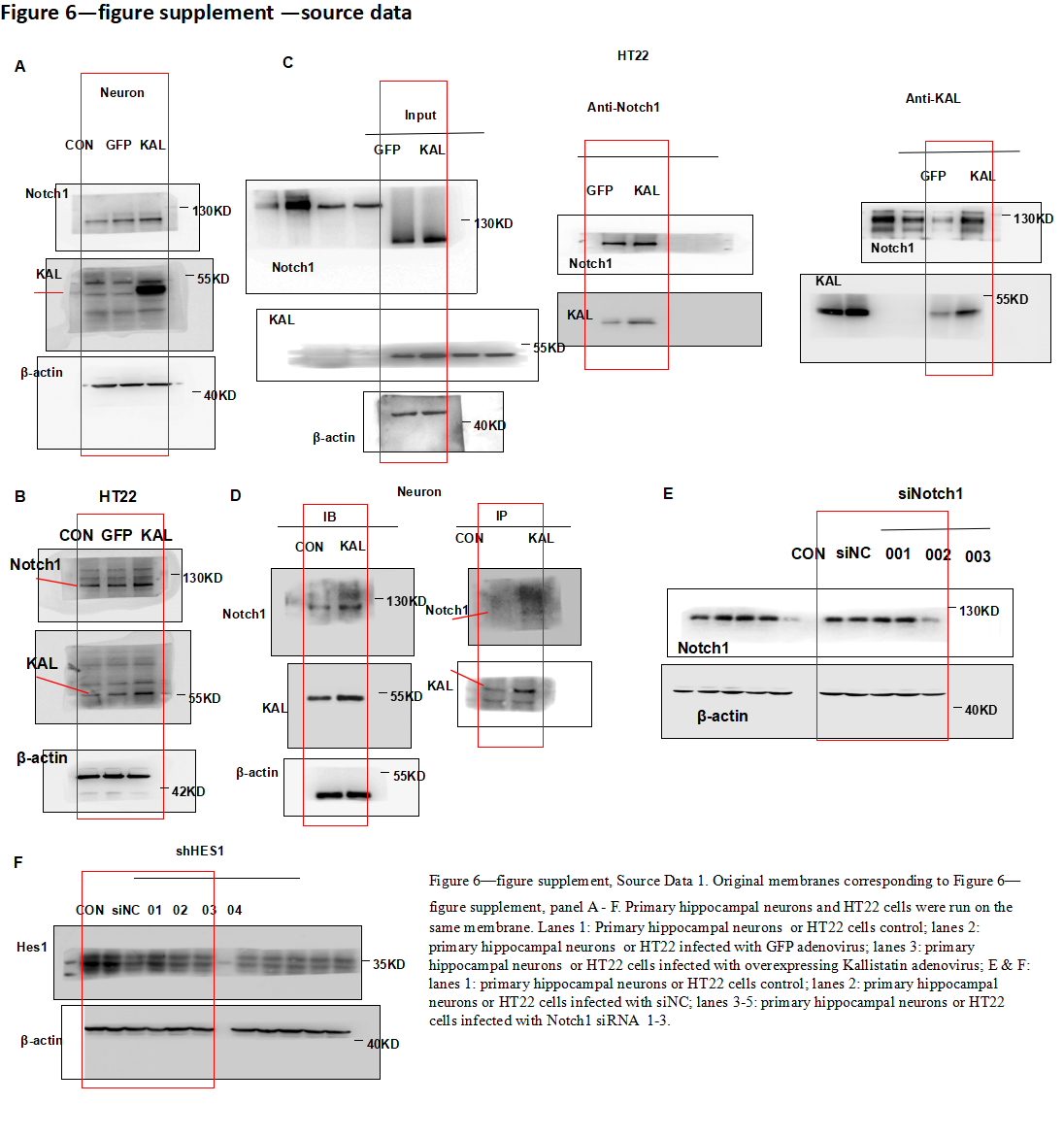

Supplement: Figure 6—figure supplement 1—source data 1. — Primary hippocampal neurons and HT22 cells were run on thesame membrane. Lanes 1: Primary hippocampal neurons or HT22 cells control; lanes 2: primary hippocampal neurons or HT22 infected with GFP adenovirus, lanes 3: primary hippocampal neurons or HT22 cells infected with overexpressing Kallistatin adenovirus, E, F: lanes 1: primary hippocampal neurons or HT22 cells control: lanes 2: primary hippocampal neurons or HT22 cells infected with siNC: lanes 3-5: primary hippocampal neurons or HT22 cells infected with Notchl siRNA 1-3. [file elife-99462-fig6-figsupp1-data1.zip › Figure 6-figure supplement-source data 1/Figure 6-figure supplement-source data.png]

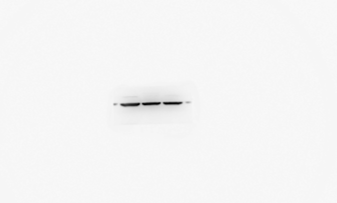

Supplement: Figure 6—figure supplement 1—source data 2. — Primary hippocampal neurons and HT22 cells were run on thesame membrane. Lanes 1: Primary hippocampal neurons or HT22 cells control; lanes 2: primary hippocampal neurons or HT22 infected with GFP adenovirus, lanes 3: primary hippocampal neurons or HT22 cells infected with overexpressing Kallistatin adenovirus, E, F: lanes 1: primary hippocampal neurons or HT22 cells control: lanes 2: primary hippocampal neurons or HT22 cells infected with siNC: lanes 3-5: primary hippocampal neurons or HT22 cells infected with Notchl siRNA 1-3. [file elife-99462-fig6-figsupp1-data2.zip › Fig.S6A Actin Neuron.tif]

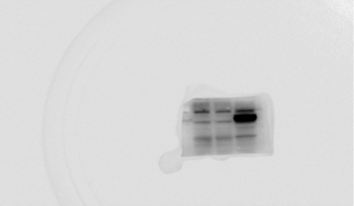

Supplement: Figure 6—figure supplement 1—source data 2. — Primary hippocampal neurons and HT22 cells were run on thesame membrane. Lanes 1: Primary hippocampal neurons or HT22 cells control; lanes 2: primary hippocampal neurons or HT22 infected with GFP adenovirus, lanes 3: primary hippocampal neurons or HT22 cells infected with overexpressing Kallistatin adenovirus, E, F: lanes 1: primary hippocampal neurons or HT22 cells control: lanes 2: primary hippocampal neurons or HT22 cells infected with siNC: lanes 3-5: primary hippocampal neurons or HT22 cells infected with Notchl siRNA 1-3. [file elife-99462-fig6-figsupp1-data2.zip › Fig.S6A KAL Neuron.tif]

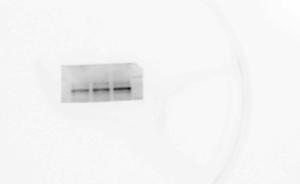

Supplement: Figure 6—figure supplement 1—source data 2. — Primary hippocampal neurons and HT22 cells were run on thesame membrane. Lanes 1: Primary hippocampal neurons or HT22 cells control; lanes 2: primary hippocampal neurons or HT22 infected with GFP adenovirus, lanes 3: primary hippocampal neurons or HT22 cells infected with overexpressing Kallistatin adenovirus, E, F: lanes 1: primary hippocampal neurons or HT22 cells control: lanes 2: primary hippocampal neurons or HT22 cells infected with siNC: lanes 3-5: primary hippocampal neurons or HT22 cells infected with Notchl siRNA 1-3. [file elife-99462-fig6-figsupp1-data2.zip › Fig.S6A Notch1 Neuron.tif]

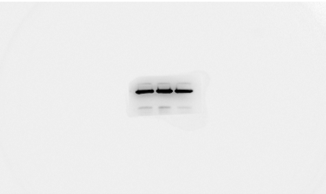

Supplement: Figure 6—figure supplement 1—source data 2. — Primary hippocampal neurons and HT22 cells were run on thesame membrane. Lanes 1: Primary hippocampal neurons or HT22 cells control; lanes 2: primary hippocampal neurons or HT22 infected with GFP adenovirus, lanes 3: primary hippocampal neurons or HT22 cells infected with overexpressing Kallistatin adenovirus, E, F: lanes 1: primary hippocampal neurons or HT22 cells control: lanes 2: primary hippocampal neurons or HT22 cells infected with siNC: lanes 3-5: primary hippocampal neurons or HT22 cells infected with Notchl siRNA 1-3. [file elife-99462-fig6-figsupp1-data2.zip › Fig.S6B Actin HT22.tif]

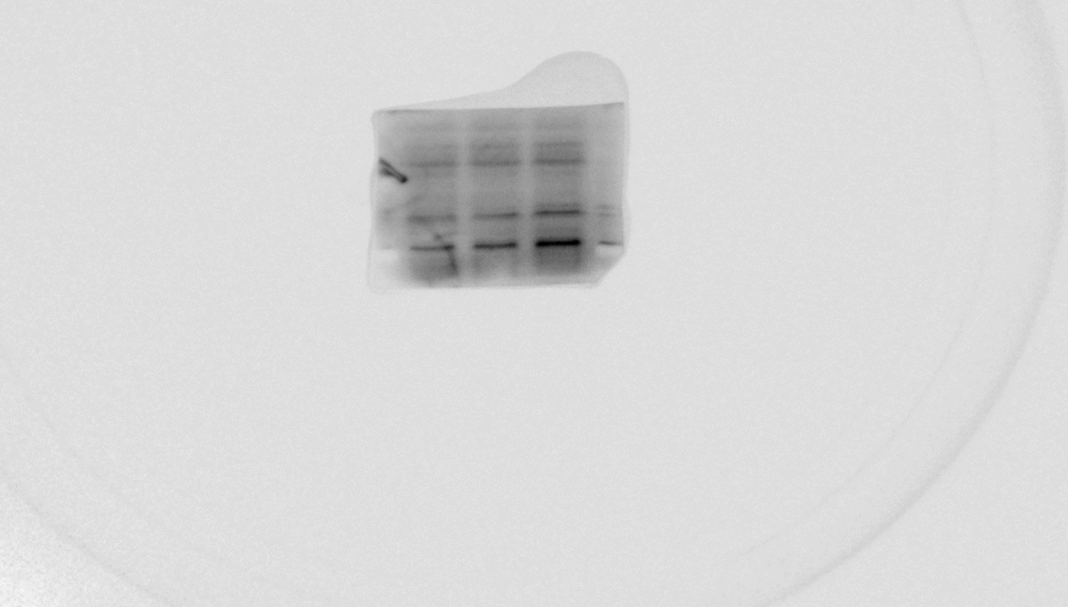

Supplement: Figure 6—figure supplement 1—source data 2. — Primary hippocampal neurons and HT22 cells were run on thesame membrane. Lanes 1: Primary hippocampal neurons or HT22 cells control; lanes 2: primary hippocampal neurons or HT22 infected with GFP adenovirus, lanes 3: primary hippocampal neurons or HT22 cells infected with overexpressing Kallistatin adenovirus, E, F: lanes 1: primary hippocampal neurons or HT22 cells control: lanes 2: primary hippocampal neurons or HT22 cells infected with siNC: lanes 3-5: primary hippocampal neurons or HT22 cells infected with Notchl siRNA 1-3. [file elife-99462-fig6-figsupp1-data2.zip › Fig.S6B KAL HT22.tif]

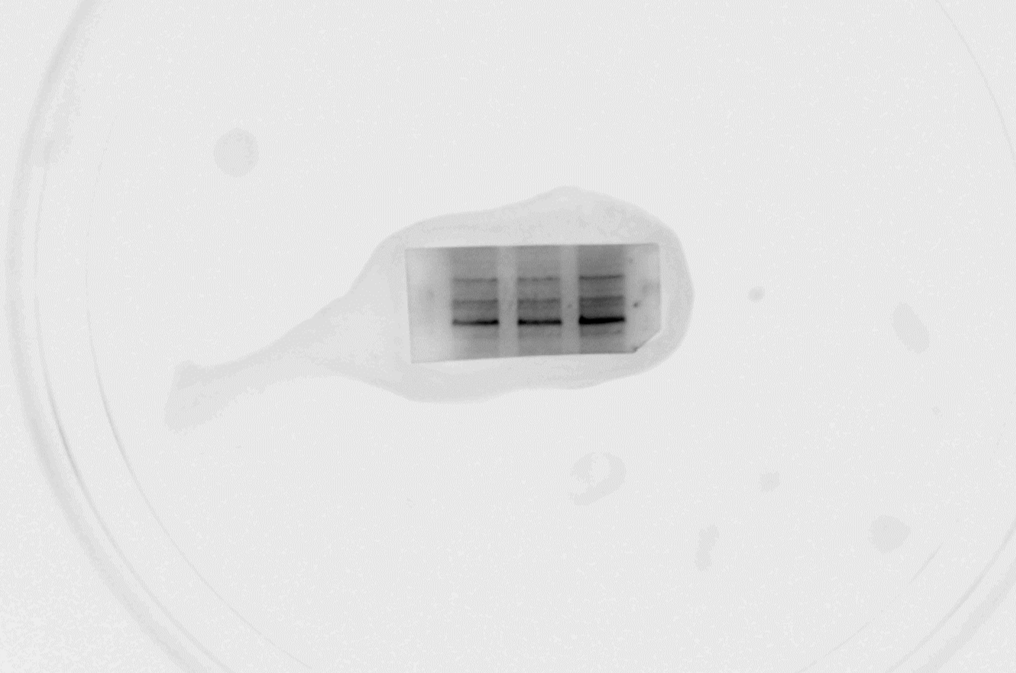

Supplement: Figure 6—figure supplement 1—source data 2. — Primary hippocampal neurons and HT22 cells were run on thesame membrane. Lanes 1: Primary hippocampal neurons or HT22 cells control; lanes 2: primary hippocampal neurons or HT22 infected with GFP adenovirus, lanes 3: primary hippocampal neurons or HT22 cells infected with overexpressing Kallistatin adenovirus, E, F: lanes 1: primary hippocampal neurons or HT22 cells control: lanes 2: primary hippocampal neurons or HT22 cells infected with siNC: lanes 3-5: primary hippocampal neurons or HT22 cells infected with Notchl siRNA 1-3. [file elife-99462-fig6-figsupp1-data2.zip › Fig.S6B Notch1 HT22.tif]

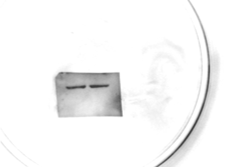

Supplement: Figure 6—figure supplement 1—source data 2. — Primary hippocampal neurons and HT22 cells were run on thesame membrane. Lanes 1: Primary hippocampal neurons or HT22 cells control; lanes 2: primary hippocampal neurons or HT22 infected with GFP adenovirus, lanes 3: primary hippocampal neurons or HT22 cells infected with overexpressing Kallistatin adenovirus, E, F: lanes 1: primary hippocampal neurons or HT22 cells control: lanes 2: primary hippocampal neurons or HT22 cells infected with siNC: lanes 3-5: primary hippocampal neurons or HT22 cells infected with Notchl siRNA 1-3. [file elife-99462-fig6-figsupp1-data2.zip › Fig.S6C Actin input HT22.tif]

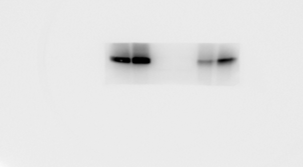

Supplement: Figure 6—figure supplement 1—source data 2. — Primary hippocampal neurons and HT22 cells were run on thesame membrane. Lanes 1: Primary hippocampal neurons or HT22 cells control; lanes 2: primary hippocampal neurons or HT22 infected with GFP adenovirus, lanes 3: primary hippocampal neurons or HT22 cells infected with overexpressing Kallistatin adenovirus, E, F: lanes 1: primary hippocampal neurons or HT22 cells control: lanes 2: primary hippocampal neurons or HT22 cells infected with siNC: lanes 3-5: primary hippocampal neurons or HT22 cells infected with Notchl siRNA 1-3. [file elife-99462-fig6-figsupp1-data2.zip › Fig.S6C KAL anti KAL HT22.tif]

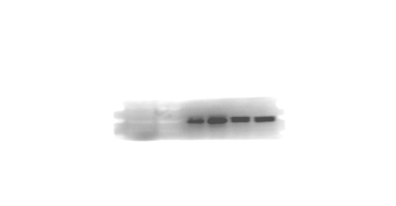

Supplement: Figure 6—figure supplement 1—source data 2. — Primary hippocampal neurons and HT22 cells were run on thesame membrane. Lanes 1: Primary hippocampal neurons or HT22 cells control; lanes 2: primary hippocampal neurons or HT22 infected with GFP adenovirus, lanes 3: primary hippocampal neurons or HT22 cells infected with overexpressing Kallistatin adenovirus, E, F: lanes 1: primary hippocampal neurons or HT22 cells control: lanes 2: primary hippocampal neurons or HT22 cells infected with siNC: lanes 3-5: primary hippocampal neurons or HT22 cells infected with Notchl siRNA 1-3. [file elife-99462-fig6-figsupp1-data2.zip › Fig.S6C KAL input HT22.tif]

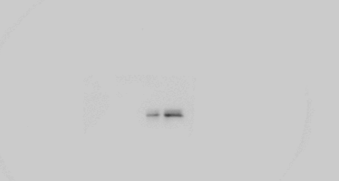

Supplement: Figure 6—figure supplement 1—source data 2. — Primary hippocampal neurons and HT22 cells were run on thesame membrane. Lanes 1: Primary hippocampal neurons or HT22 cells control; lanes 2: primary hippocampal neurons or HT22 infected with GFP adenovirus, lanes 3: primary hippocampal neurons or HT22 cells infected with overexpressing Kallistatin adenovirus, E, F: lanes 1: primary hippocampal neurons or HT22 cells control: lanes 2: primary hippocampal neurons or HT22 cells infected with siNC: lanes 3-5: primary hippocampal neurons or HT22 cells infected with Notchl siRNA 1-3. [file elife-99462-fig6-figsupp1-data2.zip › Fig.S6C Notch1 KAL anti notch HT22.tif]

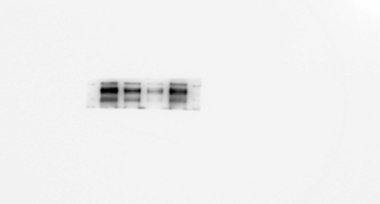

Supplement: Figure 6—figure supplement 1—source data 2. — Primary hippocampal neurons and HT22 cells were run on thesame membrane. Lanes 1: Primary hippocampal neurons or HT22 cells control; lanes 2: primary hippocampal neurons or HT22 infected with GFP adenovirus, lanes 3: primary hippocampal neurons or HT22 cells infected with overexpressing Kallistatin adenovirus, E, F: lanes 1: primary hippocampal neurons or HT22 cells control: lanes 2: primary hippocampal neurons or HT22 cells infected with siNC: lanes 3-5: primary hippocampal neurons or HT22 cells infected with Notchl siRNA 1-3. [file elife-99462-fig6-figsupp1-data2.zip › Fig.S6C Notch1 anti KAL HT22.tif]

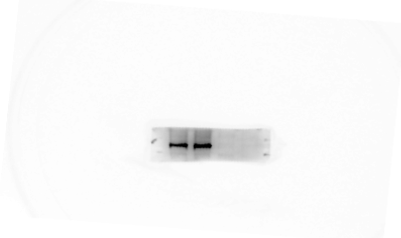

Supplement: Figure 6—figure supplement 1—source data 2. — Primary hippocampal neurons and HT22 cells were run on thesame membrane. Lanes 1: Primary hippocampal neurons or HT22 cells control; lanes 2: primary hippocampal neurons or HT22 infected with GFP adenovirus, lanes 3: primary hippocampal neurons or HT22 cells infected with overexpressing Kallistatin adenovirus, E, F: lanes 1: primary hippocampal neurons or HT22 cells control: lanes 2: primary hippocampal neurons or HT22 cells infected with siNC: lanes 3-5: primary hippocampal neurons or HT22 cells infected with Notchl siRNA 1-3. [file elife-99462-fig6-figsupp1-data2.zip › Fig.S6C Notch1 anti notch HT22.tif]

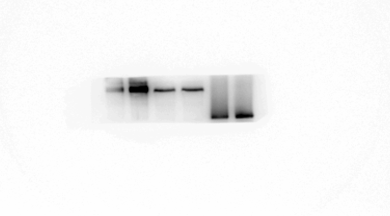

Supplement: Figure 6—figure supplement 1—source data 2. — Primary hippocampal neurons and HT22 cells were run on thesame membrane. Lanes 1: Primary hippocampal neurons or HT22 cells control; lanes 2: primary hippocampal neurons or HT22 infected with GFP adenovirus, lanes 3: primary hippocampal neurons or HT22 cells infected with overexpressing Kallistatin adenovirus, E, F: lanes 1: primary hippocampal neurons or HT22 cells control: lanes 2: primary hippocampal neurons or HT22 cells infected with siNC: lanes 3-5: primary hippocampal neurons or HT22 cells infected with Notchl siRNA 1-3. [file elife-99462-fig6-figsupp1-data2.zip › Fig.S6C Notch1 input HT22.tif]

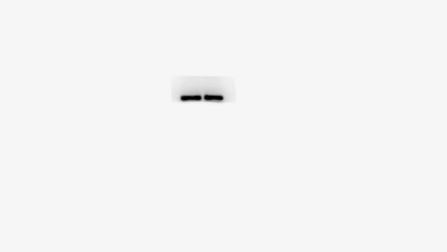

Supplement: Figure 6—figure supplement 1—source data 2. — Primary hippocampal neurons and HT22 cells were run on thesame membrane. Lanes 1: Primary hippocampal neurons or HT22 cells control; lanes 2: primary hippocampal neurons or HT22 infected with GFP adenovirus, lanes 3: primary hippocampal neurons or HT22 cells infected with overexpressing Kallistatin adenovirus, E, F: lanes 1: primary hippocampal neurons or HT22 cells control: lanes 2: primary hippocampal neurons or HT22 cells infected with siNC: lanes 3-5: primary hippocampal neurons or HT22 cells infected with Notchl siRNA 1-3. [file elife-99462-fig6-figsupp1-data2.zip › Fig.S6D Actin IB HT22.tif]

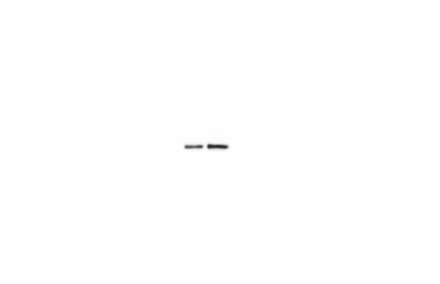

Supplement: Figure 6—figure supplement 1—source data 2. — Primary hippocampal neurons and HT22 cells were run on thesame membrane. Lanes 1: Primary hippocampal neurons or HT22 cells control; lanes 2: primary hippocampal neurons or HT22 infected with GFP adenovirus, lanes 3: primary hippocampal neurons or HT22 cells infected with overexpressing Kallistatin adenovirus, E, F: lanes 1: primary hippocampal neurons or HT22 cells control: lanes 2: primary hippocampal neurons or HT22 cells infected with siNC: lanes 3-5: primary hippocampal neurons or HT22 cells infected with Notchl siRNA 1-3. [file elife-99462-fig6-figsupp1-data2.zip › Fig.S6D KAL IB HT22.tif]

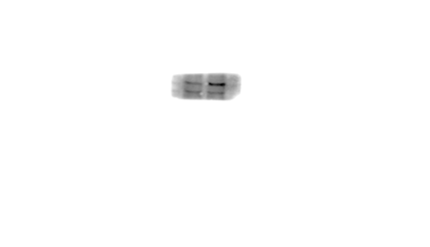

Supplement: Figure 6—figure supplement 1—source data 2. — Primary hippocampal neurons and HT22 cells were run on thesame membrane. Lanes 1: Primary hippocampal neurons or HT22 cells control; lanes 2: primary hippocampal neurons or HT22 infected with GFP adenovirus, lanes 3: primary hippocampal neurons or HT22 cells infected with overexpressing Kallistatin adenovirus, E, F: lanes 1: primary hippocampal neurons or HT22 cells control: lanes 2: primary hippocampal neurons or HT22 cells infected with siNC: lanes 3-5: primary hippocampal neurons or HT22 cells infected with Notchl siRNA 1-3. [file elife-99462-fig6-figsupp1-data2.zip › Fig.S6D KAL IP HT22.tif]

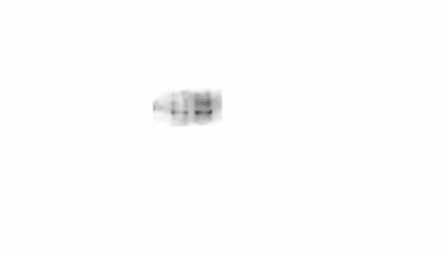

Supplement: Figure 6—figure supplement 1—source data 2. — Primary hippocampal neurons and HT22 cells were run on thesame membrane. Lanes 1: Primary hippocampal neurons or HT22 cells control; lanes 2: primary hippocampal neurons or HT22 infected with GFP adenovirus, lanes 3: primary hippocampal neurons or HT22 cells infected with overexpressing Kallistatin adenovirus, E, F: lanes 1: primary hippocampal neurons or HT22 cells control: lanes 2: primary hippocampal neurons or HT22 cells infected with siNC: lanes 3-5: primary hippocampal neurons or HT22 cells infected with Notchl siRNA 1-3. [file elife-99462-fig6-figsupp1-data2.zip › Fig.S6D Notch1 IB HT22.tif]

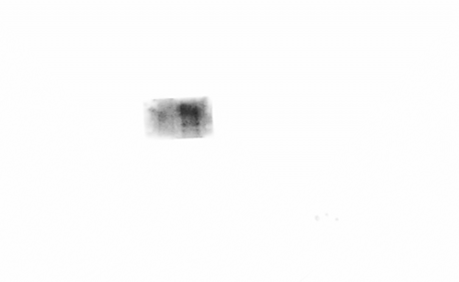

Supplement: Figure 6—figure supplement 1—source data 2. — Primary hippocampal neurons and HT22 cells were run on thesame membrane. Lanes 1: Primary hippocampal neurons or HT22 cells control; lanes 2: primary hippocampal neurons or HT22 infected with GFP adenovirus, lanes 3: primary hippocampal neurons or HT22 cells infected with overexpressing Kallistatin adenovirus, E, F: lanes 1: primary hippocampal neurons or HT22 cells control: lanes 2: primary hippocampal neurons or HT22 cells infected with siNC: lanes 3-5: primary hippocampal neurons or HT22 cells infected with Notchl siRNA 1-3. [file elife-99462-fig6-figsupp1-data2.zip › Fig.S6D Notch1 IP HT22.tif]

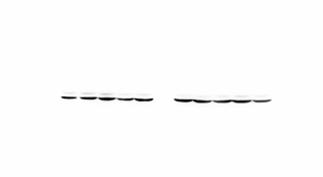

Supplement: Figure 6—figure supplement 1—source data 2. — Primary hippocampal neurons and HT22 cells were run on thesame membrane. Lanes 1: Primary hippocampal neurons or HT22 cells control; lanes 2: primary hippocampal neurons or HT22 infected with GFP adenovirus, lanes 3: primary hippocampal neurons or HT22 cells infected with overexpressing Kallistatin adenovirus, E, F: lanes 1: primary hippocampal neurons or HT22 cells control: lanes 2: primary hippocampal neurons or HT22 cells infected with siNC: lanes 3-5: primary hippocampal neurons or HT22 cells infected with Notchl siRNA 1-3. [file elife-99462-fig6-figsupp1-data2.zip › Fig.S6E Actin HT22.tif]

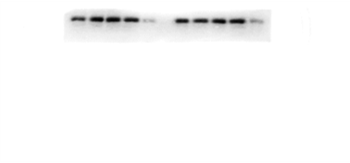

Supplement: Figure 6—figure supplement 1—source data 2. — Primary hippocampal neurons and HT22 cells were run on thesame membrane. Lanes 1: Primary hippocampal neurons or HT22 cells control; lanes 2: primary hippocampal neurons or HT22 infected with GFP adenovirus, lanes 3: primary hippocampal neurons or HT22 cells infected with overexpressing Kallistatin adenovirus, E, F: lanes 1: primary hippocampal neurons or HT22 cells control: lanes 2: primary hippocampal neurons or HT22 cells infected with siNC: lanes 3-5: primary hippocampal neurons or HT22 cells infected with Notchl siRNA 1-3. [file elife-99462-fig6-figsupp1-data2.zip › Fig.S6E NOTCH1 HT22.tif]

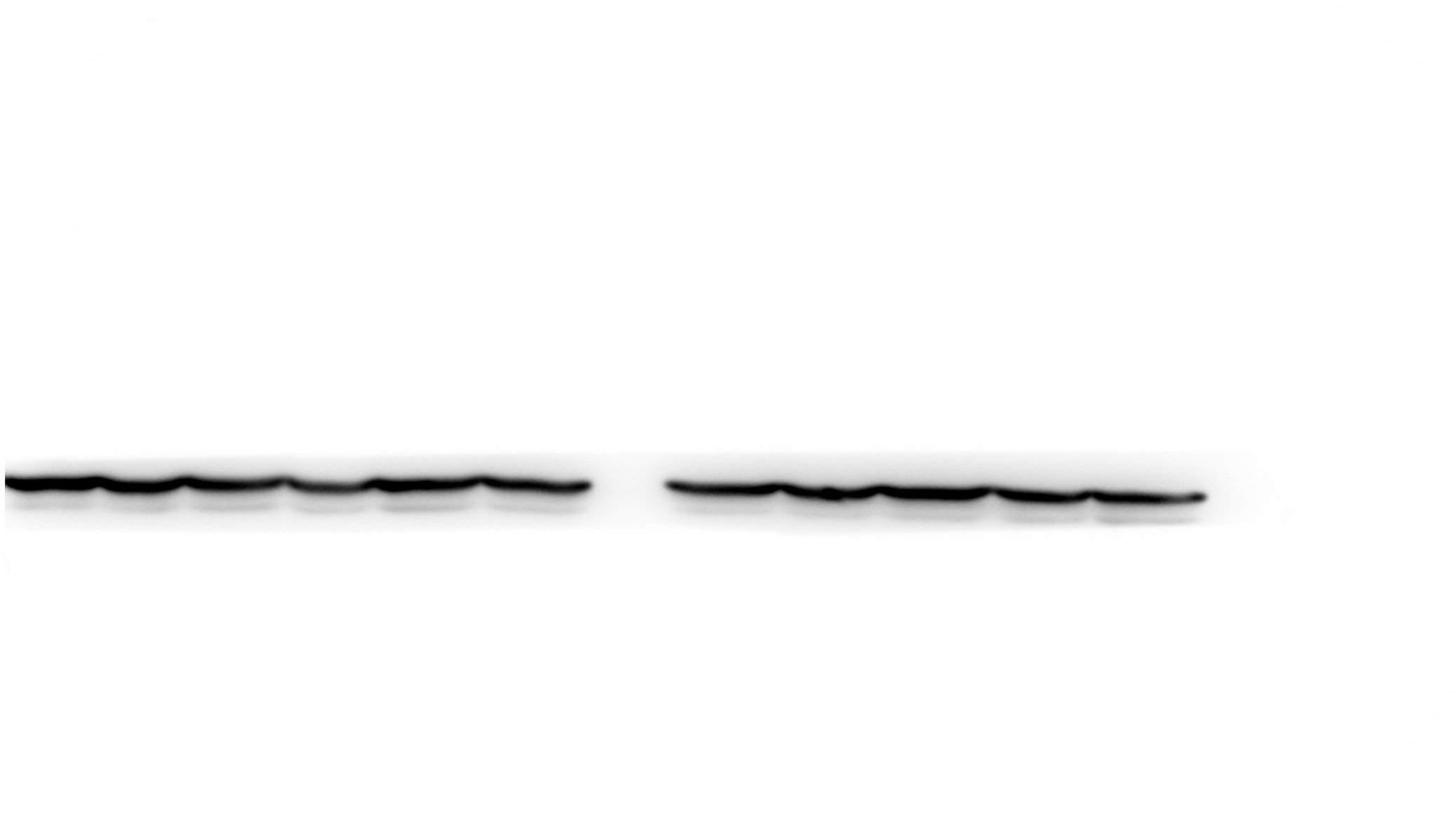

Supplement: Figure 6—figure supplement 1—source data 2. — Primary hippocampal neurons and HT22 cells were run on thesame membrane. Lanes 1: Primary hippocampal neurons or HT22 cells control; lanes 2: primary hippocampal neurons or HT22 infected with GFP adenovirus, lanes 3: primary hippocampal neurons or HT22 cells infected with overexpressing Kallistatin adenovirus, E, F: lanes 1: primary hippocampal neurons or HT22 cells control: lanes 2: primary hippocampal neurons or HT22 cells infected with siNC: lanes 3-5: primary hippocampal neurons or HT22 cells infected with Notchl siRNA 1-3. [file elife-99462-fig6-figsupp1-data2.zip › Fig.S6F Actin HT22.tif]

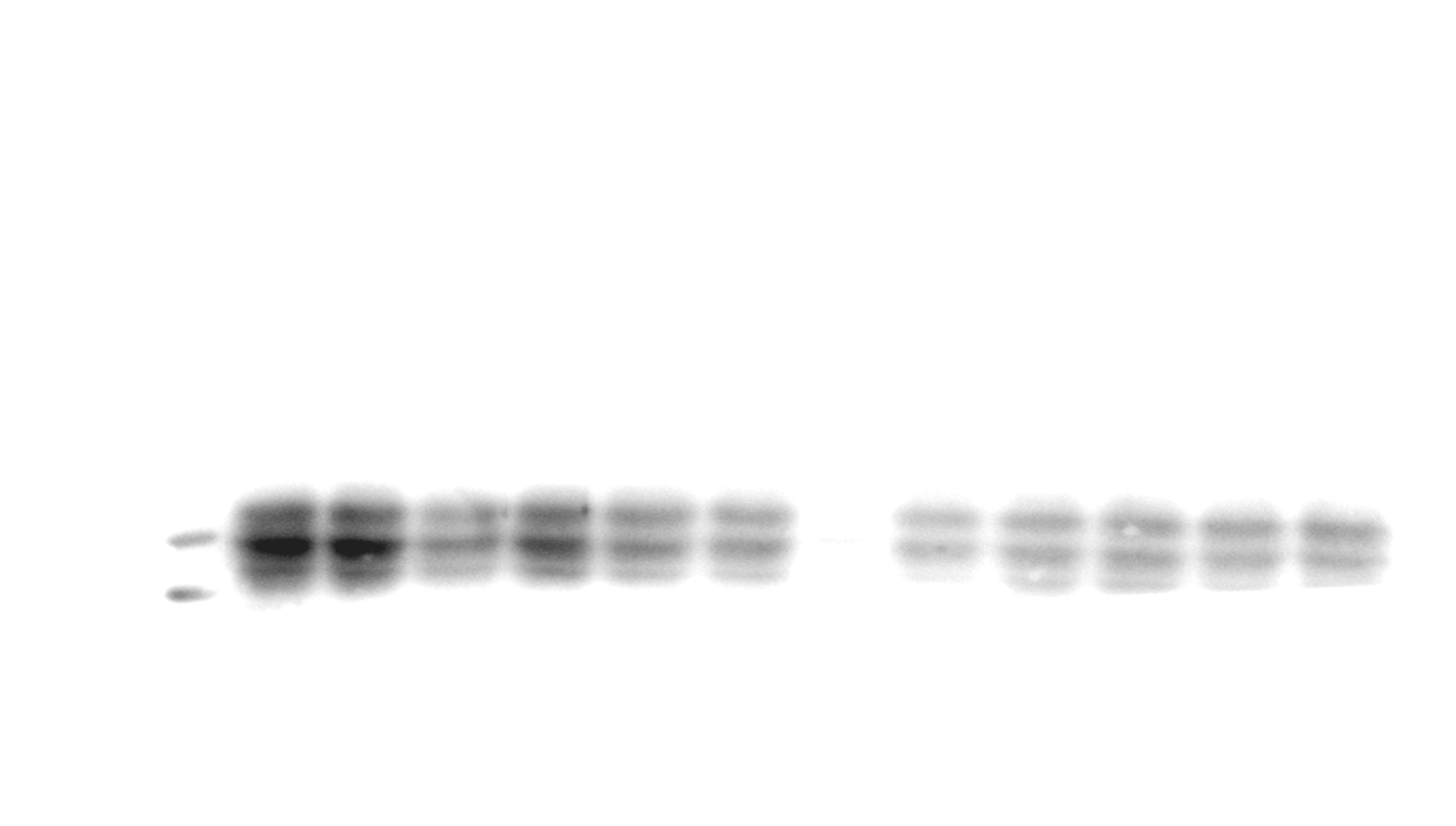

Supplement: Figure 6—figure supplement 1—source data 2. — Primary hippocampal neurons and HT22 cells were run on thesame membrane. Lanes 1: Primary hippocampal neurons or HT22 cells control; lanes 2: primary hippocampal neurons or HT22 infected with GFP adenovirus, lanes 3: primary hippocampal neurons or HT22 cells infected with overexpressing Kallistatin adenovirus, E, F: lanes 1: primary hippocampal neurons or HT22 cells control: lanes 2: primary hippocampal neurons or HT22 cells infected with siNC: lanes 3-5: primary hippocampal neurons or HT22 cells infected with Notchl siRNA 1-3. [file elife-99462-fig6-figsupp1-data2.zip › Fig.S6F HES1 HT22.tif]

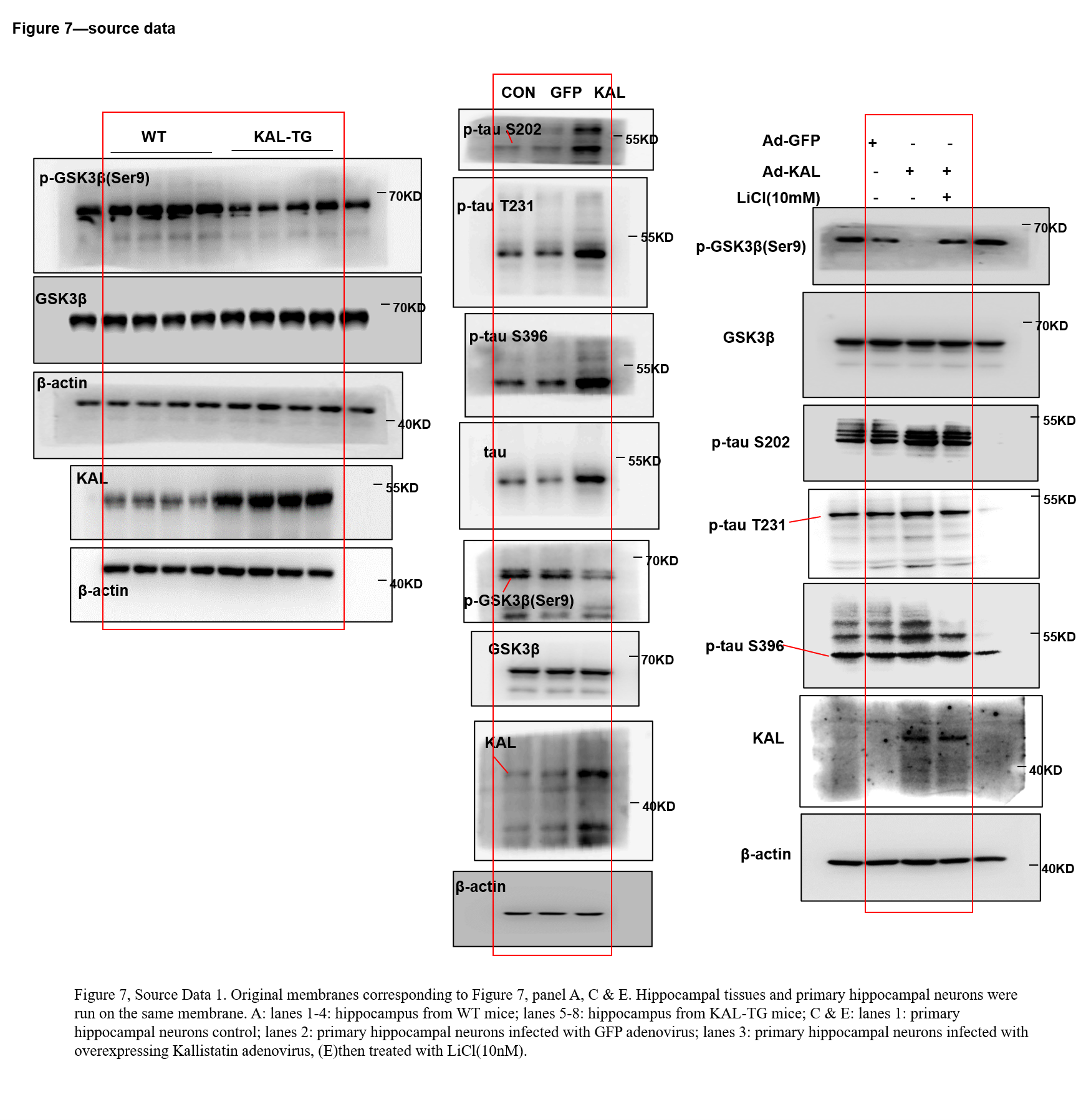

Supplement: Figure 7—source data 1. — Hippocampal tissues and primary hippocampal neurons were run on the same membrane. A: lanes 1-4: hippocampus from WT mice, lanes 5-8: hippocampus from KAL-TG mice; C, E: lanes 1: primary hippocampal neurons control; lanes 2: primary hippocampal neurons infected with GFP adenovirus; lanes 3: primary hippocampal neurons infected with overexpressing Kallistatin adenovirus, (E) then treated with LiCl (10 nM). [file elife-99462-fig7-data1.zip › Figure 7-source data 1/Figure 7-source data.png]

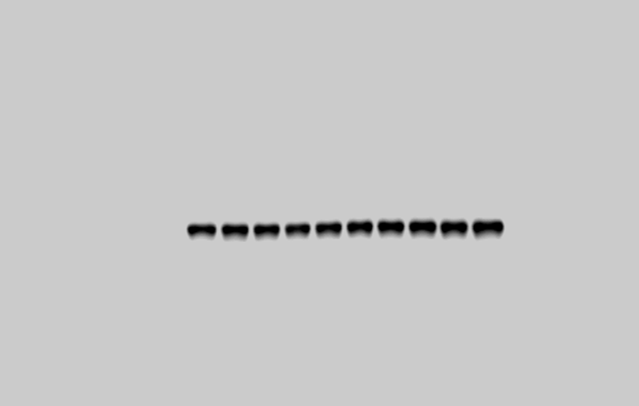

Supplement: Figure 7—source data 2. — Hippocampal tissues and primary hippocampal neurons were run on the same membrane. A: lanes 1-4: hippocampus from WT mice, lanes 5-8: hippocampus from KAL-TG mice; C, E: lanes 1: primary hippocampal neurons control; lanes 2: primary hippocampal neurons infected with GFP adenovirus; lanes 3: primary hippocampal neurons infected with overexpressing Kallistatin adenovirus, (E) then treated with LiCl (10 nM). [file elife-99462-fig7-data2.zip › Fig.7A GSK3α╕åα╕ó hippo.tif]

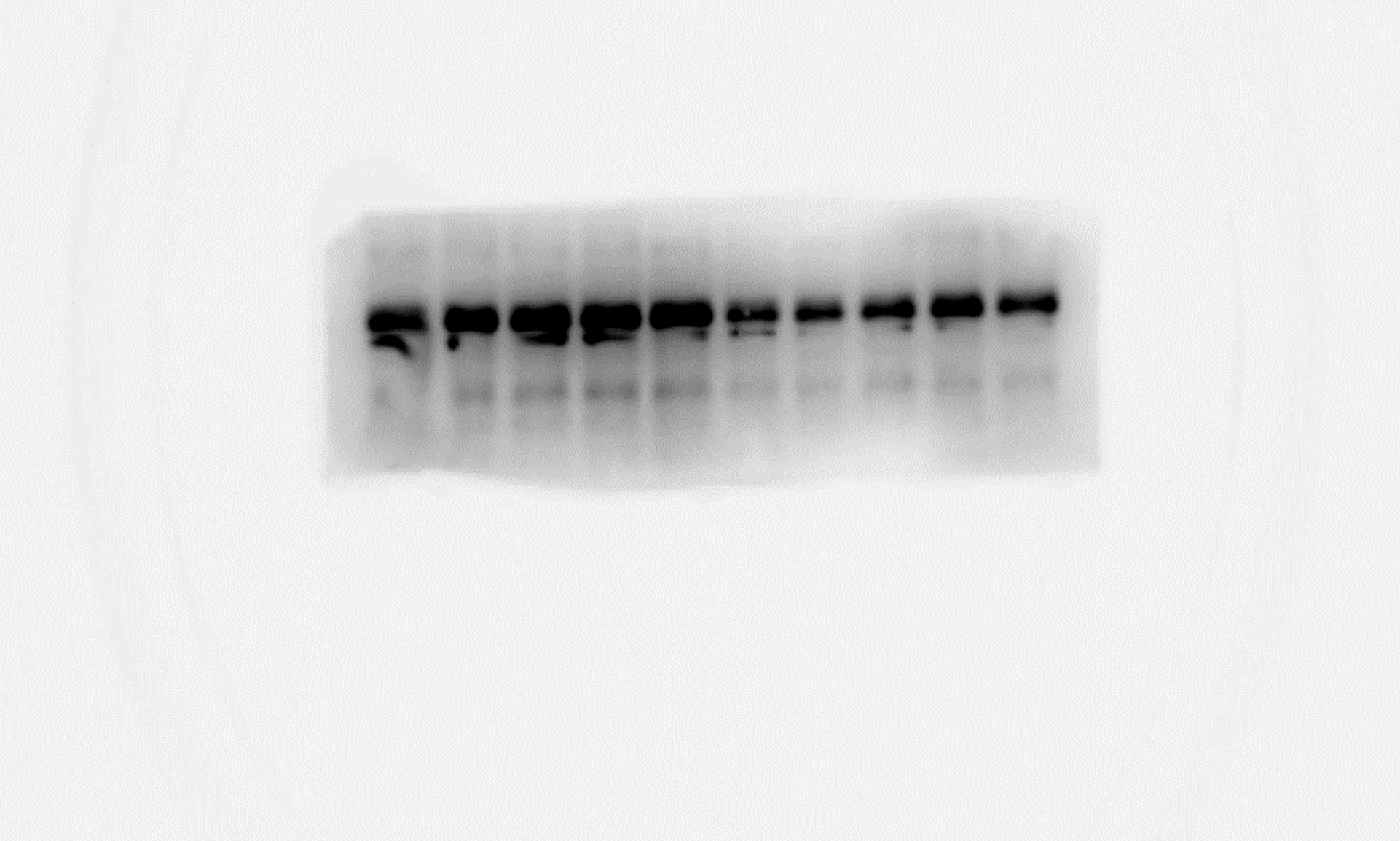

Supplement: Figure 7—source data 2. — Hippocampal tissues and primary hippocampal neurons were run on the same membrane. A: lanes 1-4: hippocampus from WT mice, lanes 5-8: hippocampus from KAL-TG mice; C, E: lanes 1: primary hippocampal neurons control; lanes 2: primary hippocampal neurons infected with GFP adenovirus; lanes 3: primary hippocampal neurons infected with overexpressing Kallistatin adenovirus, (E) then treated with LiCl (10 nM). [file elife-99462-fig7-data2.zip › Fig.7A pGSK3α╕åα╕ó(S9) hippo.tif]

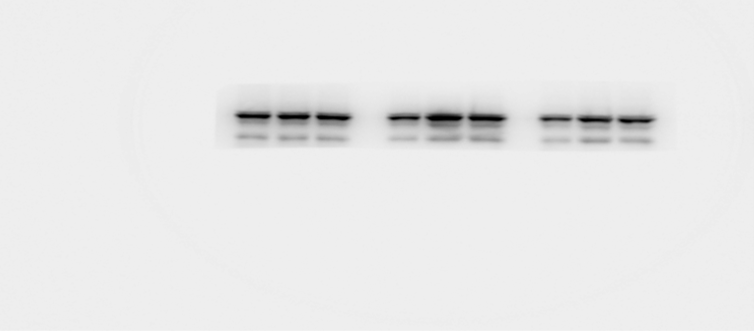

Supplement: Figure 7—source data 2. — Hippocampal tissues and primary hippocampal neurons were run on the same membrane. A: lanes 1-4: hippocampus from WT mice, lanes 5-8: hippocampus from KAL-TG mice; C, E: lanes 1: primary hippocampal neurons control; lanes 2: primary hippocampal neurons infected with GFP adenovirus; lanes 3: primary hippocampal neurons infected with overexpressing Kallistatin adenovirus, (E) then treated with LiCl (10 nM). [file elife-99462-fig7-data2.zip › Fig.7C GSK3α╕åα╕ó Neuron.tif]

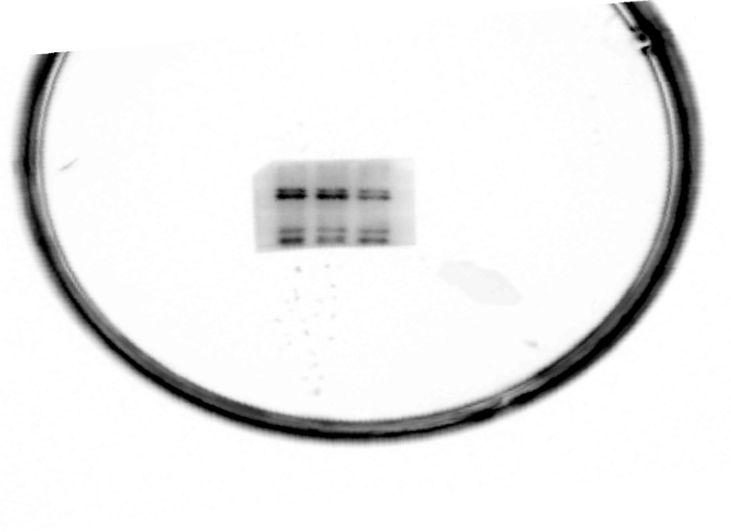

Supplement: Figure 7—source data 2. — Hippocampal tissues and primary hippocampal neurons were run on the same membrane. A: lanes 1-4: hippocampus from WT mice, lanes 5-8: hippocampus from KAL-TG mice; C, E: lanes 1: primary hippocampal neurons control; lanes 2: primary hippocampal neurons infected with GFP adenovirus; lanes 3: primary hippocampal neurons infected with overexpressing Kallistatin adenovirus, (E) then treated with LiCl (10 nM). [file elife-99462-fig7-data2.zip › Fig.7C pGSK3α╕åα╕ó(S9) Neuron.tif]

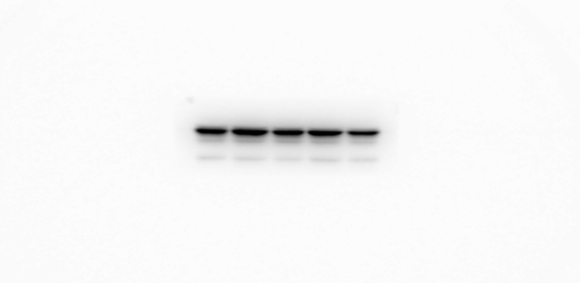

Supplement: Figure 7—source data 2. — Hippocampal tissues and primary hippocampal neurons were run on the same membrane. A: lanes 1-4: hippocampus from WT mice, lanes 5-8: hippocampus from KAL-TG mice; C, E: lanes 1: primary hippocampal neurons control; lanes 2: primary hippocampal neurons infected with GFP adenovirus; lanes 3: primary hippocampal neurons infected with overexpressing Kallistatin adenovirus, (E) then treated with LiCl (10 nM). [file elife-99462-fig7-data2.zip › Fig.7E GSK3α╕åα╕ó Neuron.tif]

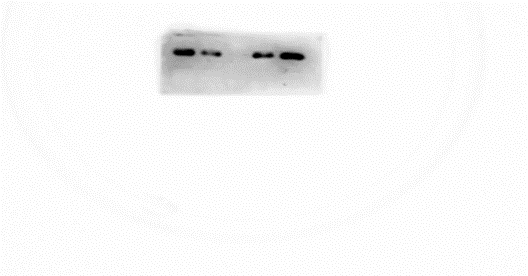

Supplement: Figure 7—source data 2. — Hippocampal tissues and primary hippocampal neurons were run on the same membrane. A: lanes 1-4: hippocampus from WT mice, lanes 5-8: hippocampus from KAL-TG mice; C, E: lanes 1: primary hippocampal neurons control; lanes 2: primary hippocampal neurons infected with GFP adenovirus; lanes 3: primary hippocampal neurons infected with overexpressing Kallistatin adenovirus, (E) then treated with LiCl (10 nM). [file elife-99462-fig7-data2.zip › Fig.7E pGSK3α╕åα╕ó(S9) Neuron.gif]

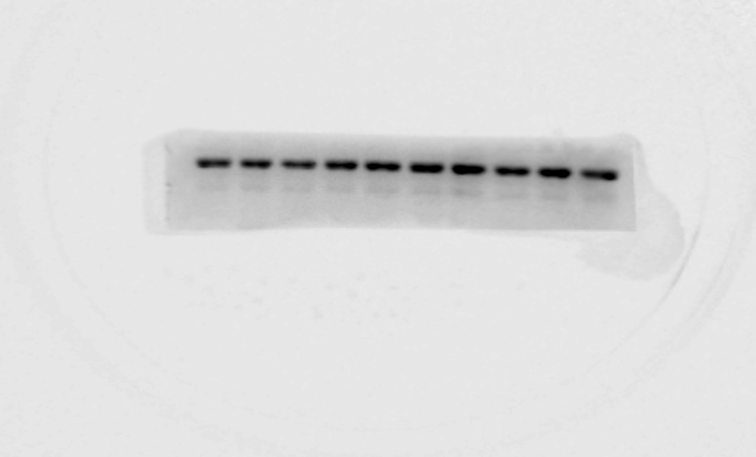

Supplement: Figure 7—source data 2. — Hippocampal tissues and primary hippocampal neurons were run on the same membrane. A: lanes 1-4: hippocampus from WT mice, lanes 5-8: hippocampus from KAL-TG mice; C, E: lanes 1: primary hippocampal neurons control; lanes 2: primary hippocampal neurons infected with GFP adenovirus; lanes 3: primary hippocampal neurons infected with overexpressing Kallistatin adenovirus, (E) then treated with LiCl (10 nM). [file elife-99462-fig7-data2.zip › Fig.7A Actin1 hippo.tif]

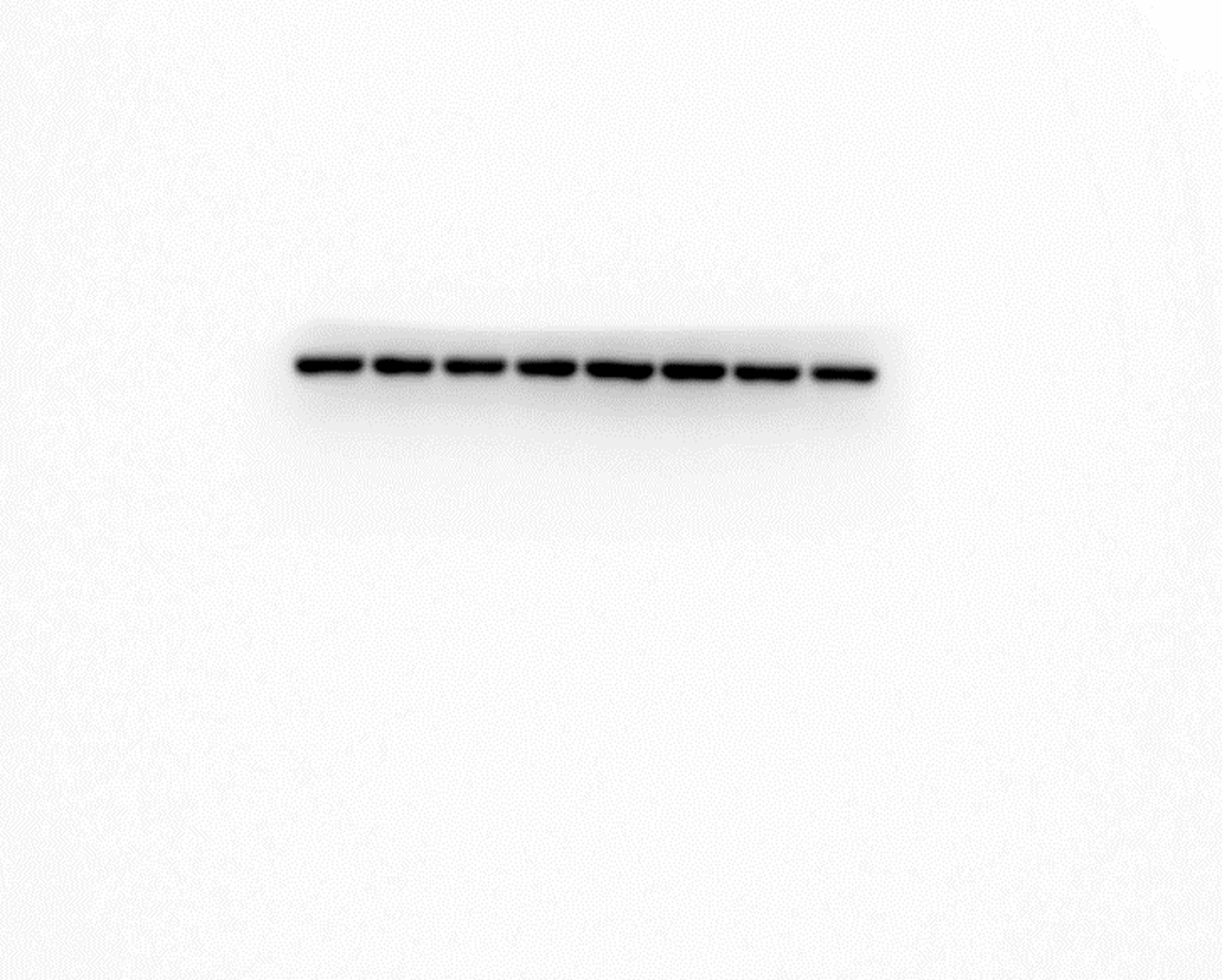

Supplement: Figure 7—source data 2. — Hippocampal tissues and primary hippocampal neurons were run on the same membrane. A: lanes 1-4: hippocampus from WT mice, lanes 5-8: hippocampus from KAL-TG mice; C, E: lanes 1: primary hippocampal neurons control; lanes 2: primary hippocampal neurons infected with GFP adenovirus; lanes 3: primary hippocampal neurons infected with overexpressing Kallistatin adenovirus, (E) then treated with LiCl (10 nM). [file elife-99462-fig7-data2.zip › Fig.7A Actin2 hippo.tif]

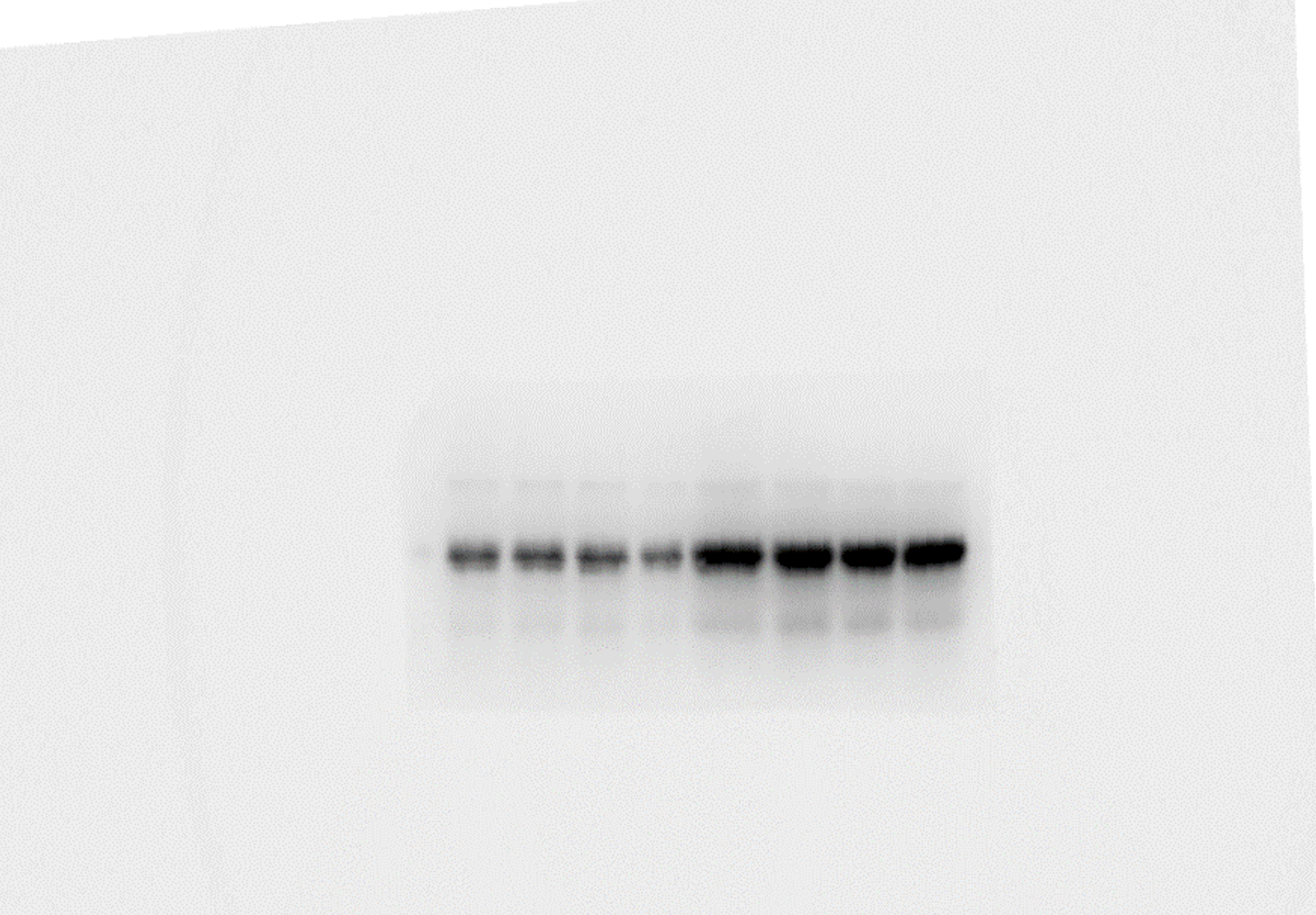

Supplement: Figure 7—source data 2. — Hippocampal tissues and primary hippocampal neurons were run on the same membrane. A: lanes 1-4: hippocampus from WT mice, lanes 5-8: hippocampus from KAL-TG mice; C, E: lanes 1: primary hippocampal neurons control; lanes 2: primary hippocampal neurons infected with GFP adenovirus; lanes 3: primary hippocampal neurons infected with overexpressing Kallistatin adenovirus, (E) then treated with LiCl (10 nM). [file elife-99462-fig7-data2.zip › Fig.7A KAL hippo.tif]

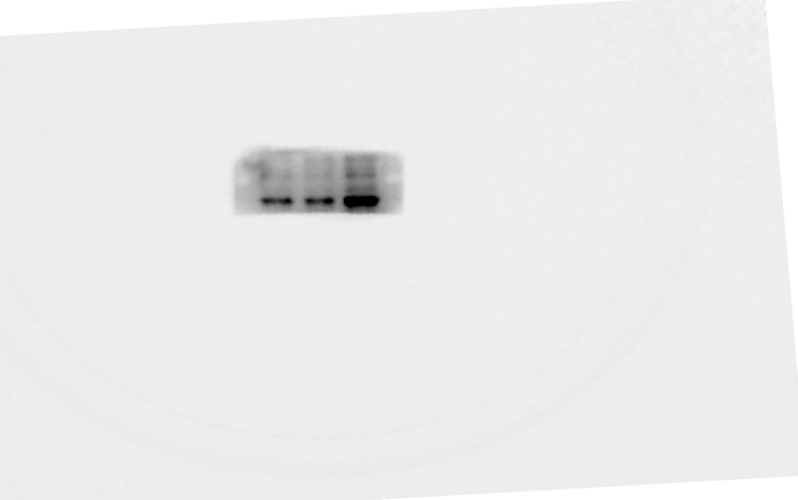

Supplement: Figure 7—source data 2. — Hippocampal tissues and primary hippocampal neurons were run on the same membrane. A: lanes 1-4: hippocampus from WT mice, lanes 5-8: hippocampus from KAL-TG mice; C, E: lanes 1: primary hippocampal neurons control; lanes 2: primary hippocampal neurons infected with GFP adenovirus; lanes 3: primary hippocampal neurons infected with overexpressing Kallistatin adenovirus, (E) then treated with LiCl (10 nM). [file elife-99462-fig7-data2.zip › Fig.7C pTau(S396) Neuron.tif]

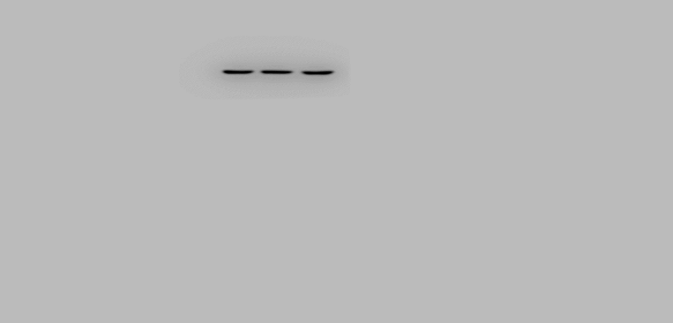

Supplement: Figure 7—source data 2. — Hippocampal tissues and primary hippocampal neurons were run on the same membrane. A: lanes 1-4: hippocampus from WT mice, lanes 5-8: hippocampus from KAL-TG mice; C, E: lanes 1: primary hippocampal neurons control; lanes 2: primary hippocampal neurons infected with GFP adenovirus; lanes 3: primary hippocampal neurons infected with overexpressing Kallistatin adenovirus, (E) then treated with LiCl (10 nM). [file elife-99462-fig7-data2.zip › Fig.7C Actin neuron.tif]

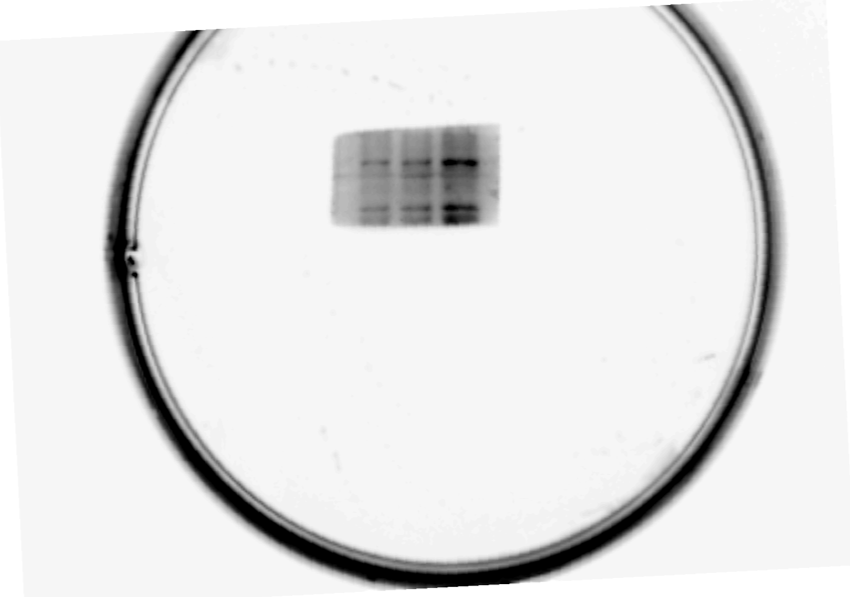

Supplement: Figure 7—source data 2. — Hippocampal tissues and primary hippocampal neurons were run on the same membrane. A: lanes 1-4: hippocampus from WT mice, lanes 5-8: hippocampus from KAL-TG mice; C, E: lanes 1: primary hippocampal neurons control; lanes 2: primary hippocampal neurons infected with GFP adenovirus; lanes 3: primary hippocampal neurons infected with overexpressing Kallistatin adenovirus, (E) then treated with LiCl (10 nM). [file elife-99462-fig7-data2.zip › Fig.7C KAL Neuron.tif]

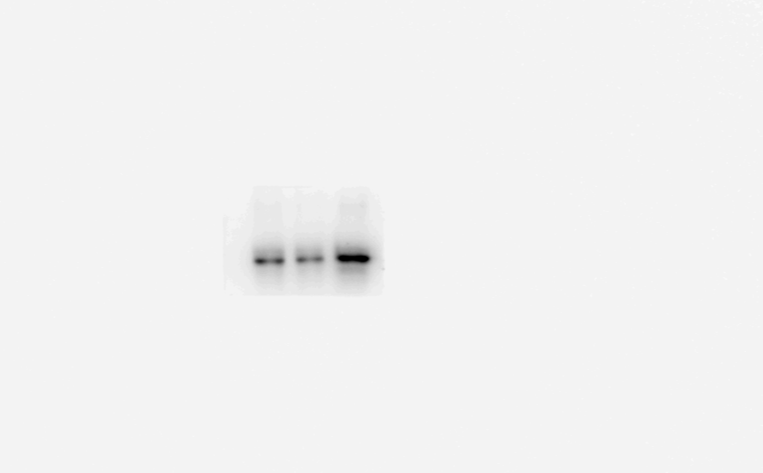

Supplement: Figure 7—source data 2. — Hippocampal tissues and primary hippocampal neurons were run on the same membrane. A: lanes 1-4: hippocampus from WT mice, lanes 5-8: hippocampus from KAL-TG mice; C, E: lanes 1: primary hippocampal neurons control; lanes 2: primary hippocampal neurons infected with GFP adenovirus; lanes 3: primary hippocampal neurons infected with overexpressing Kallistatin adenovirus, (E) then treated with LiCl (10 nM). [file elife-99462-fig7-data2.zip › Fig.7C Tau Neuron.tif]

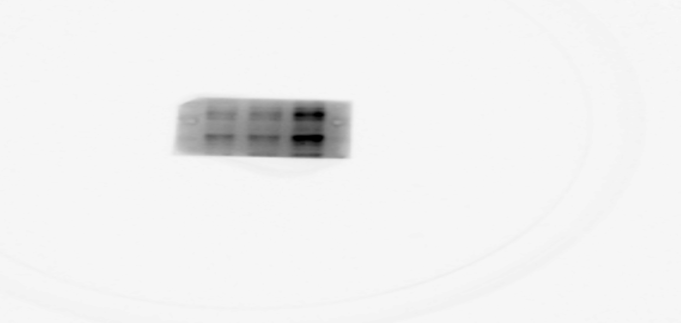

Supplement: Figure 7—source data 2. — Hippocampal tissues and primary hippocampal neurons were run on the same membrane. A: lanes 1-4: hippocampus from WT mice, lanes 5-8: hippocampus from KAL-TG mice; C, E: lanes 1: primary hippocampal neurons control; lanes 2: primary hippocampal neurons infected with GFP adenovirus; lanes 3: primary hippocampal neurons infected with overexpressing Kallistatin adenovirus, (E) then treated with LiCl (10 nM). [file elife-99462-fig7-data2.zip › Fig.7C pTau(S202) Neuron.tif]

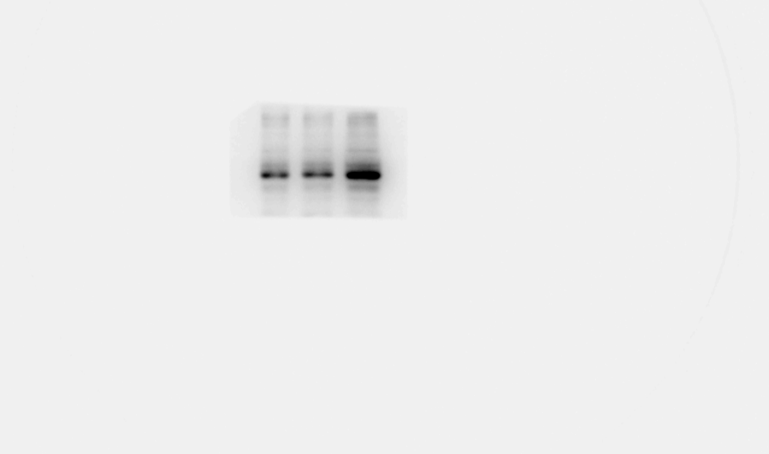

Supplement: Figure 7—source data 2. — Hippocampal tissues and primary hippocampal neurons were run on the same membrane. A: lanes 1-4: hippocampus from WT mice, lanes 5-8: hippocampus from KAL-TG mice; C, E: lanes 1: primary hippocampal neurons control; lanes 2: primary hippocampal neurons infected with GFP adenovirus; lanes 3: primary hippocampal neurons infected with overexpressing Kallistatin adenovirus, (E) then treated with LiCl (10 nM). [file elife-99462-fig7-data2.zip › Fig.7C pTau(T231) Neuron.tif]

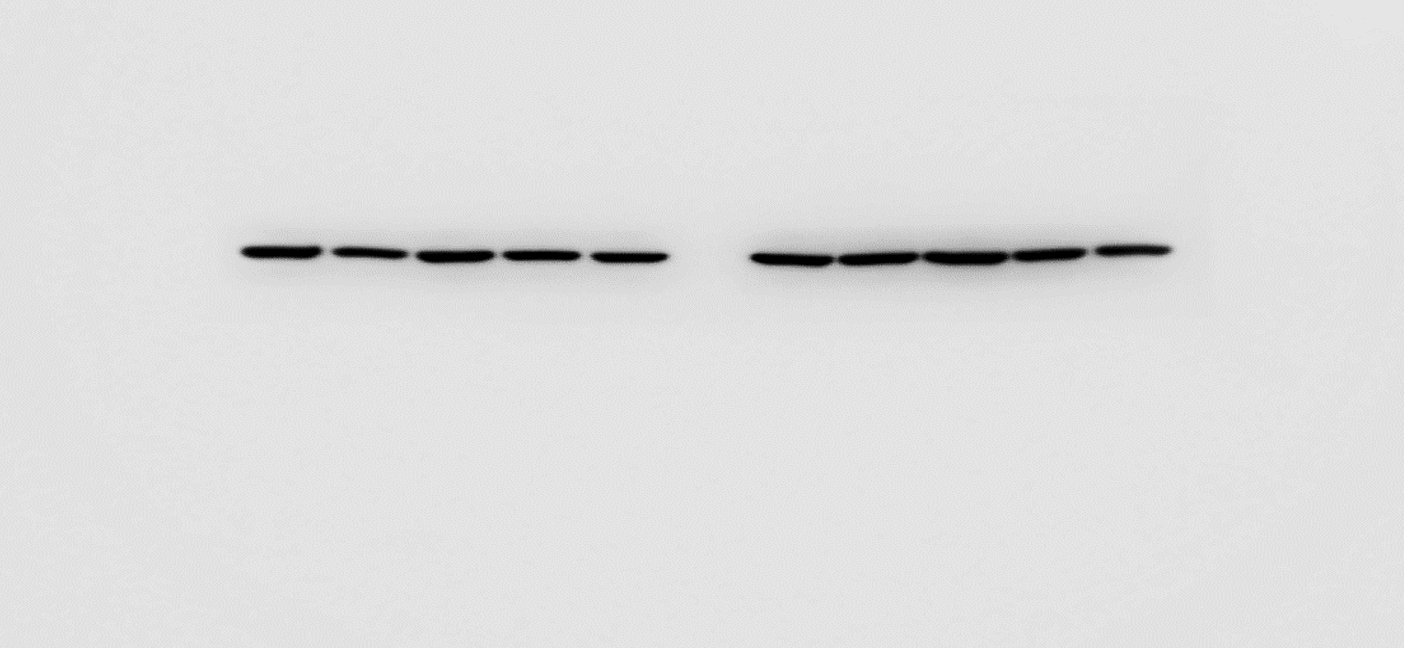

Supplement: Figure 7—source data 2. — Hippocampal tissues and primary hippocampal neurons were run on the same membrane. A: lanes 1-4: hippocampus from WT mice, lanes 5-8: hippocampus from KAL-TG mice; C, E: lanes 1: primary hippocampal neurons control; lanes 2: primary hippocampal neurons infected with GFP adenovirus; lanes 3: primary hippocampal neurons infected with overexpressing Kallistatin adenovirus, (E) then treated with LiCl (10 nM). [file elife-99462-fig7-data2.zip › Fig.7E Actin Neuron.tif]

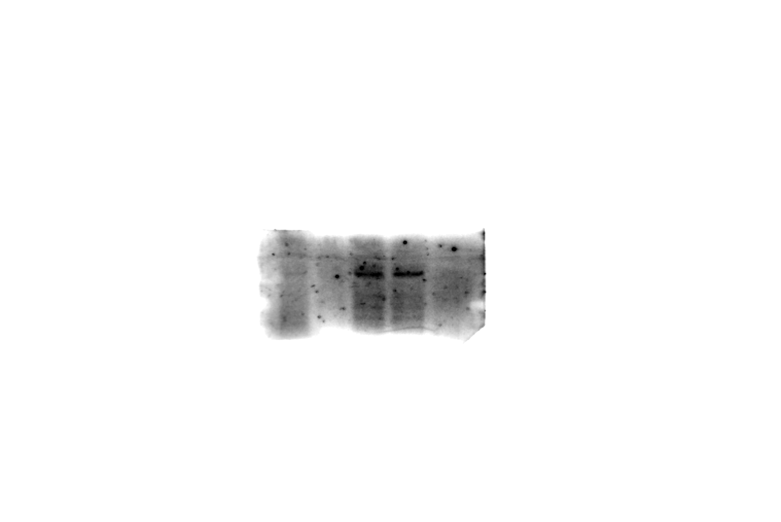

Supplement: Figure 7—source data 2. — Hippocampal tissues and primary hippocampal neurons were run on the same membrane. A: lanes 1-4: hippocampus from WT mice, lanes 5-8: hippocampus from KAL-TG mice; C, E: lanes 1: primary hippocampal neurons control; lanes 2: primary hippocampal neurons infected with GFP adenovirus; lanes 3: primary hippocampal neurons infected with overexpressing Kallistatin adenovirus, (E) then treated with LiCl (10 nM). [file elife-99462-fig7-data2.zip › Fig.7E KAL Neuron.tif]

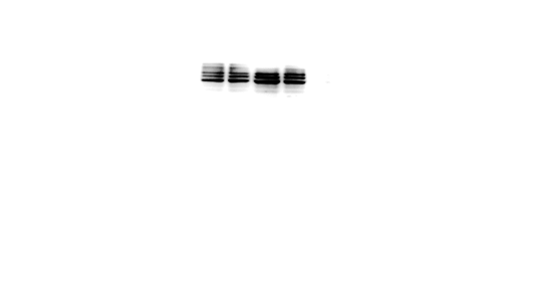

Supplement: Figure 7—source data 2. — Hippocampal tissues and primary hippocampal neurons were run on the same membrane. A: lanes 1-4: hippocampus from WT mice, lanes 5-8: hippocampus from KAL-TG mice; C, E: lanes 1: primary hippocampal neurons control; lanes 2: primary hippocampal neurons infected with GFP adenovirus; lanes 3: primary hippocampal neurons infected with overexpressing Kallistatin adenovirus, (E) then treated with LiCl (10 nM). [file elife-99462-fig7-data2.zip › Fig.7E pTau(S202) Neuron.tif]

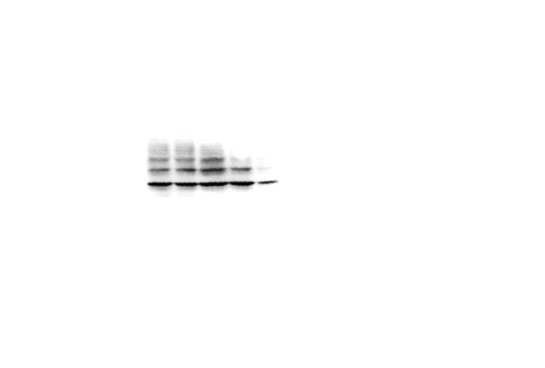

Supplement: Figure 7—source data 2. — Hippocampal tissues and primary hippocampal neurons were run on the same membrane. A: lanes 1-4: hippocampus from WT mice, lanes 5-8: hippocampus from KAL-TG mice; C, E: lanes 1: primary hippocampal neurons control; lanes 2: primary hippocampal neurons infected with GFP adenovirus; lanes 3: primary hippocampal neurons infected with overexpressing Kallistatin adenovirus, (E) then treated with LiCl (10 nM). [file elife-99462-fig7-data2.zip › Fig.7E pTau(S396) Neuron.tif]

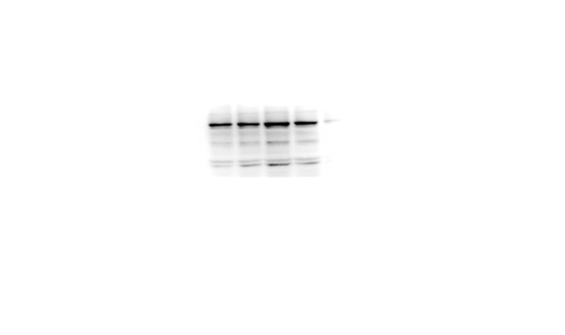

Supplement: Figure 7—source data 2. — Hippocampal tissues and primary hippocampal neurons were run on the same membrane. A: lanes 1-4: hippocampus from WT mice, lanes 5-8: hippocampus from KAL-TG mice; C, E: lanes 1: primary hippocampal neurons control; lanes 2: primary hippocampal neurons infected with GFP adenovirus; lanes 3: primary hippocampal neurons infected with overexpressing Kallistatin adenovirus, (E) then treated with LiCl (10 nM). [file elife-99462-fig7-data2.zip › Fig.7E pTau(T231) Neuron.tif]

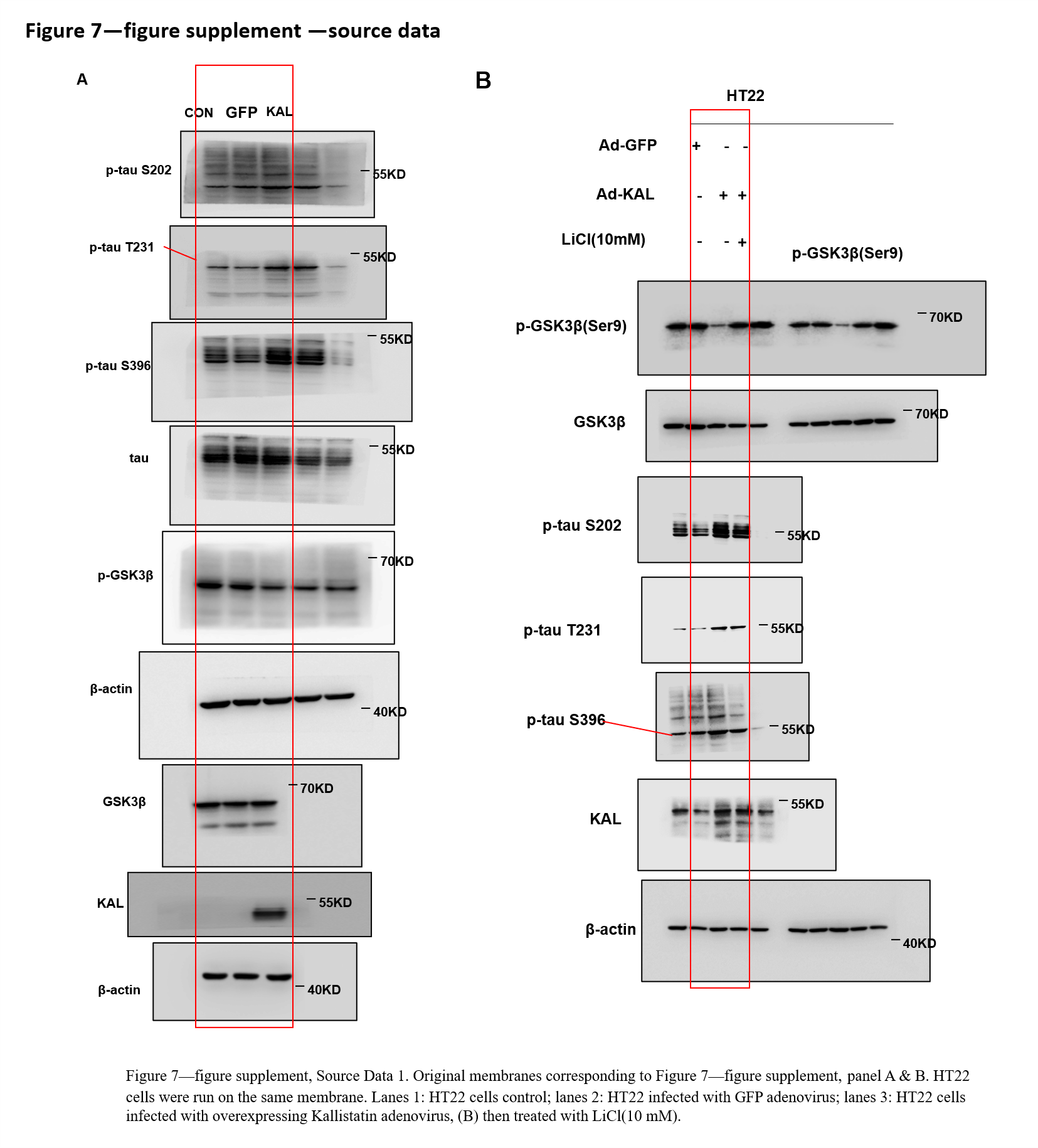

Supplement: Figure 7—figure supplement 1—source data 1. — Western blot analysis of GSK-3β, p-GSK-3β, tau, and p-tau (Ser9, T231, and S396) in HT22 cells infected with overexpressing Kallistatin adenovirus and control groups. [file elife-99462-fig7-figsupp1-data1.zip › Figure 7-figure supplement-source data 1/Figure 7-figure supplement-source data.png]

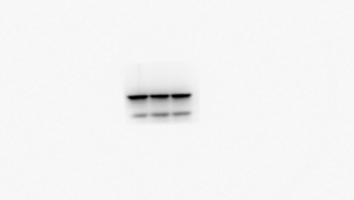

Supplement: Figure 7—figure supplement 1—source data 2. — Western blot analysis of GSK-3β, p-GSK-3β, and p-tau (Ser9, T231, and S396) in HT22 cells infected with overexpressing Kallistatin adenovirus and control groups for 24 hr, then treated with LiCl (10 mM) for 24 hr. [file elife-99462-fig7-figsupp1-data2.zip › Fig.S7A GSK3α╕åα╕ó HT22.tif]

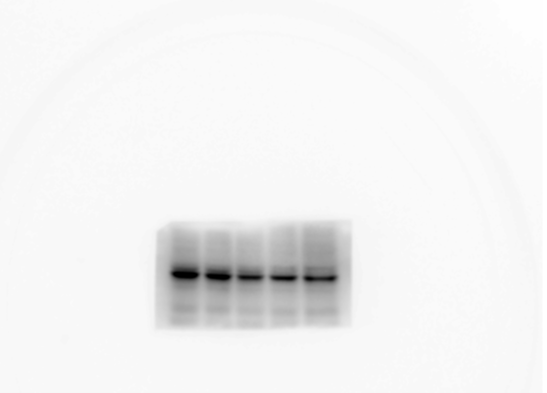

Supplement: Figure 7—figure supplement 1—source data 2. — Western blot analysis of GSK-3β, p-GSK-3β, and p-tau (Ser9, T231, and S396) in HT22 cells infected with overexpressing Kallistatin adenovirus and control groups for 24 hr, then treated with LiCl (10 mM) for 24 hr. [file elife-99462-fig7-figsupp1-data2.zip › Fig.S7A pGSK3α╕åα╕ó(S9) HT22.tif]

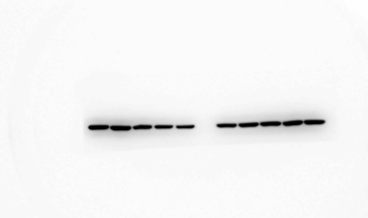

Supplement: Figure 7—figure supplement 1—source data 2. — Western blot analysis of GSK-3β, p-GSK-3β, and p-tau (Ser9, T231, and S396) in HT22 cells infected with overexpressing Kallistatin adenovirus and control groups for 24 hr, then treated with LiCl (10 mM) for 24 hr. [file elife-99462-fig7-figsupp1-data2.zip › Fig.S7B GSK3α╕åα╕ó HT22.tif]

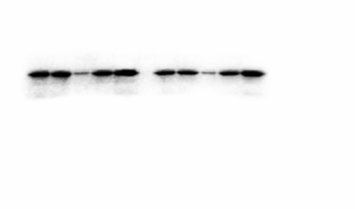

Supplement: Figure 7—figure supplement 1—source data 2. — Western blot analysis of GSK-3β, p-GSK-3β, and p-tau (Ser9, T231, and S396) in HT22 cells infected with overexpressing Kallistatin adenovirus and control groups for 24 hr, then treated with LiCl (10 mM) for 24 hr. [file elife-99462-fig7-figsupp1-data2.zip › Fig.S7B pGSK3α╕åα╕ó(S9) HT22.tif]

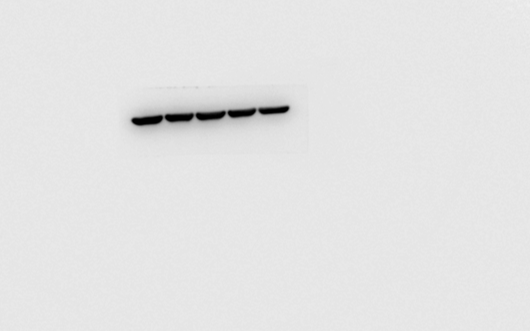

Supplement: Figure 7—figure supplement 1—source data 2. — Western blot analysis of GSK-3β, p-GSK-3β, and p-tau (Ser9, T231, and S396) in HT22 cells infected with overexpressing Kallistatin adenovirus and control groups for 24 hr, then treated with LiCl (10 mM) for 24 hr. [file elife-99462-fig7-figsupp1-data2.zip › Fig.S7A Actin HT22.tif]

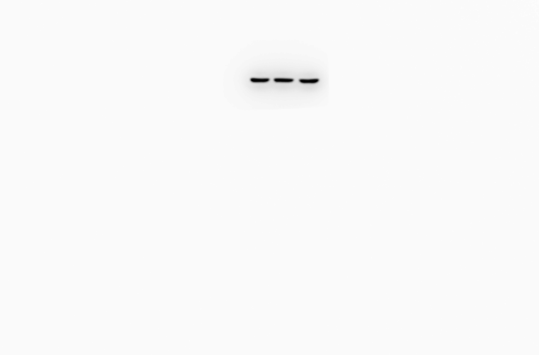

Supplement: Figure 7—figure supplement 1—source data 2. — Western blot analysis of GSK-3β, p-GSK-3β, and p-tau (Ser9, T231, and S396) in HT22 cells infected with overexpressing Kallistatin adenovirus and control groups for 24 hr, then treated with LiCl (10 mM) for 24 hr. [file elife-99462-fig7-figsupp1-data2.zip › Fig.S7A Actin2 HT22.tif]

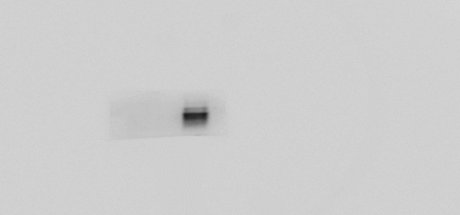

Supplement: Figure 7—figure supplement 1—source data 2. — Western blot analysis of GSK-3β, p-GSK-3β, and p-tau (Ser9, T231, and S396) in HT22 cells infected with overexpressing Kallistatin adenovirus and control groups for 24 hr, then treated with LiCl (10 mM) for 24 hr. [file elife-99462-fig7-figsupp1-data2.zip › Fig.S7A KAL HT22.tif]

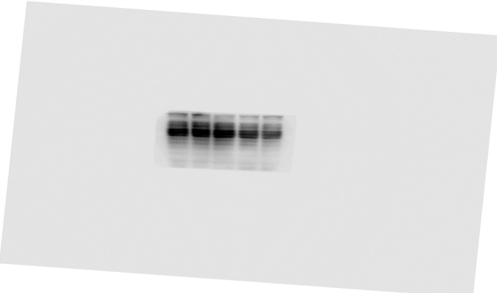

Supplement: Figure 7—figure supplement 1—source data 2. — Western blot analysis of GSK-3β, p-GSK-3β, and p-tau (Ser9, T231, and S396) in HT22 cells infected with overexpressing Kallistatin adenovirus and control groups for 24 hr, then treated with LiCl (10 mM) for 24 hr. [file elife-99462-fig7-figsupp1-data2.zip › Fig.S7A Tau HT22.tif]

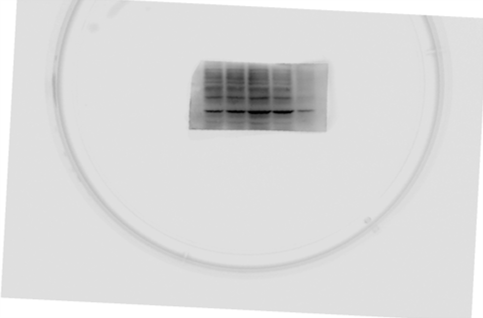

Supplement: Figure 7—figure supplement 1—source data 2. — Western blot analysis of GSK-3β, p-GSK-3β, and p-tau (Ser9, T231, and S396) in HT22 cells infected with overexpressing Kallistatin adenovirus and control groups for 24 hr, then treated with LiCl (10 mM) for 24 hr. [file elife-99462-fig7-figsupp1-data2.zip › Fig.S7A pTau(S202) HT22.tif]

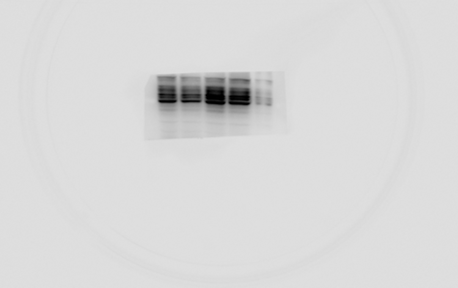

Supplement: Figure 7—figure supplement 1—source data 2. — Western blot analysis of GSK-3β, p-GSK-3β, and p-tau (Ser9, T231, and S396) in HT22 cells infected with overexpressing Kallistatin adenovirus and control groups for 24 hr, then treated with LiCl (10 mM) for 24 hr. [file elife-99462-fig7-figsupp1-data2.zip › Fig.S7A pTau(S396) HT22.tif]

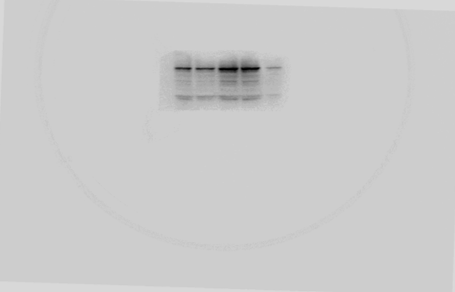

Supplement: Figure 7—figure supplement 1—source data 2. — Western blot analysis of GSK-3β, p-GSK-3β, and p-tau (Ser9, T231, and S396) in HT22 cells infected with overexpressing Kallistatin adenovirus and control groups for 24 hr, then treated with LiCl (10 mM) for 24 hr. [file elife-99462-fig7-figsupp1-data2.zip › Fig.S7A pTau(T231) HT22.tif]

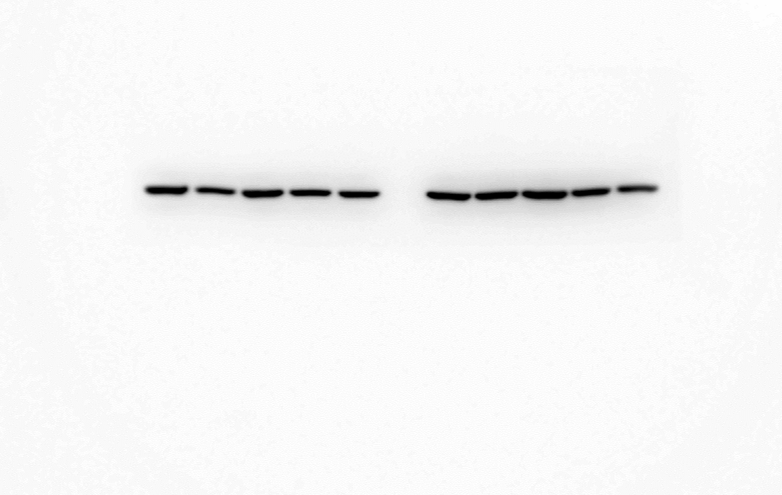

Supplement: Figure 7—figure supplement 1—source data 2. — Western blot analysis of GSK-3β, p-GSK-3β, and p-tau (Ser9, T231, and S396) in HT22 cells infected with overexpressing Kallistatin adenovirus and control groups for 24 hr, then treated with LiCl (10 mM) for 24 hr. [file elife-99462-fig7-figsupp1-data2.zip › Fig.S7B Actin HT22.tif]

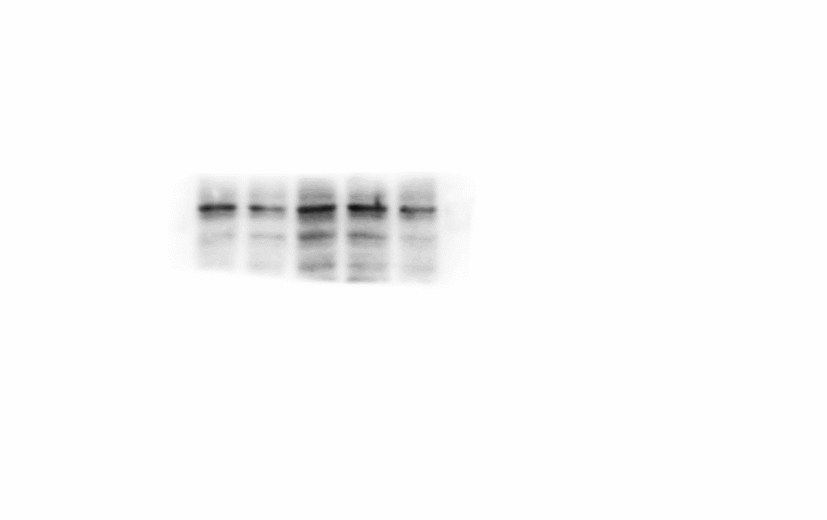

Supplement: Figure 7—figure supplement 1—source data 2. — Western blot analysis of GSK-3β, p-GSK-3β, and p-tau (Ser9, T231, and S396) in HT22 cells infected with overexpressing Kallistatin adenovirus and control groups for 24 hr, then treated with LiCl (10 mM) for 24 hr. [file elife-99462-fig7-figsupp1-data2.zip › Fig.S7B KAL HT22.tif]

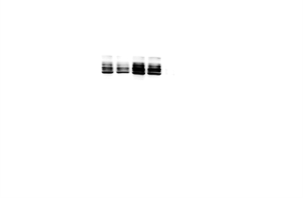

Supplement: Figure 7—figure supplement 1—source data 2. — Western blot analysis of GSK-3β, p-GSK-3β, and p-tau (Ser9, T231, and S396) in HT22 cells infected with overexpressing Kallistatin adenovirus and control groups for 24 hr, then treated with LiCl (10 mM) for 24 hr. [file elife-99462-fig7-figsupp1-data2.zip › Fig.S7B pTau(S202) HT22.tif]

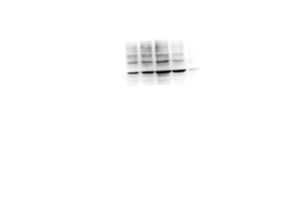

Supplement: Figure 7—figure supplement 1—source data 2. — Western blot analysis of GSK-3β, p-GSK-3β, and p-tau (Ser9, T231, and S396) in HT22 cells infected with overexpressing Kallistatin adenovirus and control groups for 24 hr, then treated with LiCl (10 mM) for 24 hr. [file elife-99462-fig7-figsupp1-data2.zip › Fig.S7B pTau(S396) HT22.tif]

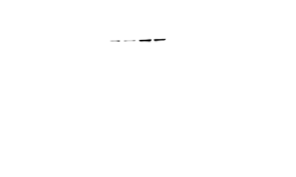

Supplement: Figure 7—figure supplement 1—source data 2. — Western blot analysis of GSK-3β, p-GSK-3β, and p-tau (Ser9, T231, and S396) in HT22 cells infected with overexpressing Kallistatin adenovirus and control groups for 24 hr, then treated with LiCl (10 mM) for 24 hr. [file elife-99462-fig7-figsupp1-data2.zip › Fig.S7B pTau(T231) HT22.tif]

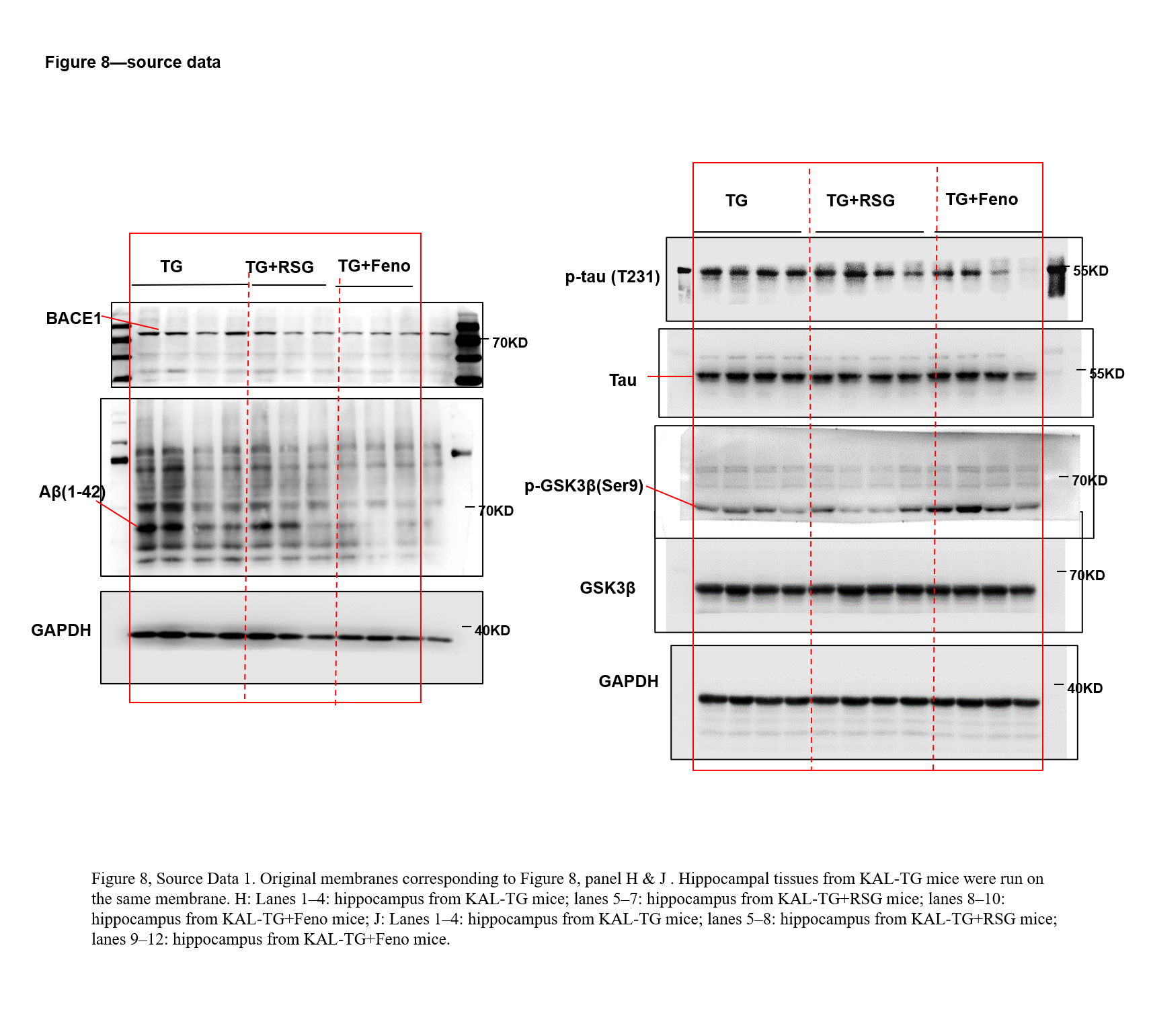

Supplement: Figure 8—source data 1. — Protein levels of Aβ and BACE1 were tested by western blot analysis in hippocampal tissue. [file elife-99462-fig8-data1.zip › Figure 8-source data 1/Figure 8-source data.png]

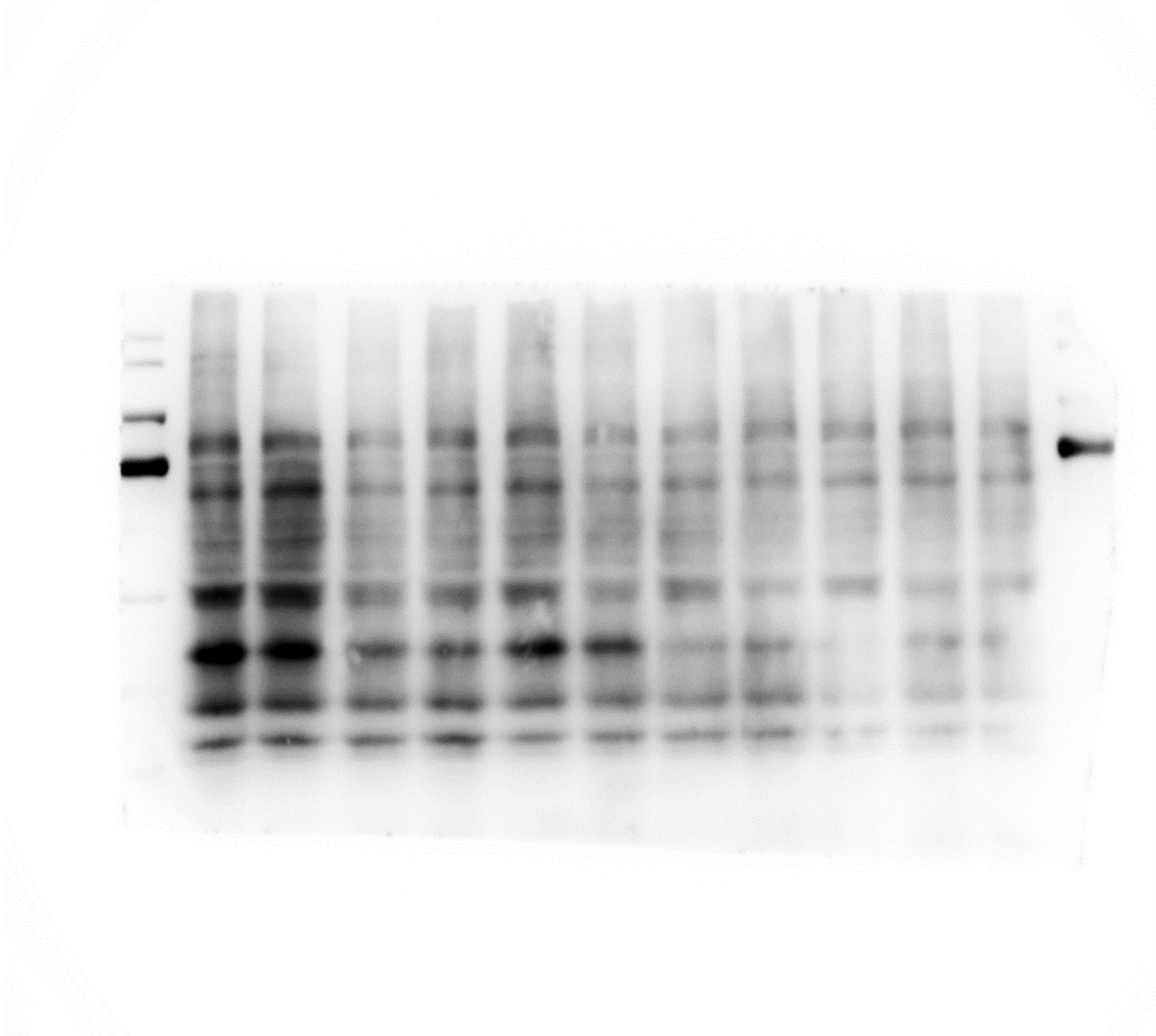

Supplement: Figure 8—source data 2. — Protein levels of p-tau (231), tau, p-GSK-3β (Ser9), and GSK-3β were tested by western blot analysis in hippocampal tissue. [file elife-99462-fig8-data2.zip › Fig.8H Aα╕åα╕ó(1-42) hippo.tif]

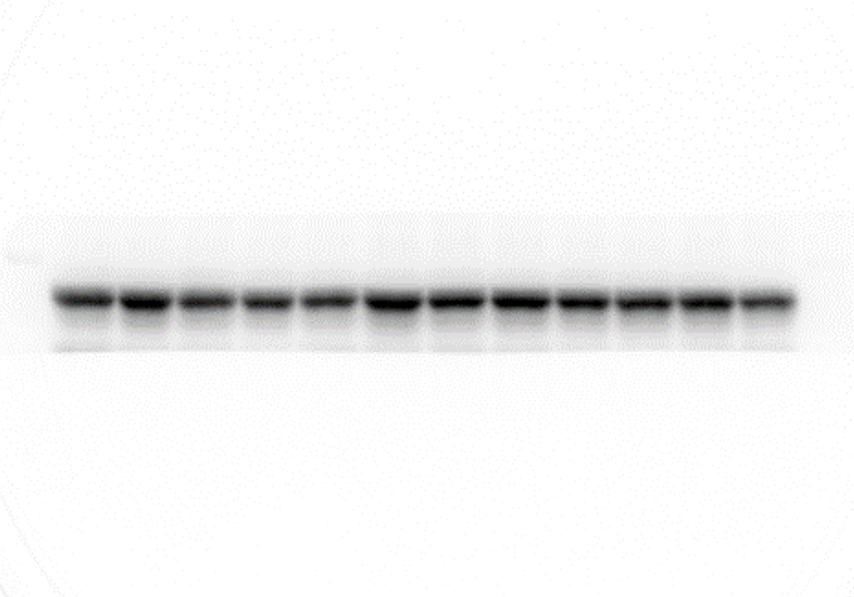

Supplement: Figure 8—source data 2. — Protein levels of p-tau (231), tau, p-GSK-3β (Ser9), and GSK-3β were tested by western blot analysis in hippocampal tissue. [file elife-99462-fig8-data2.zip › Fig.8J GSK3α╕åα╕ó hippo.tif]

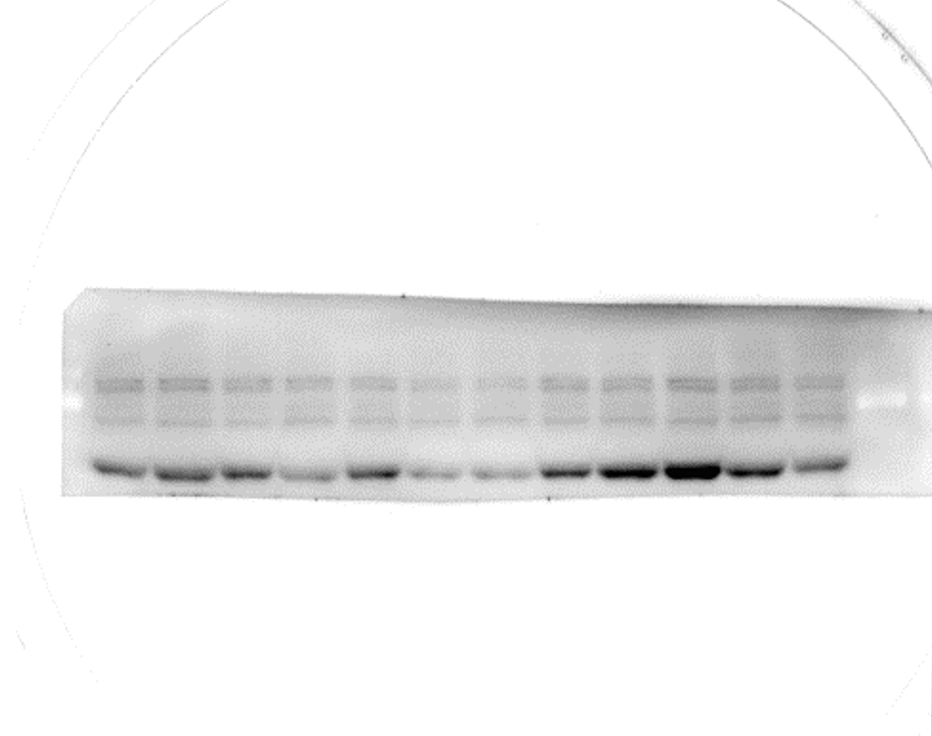

Supplement: Figure 8—source data 2. — Protein levels of p-tau (231), tau, p-GSK-3β (Ser9), and GSK-3β were tested by western blot analysis in hippocampal tissue. [file elife-99462-fig8-data2.zip › Fig.8J pGSK3α╕åα╕ó(S9) hippo.tif]

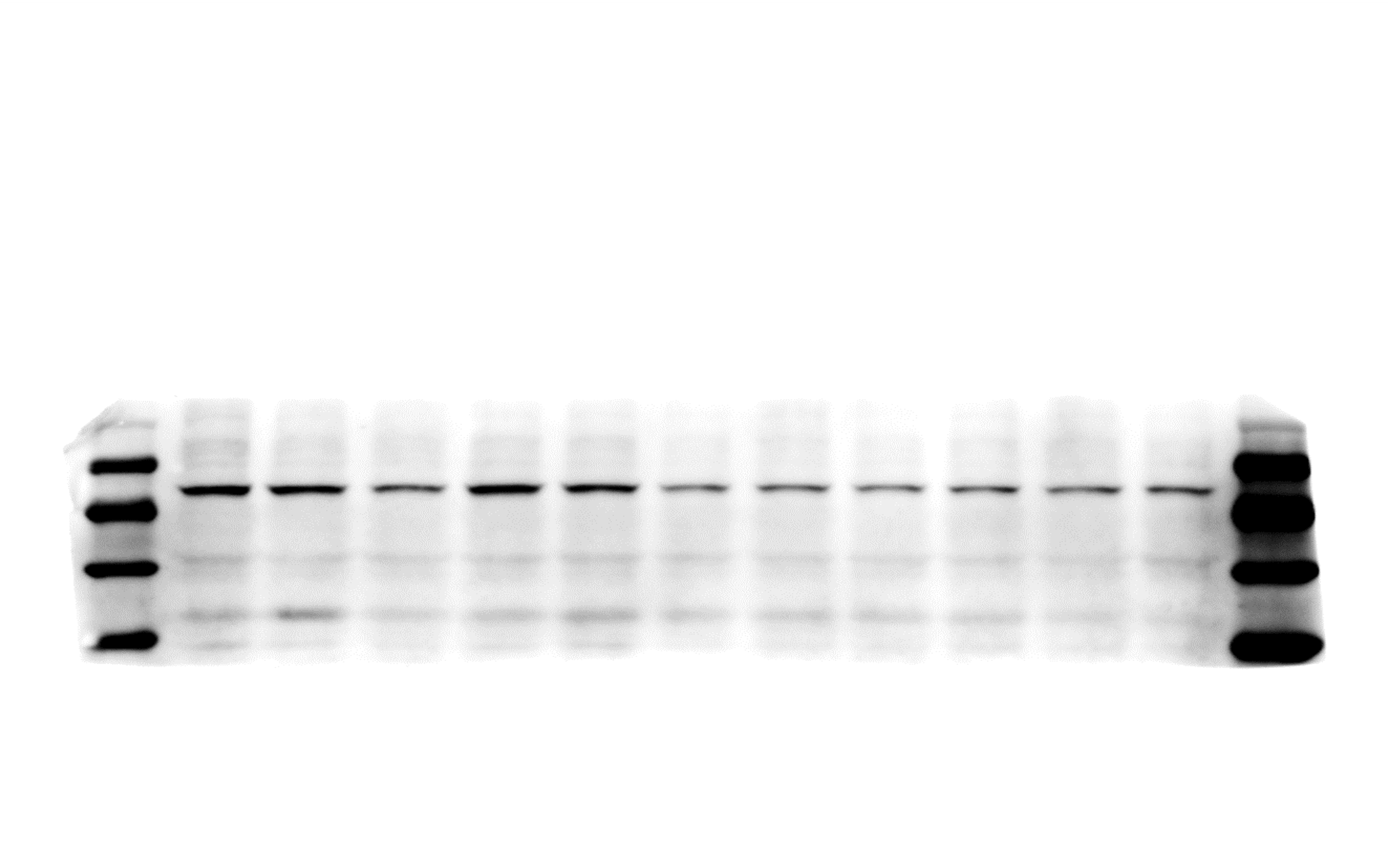

Supplement: Figure 8—source data 2. — Protein levels of p-tau (231), tau, p-GSK-3β (Ser9), and GSK-3β were tested by western blot analysis in hippocampal tissue. [file elife-99462-fig8-data2.zip › Fig.8H BACE1 hippo.tif]

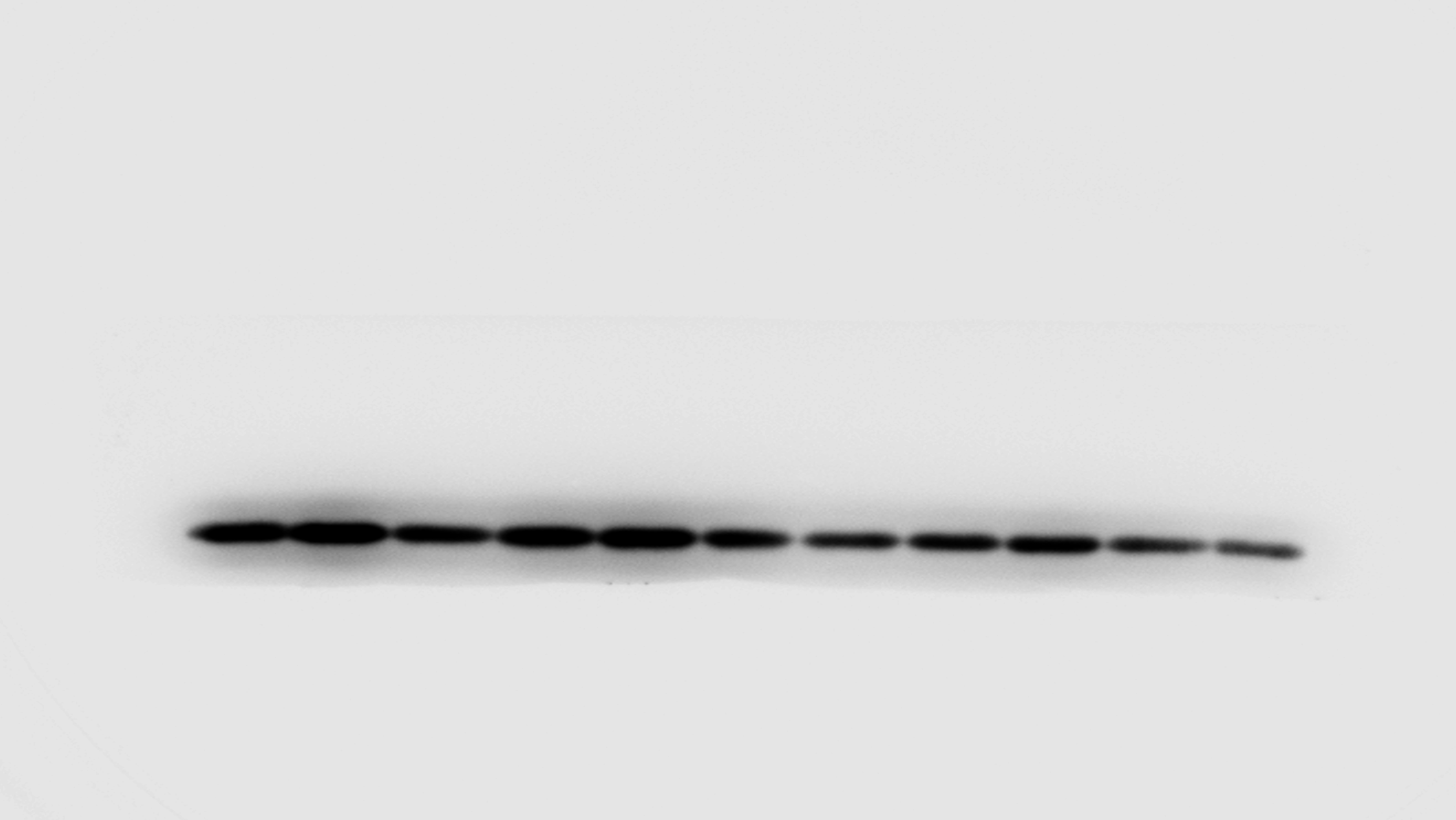

Supplement: Figure 8—source data 2. — Protein levels of p-tau (231), tau, p-GSK-3β (Ser9), and GSK-3β were tested by western blot analysis in hippocampal tissue. [file elife-99462-fig8-data2.zip › Fig.8H GAPDH hippo.tif]

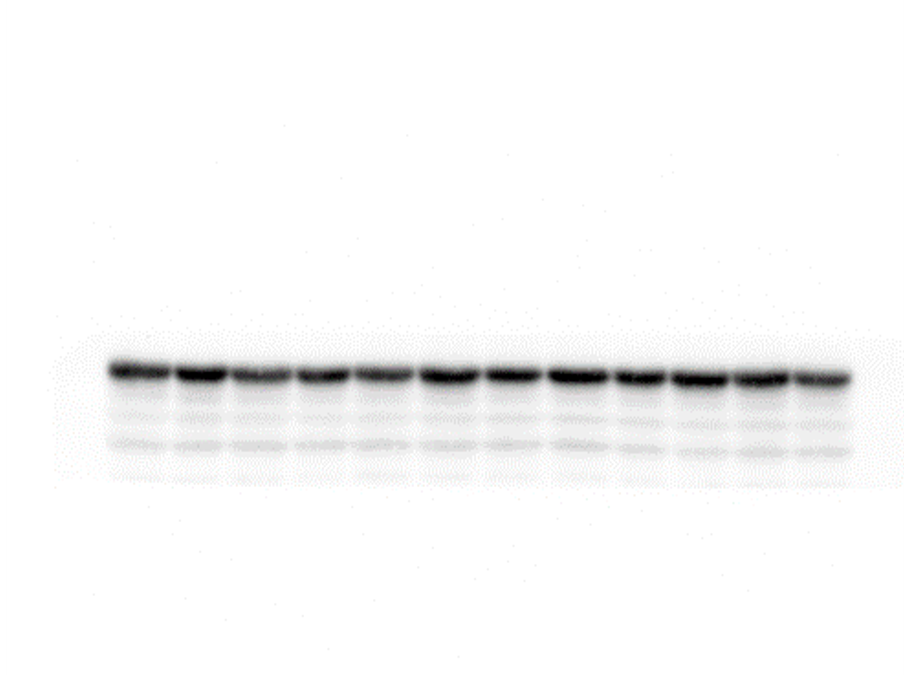

Supplement: Figure 8—source data 2. — Protein levels of p-tau (231), tau, p-GSK-3β (Ser9), and GSK-3β were tested by western blot analysis in hippocampal tissue. [file elife-99462-fig8-data2.zip › Fig.8J GAPDH hippo.tif]

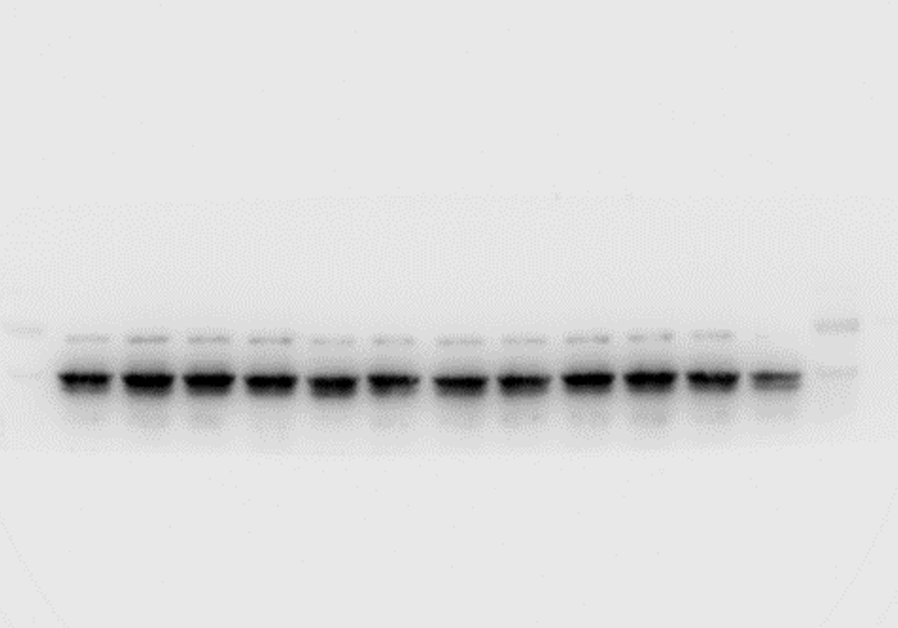

Supplement: Figure 8—source data 2. — Protein levels of p-tau (231), tau, p-GSK-3β (Ser9), and GSK-3β were tested by western blot analysis in hippocampal tissue. [file elife-99462-fig8-data2.zip › Fig.8J Tau hippo.tif]

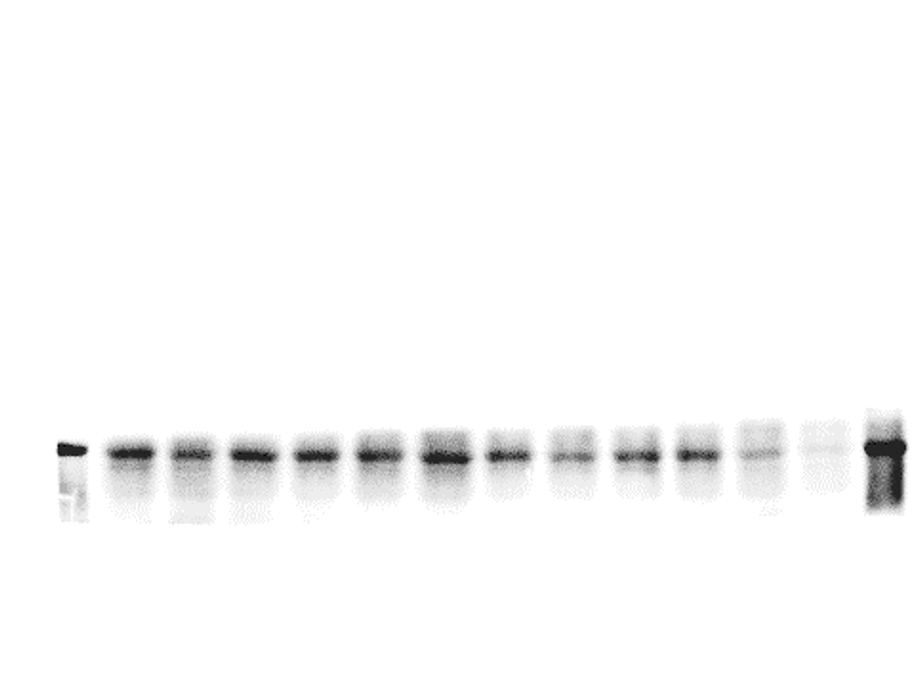

Supplement: Figure 8—source data 2. — Protein levels of p-tau (231), tau, p-GSK-3β (Ser9), and GSK-3β were tested by western blot analysis in hippocampal tissue. [file elife-99462-fig8-data2.zip › Fig.8J pTau(T231) hippo.tif]
